# Preclinical candidate for the treatment of visceral leishmaniasis that acts through proteasome inhibition

**DOI:** 10.1073/pnas.1820175116

**Published:** 2019-04-08

**Authors:** Susan Wyllie, Stephen Brand, Michael Thomas, Manu De Rycker, Chun-wa Chung, Imanol Pena, Ryan P. Bingham, Juan A. Bueren-Calabuig, Juan Cantizani, David Cebrian, Peter D. Craggs, Liam Ferguson, Panchali Goswami, Judith Hobrath, Jonathan Howe, Laura Jeacock, Eun-Jung Ko, Justyna Korczynska, Lorna MacLean, Sujatha Manthri, Maria S. Martinez, Lydia Mata-Cantero, Sonia Moniz, Andrea Nühs, Maria Osuna-Cabello, Erika Pinto, Jennifer Riley, Sharon Robinson, Paul Rowland, Frederick R. C. Simeons, Yoko Shishikura, Daniel Spinks, Laste Stojanovski, John Thomas, Stephen Thompson, Elisabet Viayna Gaza, Richard J. Wall, Fabio Zuccotto, David Horn, Michael A. J. Ferguson, Alan H. Fairlamb, Jose M. Fiandor, Julio Martin, David W. Gray, Timothy J. Miles, Ian H. Gilbert, Kevin D. Read, Maria Marco, Paul G. Wyatt

**Affiliations:** ^a^Drug Discovery Unit, Wellcome Centre for Anti-Infectives Research, Division of Biological Chemistry and Drug Discovery, School of Life Sciences, University of Dundee, Dundee DD1 5EH, United Kingdom;; ^b^Medicines Research Centre, Stevenage, Hertfordshire SG1 2NY, United Kingdom;; ^c^Global Health R&D, GlaxoSmithKline, Tres Cantos, 28760, Spain;; ^d^David Jack Centre for R&D, GlaxoSmithKline, Ware SG12 0DP, United Kingdom

**Keywords:** Leishmania, proteasome, cryo-EM, drug discovery

## Abstract

Visceral leishmaniasis (VL), caused by the protozoan parasites *Leishmania donovani* and *Leishmania infantum*, is one of the major parasitic diseases worldwide. There is an urgent need for new drugs to treat VL, because current therapies are unfit for purpose in a resource-poor setting. Here, we describe the development of a preclinical drug candidate, GSK3494245/DDD01305143/compound 8, with potential to treat this neglected tropical disease. The compound series was discovered by repurposing hits from a screen against the related parasite *Trypanosoma cruzi*. Subsequent optimization of the chemical series resulted in the development of a potent cidal compound with activity against a range of clinically relevant *L. donovani* and *L. infantum* isolates. Compound 8 demonstrates promising pharmacokinetic properties and impressive in vivo efficacy in our mouse model of infection comparable with those of the current oral antileishmanial miltefosine. Detailed mode of action studies confirm that this compound acts principally by inhibition of the chymotrypsin-like activity catalyzed by the β5 subunit of the *L. donovani* proteasome. High-resolution cryo-EM structures of apo and compound 8-bound *Leishmania tarentolae* 20S proteasome reveal a previously undiscovered inhibitor site that lies between the β4 and β5 proteasome subunits. This induced pocket exploits β4 residues that are divergent between humans and kinetoplastid parasites and is consistent with all of our experimental and mutagenesis data. As a result of these comprehensive studies and due to a favorable developability and safety profile, compound 8 is being advanced toward human clinical trials.

## Significance

Safer and more effective oral drugs are urgently required to treat visceral leishmaniasis (VL), a neglected parasitic disease that kills 20,000–40,000 people each year in parts of Asia, Africa, and Latin America. Here, we describe the development of GSK3494245/DDD01305143/compound 8, a small molecule that demonstrates clinical-level efficacy in a mouse model of VL. Compound 8 exhibits attractive biological and biosafety properties, resulting in its selection as a preclinical candidate. Target deconvolution and cryo-EM studies reveal that compound 8 is a potent and selective inhibitor of the chymotrypsin-like activity of the parasite proteasome binding in a site sandwiched between the β4 and β5 subunits. Compound 8 is progressing toward human clinical trials, raising hopes of improved therapeutics for this disease.

Visceral leishmaniasis (VL), the most serious form of leishmaniasis, is invariably fatal if left untreated (1). This neglected tropical disease is caused by infection with the protozoan parasite *Leishmania donovani* or *Leishmania infantum*. There are over 600 million people at risk from infection, and the annual death toll is 20,000–40,000 (2). The disease is spread through the bite of infected sandflies, giving rise to a systemic infection in the human host with diverse symptoms, including fever, weight loss, anemia, and hepatosplenomegaly. More than 90% of VL cases are reported in India, Sudan, southern Sudan, Ethiopia, Kenya, Somalia, and Brazil; however, this disease has a worldwide presence, with cases in Asia, East Africa, South America, and the Mediterranean region (3). HIV/VL coinfections are commonplace and can accelerate the progression of both diseases (4). Significantly, VL-associated morbidity has a considerable economic impact, contributing to a perpetual cycle of poverty in some of the poorest regions of the world.

Current available treatments for VL are limited to pentavalent antimonials, amphotericin B, paromomycin, and miltefosine, the only oral treatment available. These treatments have serious drawbacks, including prolonged treatment duration (20–30 d for antimonials and 28 d for miltefosine and amphotericin B deoxycholate), parenteral administration (amphotericin B, antimonials, and paromomycin), low tolerability (antimonials and amphotericin B deoxycholate), teratogenicity (miltefosine), treatment failures (5, 6) (paromomycin, miltefosine, and antimonials), and requirement for cold storage and high cost (liposomal amphotericin B). These drugs also show significant geographical variation in effectiveness for reasons that are poorly understood. In particular, none of the current VL drugs have high levels of efficacy in East Africa (7, 8). Thus, identification of a low-cost, safe, effective, oral, and short-course drug for VL is urgently needed (9). Here, we report the discovery of a preclinical candidate for the treatment of VL that acts principally by inhibition of the chymotrypsin-like activity catalyzed by the β5 subunit of the *L. donovani* proteasome.

## Results and Discussion

### Discovery of Compound 8.

Identification of new chemical entities capable of treating kinetoplastid infections has proven to be extremely challenging. There are few robustly validated drug targets in the kinetoplastid parasites, making target-based approaches speculative (10), and drug discovery programs are usually reliant on phenotypic screening to find suitable chemical start points. The identification of compounds with activity against these intracellular parasites has also proven difficult, particularly in the case of *L. donovani*, where the mammalian stage of the parasite, the amastigote, resides within human macrophages. High-throughput screening against the intramacrophage stage of the parasite is plagued by extremely low hit rates; however, activity for a compound series in this assay usually translates into activity in animal models of disease if pharmacokinetic properties can be optimized (11, 12).

**Fig. 1.**
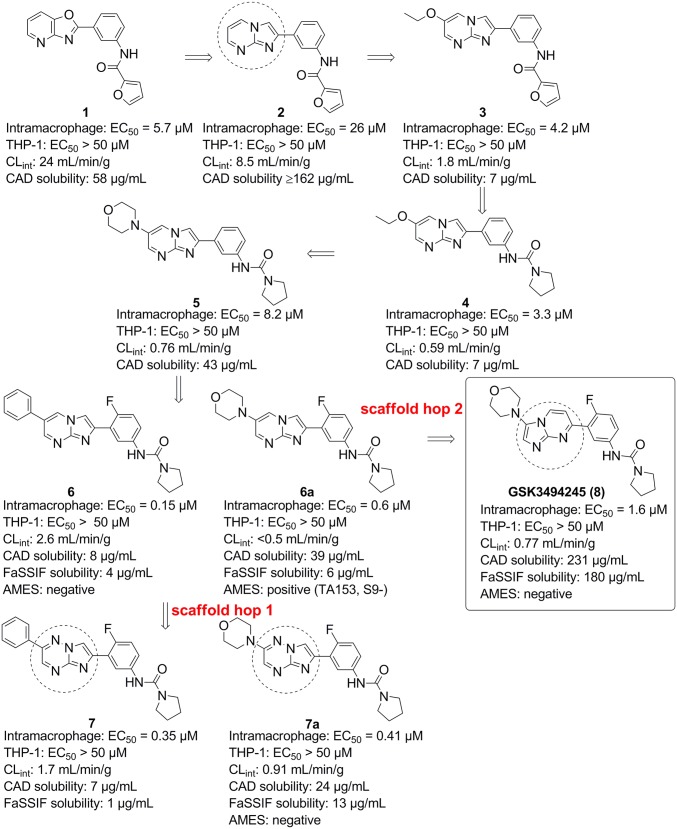
The evolution of the series from hits to **8**. Potencies against intramacrophage amastigotes and against THP-1 cells are shown. Data are from at least three independent replicates. CAD solubility, charged aerosol detector solubility.

The hit compound **1** was identified from phenotypic screening of a 15,659-compound diversity library against the related kinetoplastid parasite *Trypanosoma cruzi* [EC_50_ = 0.22 µM; 95% confidence interval (95% CI) = 0.087–0.36 µM; *n* = 3] (*SI Appendix*, Fig. S1 shows the screening progression cascade). An initial scaffold hop led to **2**, which maintains activity against *T. cruzi* (Fig. 1) (EC_50_ = 0.93 µM; 95% CI = 0.47–1.4 µM; *n* = 4). We hypothesized that, in view of their structural similarity, **1** and **2** probably shared the same mechanism of action. Screening both compounds in our *L. donovani* intramacrophage assay, where the amastigotes are cultured in differentiated THP-1 cells (13), gave EC_50_ values for compound **1** of 5.7 μM (95% CI = 2.3–14 μM; *n* = 5) and for compound **2** of 26 μM (95% CI = 13–52 μM; *n* = 4). Compounds **1** and **2** also demonstrated good selectivity over mammalian cell growth inhibition (THP-1 cells; EC_50_ > 50 μM), although both did show poor in vitro metabolic stability as shown by their rapid degradation when incubated with mouse liver microsomes (Fig. 1). Compound **2** showed improved kinetic solubility, and therefore, we decided to focus on the imidazopyrimidine scaffold for additional optimization.

The poor in vitro metabolic stability was improved by substituting at the six position of the pyrimidine ring as exemplified by **3** [intramacrophage EC_50_ value of 4.2 μM; 95% CI = 1.5–12 μM; *n* = 4; intrinsic clearance when incubated with mouse liver microsomes (CL_int_) = 1.8 mL/min per gram]. The furan group is potentially metabolically liable, and if metabolized, it could give rise to reactive intermediates. It proved possible to replace the furan amide with a pyrrolidinyl urea (**4**), which maintained potency against the parasite (EC_50_ = 3.3 μM; 95% CI = 1.4–7.6 μM; *n* = 4; CL_int_ = 0.59 mL/min per gram). Replacement of the 6-ethoxy substituent by a morpholine gave **5**, with good metabolic stability (CL_int_ = 0.76 mL/min per gram) and improved solubility (charged aerosol detector = 43 µg/mL), although with a slight decrease in its antileishmanial activity (EC_50_ = 8.2 μM; 95% CI = 5.4–11 μM; *n* = 3). Addition of fluorine to the four position of the phenyl ring to give **6a** improved potency by >10-fold (EC_50_ = 0.6 µM; 95% CI = 0.35–1.1 μM; *n* = 5). Potency could be slightly enhanced by replacement of morpholine with phenyl to give **6** (EC_50_ = 0.15 µM; 95% CI = 0.1–0.23 μM; *n* = 7). Compound **6a** was shown to be positive in an Ames assay, indicating a genotoxic liability, and therefore, we attempted to develop compounds that were negative in the Ames assay. Furthermore, **6** and **6a** had poor solubility in the biorelevant fasted simulated intestinal fluid (FaSSIF) assay.

A key strategy to tackle genotoxicity and also improve solubility was to replace the core bicycle via scaffold hopping (14). This involved varying the position and number of heteroatoms in the bicyclic system as well as switching the left-hand six-membered and right-hand five-membered rings. This led to two scaffolds that were selected for additional evaluation: imidazo[1,2-b][1,2,4]triazines (exemplified by **7** and **7a**) and imidazo[1,2-a]pyrimidines (exemplified by compound **8**). The phenyl analog **7** gave an EC_50_ value of 0.35 μM (95% CI = 0.25–0.5 μM; *n* = 9), and **7a** returned an EC_50_ of 0.41 μM (95% CI = 0.35–0.47 μM; *n* = 49) in the intramacrophage assay. Unfortunately, the aniline resulting from the removal of the urea moiety in **7a** was positive in an Ames assay, and additional development was stopped; however, the high potency of compound **7** led to it being selected as a tool compound for mechanism of action studies. The imidazo[1,2-a]pyrimidine (**8**) showed slightly lower potency with EC_50_ of 1.6 μM (95% CI = 1.1–2.2 μM; *n* = 11), but this was balanced by improved FaSSIF solubility (180 µg/mL). It showed good in vitro metabolic stability (CL_int_ = 0.8 mL/min per gram) and selectivity over mammalian cells. Both **8** and the corresponding free aniline gave negative results in the Ames assay. All of these factors led to the selection of **8** for more detailed profiling.

Due to the global spread of the disease, future antileishmanial drugs must demonstrate efficacy against a diverse range of clinical strains from geographical locations (15). This was found to be the case for **8**, which maintained potency in vitro against clinical strains (EC_50_ ∼ 1 μM), including two strains isolated in East Africa (LV9 and SUKA001) and two strains from India (DD8-WT and BHU1), with the latter being antimony resistant (*SI Appendix*, Table S1). To gain insight into the concentration and duration of treatment required to kill the parasites, **8** was assessed in our well-established axenic amastigote rate-of-kill assay (16). Compound **8** induced parasite cell death at a minimum concentration of 620 nM after 72 h. As compound concentrations were increased, the time to kill was reduced to 48 h (*SI Appendix*, Fig. S2).

### In Vivo Efficacy and PK.

**Fig. 2.**
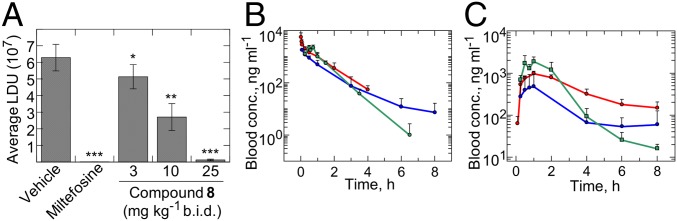
In vivo efficacy and blood exposure of **8**. (*A*) Therapeutic efficacy of **8** in a mouse model of VL (*L. donovani*, LV9). Data correspond to mean Leishman Donovan units (LDUs) for mice treated with **8**, miltefosine, or vehicle alone (*n* = 5). Unpaired *t* tests confirmed that the reduction in parasite burden evident in all treated mice is statistically significant compared with untreated control animals, with *P = 0.0441 and **P = 0.0037 for mice treated with compound **8** at 3 and 10 mg/kg, respectively, and *P* < 0.0001 for mice treated with miltefosine and compound **8** at 25 mg/kg. Blood levels of **8** after a single i.v. dose at 3 mg/kg (*B*) or a single oral dose at 10 mg/kg (*C*) to male CD-1 mice (blue), male SD rats (red), and male beagle dogs (green). The profiles are represented as mean ± SD. The *y* axis is represented as a logarithmic scale. b.i.d., twice a day.

Given its promising in vitro profile, **8** was progressed to a mouse model of VL infection (17). Initial pharmacokinetic studies in mice indicated that the compound had sufficient oral bioavailability to obtain free compound levels in excess of EC_99_ values when dosed twice daily at 25 mg/kg. Infected mice were dosed at 3, 10, or 25 mg/kg orally twice a day for 10 consecutive days. Two days after the termination of treatment, mice were euthanized, and the parasite burdens in their livers were determined. When dosed orally at 25 mg/kg, compound **8** elicited a >95% reduction of parasite load, similar efficacy to that seen with miltefosine dosed orally at 30 mg/kg once daily in the same model (Fig. 2 and *SI Appendix*, Table S2). In this dose-ranging experiment, we demonstrated that the ED_50_, ED_90_, and ED_99_ values for compound **8** after 10-d treatments orally twice a day were 8.9, 16, and 30 mg/kg, respectively. In comparison, the reported ED_90_ for miltefosine in a similar mouse model was 27 mg/kg once daily (18).

The PK properties of **8** are such that it can be orally dosed to reach efficacious levels in a range of preclinical species, including mouse, rat, and dog (*SI Appendix*, Table S3). Relatively low oral bioavailability (*F*) was observed in mouse (*F* = 18%), although bioavailability was moderate in rat and dog (*F* = 35 and 46%, respectively). The volume of distribution at steady state indicated that the compound had distribution beyond the bloodstream (2.2, 1.0, and 1.3 L/kg in mouse, rat, and dog, respectively). Compound **8** had moderate blood clearance in mice and dogs (41 and 30 mL/min per kilogram, respectively) and low clearance in rat (17 mL/min per kilogram).

### Safety Profile.

Compound **8** has exhibited no noteworthy safety liabilities at any dose tested during extensive in vitro profiling, including genotoxicity (Ames and mouse lymphoma tests) (*SI Appendix*, Tables S4 and S5). After a rat 7-d toxicology study (at doses up to 300 mg/kg), no significant safety or tolerability liabilities were detected. Thus, **8** is expected to have a safety margin of at least 37-fold by comparing the exposure in the rat at 300 mg/kg (980 μg/h per mililiter) to the observed exposure giving >95% parasite burden reduction (27 μg/h per mililiter when **8** was dosed at 25 mg/kg twice a day for 10 days).

Overall, **8** is a small molecule with good physicochemical properties that is highly efficacious (in both in vitro and in vivo assays), demonstrates a desirable safety profile, and shows balanced PK properties. As a result of these promising findings, **8** was selected as a preclinical candidate and is now being progressed toward human clinical trials.

### Mode of Action Studies.

Elucidating the mode of action of novel chemical series can be enormously beneficial in drug discovery campaigns. Since there is no roadmap establishing the mode of action of bioactive small molecules, several complementary methodologies were used with representative analogs of this compound series as chemical tools.

### Whole-Genome RNAi Library Screening.

**Fig. 3.**
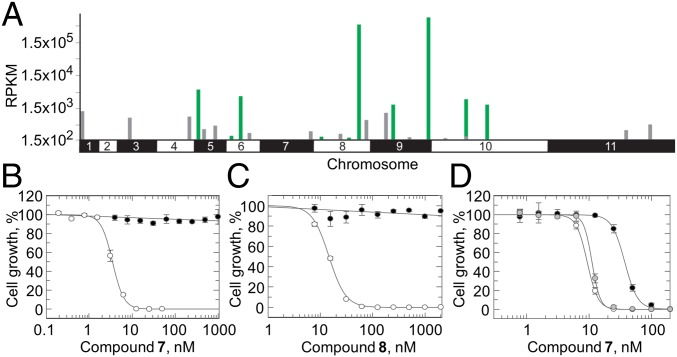
Target identification and validation studies in *T. brucei* and *L. donovani*. (*A*) Genome-wide map indicating RITseq hits from screening of **7**. Multiple RITseq fragments represent primary hits and are indicated in green. Other loci with mapped reads are indicated in gray. RPKM, reads per kilobase of transcript per million mapped reads. (*B*) Dose–response curves for WT (white circles) and RES II-resistant cells (black circles) treated with **7**. EC_50_ values of 3.5 ± 0.09 and >1,000 nM were determined for WT and RES II cells, respectively. (*C*) EC_50_ values for WT (white circles) and RES II-resistant cells (black circles) treated with **8** were 14.6 ± 0.3 and >1,000 nM, respectively. (*D*) EC_50_ values for WT (white), β5MUT (black), and β4MUT cells (gray) treated with **7** were 9.4 ± 0.14, 37.6 ± 0.6, and 11.2 ± 0.2 nM, respectively. All dose–response curves are the nonlinear regression fits using a two-parameter EC_50_ equation. Data are the mean ± SD of at least two independent experiments.

Initially, our studies of the mechanism of action of this compound series focused primarily on compound **7**. Compounds from this series, including **7**, are equally potent against both *L. donovani* and *Trypanosoma brucei*, enabling us to utilize our genome-wide RNAi (RITseq) library in *T. brucei* (19) and suggesting that this series of compounds could exhibit a broad spectrum of antikinetoplastid activity. The library was exposed to a typically lethal dose (three times the EC_50_ value) of **7**. Under tetracycline induction, each trypanosome in the library produces dsRNA from integrated RNAi target fragments (Fig. 3*A*). The resulting target knockdown has the potential to confer a selective advantage under drug pressure. RITseq was used to generate a readout from the parasite population that tolerated **7**. Screening of **7** against the RITseq library identified 10 “hits” with functional domains commonly found in proteins of the ubiquitin–proteasome recycling pathway (Fig. 3*A* and *SI Appendix*, Table S7). The top two hits from these screening studies (Tb927.9.15260 and Tb927.8.6620) were further investigated, and individual stem-loop RNAi studies confirmed that knockdown of the RNA transcripts associated with these genes conferred (approximately twofold) resistance to compound **7** (*SI Appendix*, Figs. S3 and S4). We suggest that knockdown of nonessential ubiquitin proteasome activity increases capacity and flux through ubiquitin proteasome pathways that are essential for viability. Collectively, these data implicated the ubiquitin proteasome system in mechanism(s) of resistance and potentially, mechanism(s) of action of compounds from this series.

### Resistance Generation.

*L. donovani* cell lines resistant to **7** were generated in vitro by exposing promastigotes to stepwise increasing concentrations of compound. The resulting clones demonstrated >100-fold resistance to **7** (Fig. 3*B*) and were also cross-resistant to all of the compounds from this series that were tested, including **8** (Fig. 3*C*), confirming that compounds from this series are likely to share a similar mechanism of action. At this point, Khare et al. (20) published their studies of GNF6702, a structurally related compound that selectively inhibits the kinetoplastid proteasome. They carried out mode of action studies in *T. cruzi* and identified the proteasome as the target of their inhibitor, suggesting that the compound acted at the interface of the β4 and β5 subunits. Guided by these studies and by the results of our own RITseq library screen, we carried out targeted DNA sequencing of the genes encoding the subunits of the *L. donovani* proteasome. Sequencing of three independently generated resistant clones revealed homozygous mutations within the genes encoding the β4 and β5 subunits. Specifically, clones RES I and II maintained mutations encoding a G197C substitution in the β5 subunit as well as a T30A substitution in the β4 subunit. RES III maintained solely a G197S substitution in the β5 subunit.

To determine the role of these mutations in resistance to **7** and other compounds from this series, we engineered *L. donovani* promastigote lines that overexpressed either β5^G197C^ or β4^T30A^. Overexpression of the mutated β5 subunit reduced the susceptibility of WT parasites to **7** by fourfold, confirming the role of this mutation in resistance (Fig. 3*D*). However, overexpression of β4^T30A^ did not alter susceptibility to compounds from this series. These data are entirely consistent with our compounds targeting the proteasome of *L. donovani*, specifically the chymotrypsin-like degradation activity catalyzed by the β5 subunit.

### Inhibition of the Chymotrypsin-Like Activity of the *L. donovani* Proteasome.

**Fig. 4.**
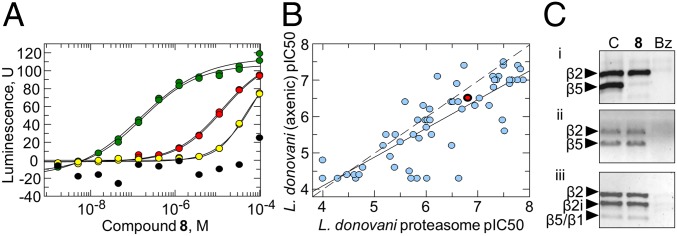
Target validation studies in *L. donovani*. (*A*) Dose–response curves determining the effect of **8** on chymotrypsin-like proteasome activity in lysates of *L. donovani* (green), purified human 26S proteasomes (red), extracts of THP-1 monocytes (yellow), and the absence of proteasome (black). (*B*) A correlation plot of cellular potency (*L. donovani* axenic amastigotes; *x* axis) and biochemical inhibition of *Leishmania* proteasome chymotrypsin-like activity (*y* axis) for a set of compound **8** analogs. Compound **8** is shown in red. (*C*) The proteasome active site probe UbiQ-018 fluorescently and covalently labels the β subunits of the proteasome in extracts of *L. donovani* WT (*i*), *L. donovani*-resistant RES II cells (*ii*), and human THP-1 monocytes (*iii*). At 100 µM, compound **8** selectively blocks labeling of β5 in *L. donovani* (WT) but not in THP-1 extracts or extracts from compound **7**-resistant *L. donovani*. The presence of the panproteasome inhibitor bortezomib (Bz) prevents labeling of proteasome subunits in all extracts. Subunit identity was assigned by mass spectrometry of the bands excised from the gel (details are in *SI Appendix*, *SI Text* and Table S8).

An in vitro assay was developed that enabled the chymotrypsin-like activity and additional proteolytic activities associated with the proteasome to be directly monitored within proteasome-enriched parasite cell lysates. Likewise, human proteasome from two sources (i.e., commercially pure 26S and enriched proteasome fractions from THP-1 lysates) was tested using the same biochemical assays. Compound **8** specifically inhibited the chymotrypsin-like activity of the *L. donovani* proteasome in a dose-dependent manner (mean IC_50_ = 0.16 ± 0.01 µM) (Fig. 4*A*) but had no effect on the caspase or trypsin activities (*SI Appendix*, Fig. S5). The chymotrypsin-like activity of human proteasome was moderately inhibited by **8** (mean IC_50_: purified 26S = 13 ± 0.88 µM; enriched THP-1 extracts IC_50_ = 40 ± 12.4 µM); however, a significant selectivity window of >100-fold was maintained.

Of particular note is the significant correlation between antileishmanial activity and the inhibition of proteasome chymotrypsin activity for a diverse set of compounds belonging to the same chemical series as **8** (Fig. 4*B*). This represents additional evidence of the proteasome as the predominant target of this compound series and that inhibition of proteasome activity accounts for the antiparasitic activity of **8** and its analogs. As expected for a proteasome inhibitor, **8** induces the accumulation of ubiquitylated proteins in the parasite cell in a dose-dependent manner (*SI Appendix*, Fig. S6).

The subunit specificity of **8** was assessed using the human active site probe Me4BodipyFL-Ahx3Leu3VS, commercially known as UbiQ-018 (Fig. 4*C*). This probe covalently labels all three catalytic subunits in human proteasomes (21). In contrast, only the β2 and β5 active sites of the parasite proteasome were fluorescently labeled by UbiQ-018 (*SI Appendix*, Table S8). In support of our enzymatic analyses, the presence of **8** selectively blocked UbiQ-018 labeling of the β5 subunit in *Leishmania* lysates while having no effect on binding to subunit β2. In another demonstration of compound **8** selectivity, incubation with this compound had no effect on UbiQ-018 labeling of the three catalytic subunits of the human proteasome. Furthermore, labeling of β5 in proteasomes prepared from our *Leishmania*-resistant cell lines could not be abolished by the presence of **8**. Classical proteasome inhibitors (e.g., bortezomib) blocked all subunits of the three parasites at the concentration tested.

Proteasome-enriched cell lysates were prepared from resistant cell line RES II, which maintains mutations encoding a G197C substitution in the β5 subunit as well as a T30A substitution in the β4 subunit. The chymotrypsin-like activity within this lysate was insensitive to inhibition by **8** up to concentrations of 100 µM (IC_50_ > 100 µM for RES II compared with IC_50_ = 0.16 ± 0.01 µM for WT *L. donovani* proteasomes). Lysates prepared from RES II were similarly insensitive to all of the compounds within the compound **8** series. Collectively, these data confirm that the proteasome is the primary target of **8**. Interestingly, bortezomib and carfilzomib, two anticancer drugs known to inhibit the chymotrypsin-like activity of human and *T. cruzi* proteasomes (20), inhibited this activity in lysates prepared from RES II and WT parasites with equal potency. These findings are consistent with the failure of RES I and RES III to demonstrate cross-resistance to bortezomib (*SI Appendix*, Fig. S7*A*) and suggest that **8** and its analogs may bind to a site that is distinct from bortezomib and carfilzomib binding sites. In contrast, RES I and RES II did demonstrate significant levels of cross-resistance to GNF6702, suggesting that these compounds do share a similar binding site (*SI Appendix*, Fig. S7*B*).

### Morphological Changes and Cell Cycle Defects.

Profound morphological changes were evident in parasites treated with compounds from this series. To gain additional insight into these compound-induced changes, *L. donovani* promastigotes were treated with **7** at a concentration equivalent to two times the established EC_50_ value for 6 h before analysis by transmission EM. Incubation with **7** caused swelling of promastigotes compared with untreated controls. In addition, there was a significant accumulation of intracellular vesicles within treated parasites (*SI Appendix*, Fig. S8). Based on our target identification studies, we reasoned that an inability to recycle key proteins via the proteasome left these parasites overcome by their own waste protein products. This accumulation of vesicles and associated morphological changes may also be explained by the activation of autophagy, an established consequence of proteasome inhibition (22).

Previous studies have demonstrated that specific inhibition of the proteasome can lead to cell cycle arrest, with treated cells accumulating at the G2/M checkpoint due to an inability to degrade G2/M-phase control cyclins (23, 24). This prompted us to analyze the effects of **7** on cell cycle progression in *L. donovani* and *T. brucei* (*SI Appendix*, Fig. S9). In both cases, treatment with **7** resulted in an accumulation of cells in G2/M and a decrease in the proportion of cells in G1 and S phases. These findings are entirely consistent with the compounds from this series inhibiting the proteasome of kinetoplastid parasites.

### Cryo-EM Structure of Compound 8 Bound to the *Leishmania tarentolae* 20S Proteasome.

**Fig. 5.**
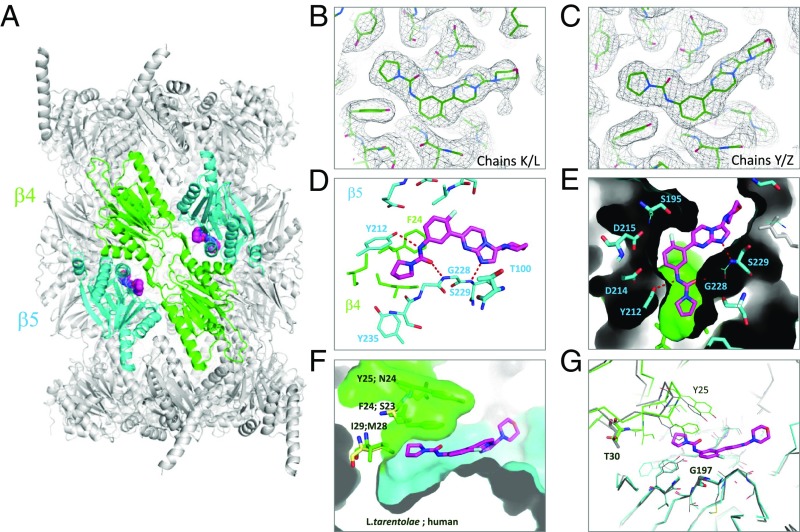
Experimental cryo-EM structure of **8** bound to the *L. tarentolae* 20S proteasome. (*A*) Two molecules of **8** (space fill) are found in 20S structure (protein ribbon) bound at the β4/β5 interface. Protein subunits β4 and β5 of this bound structure are shown in green and blue, and **8** is shown in magenta. (*B* and *C*) Electron map of the 2.8-Å cryo-EM structure of *L. tarentolae* 20S centered on **8** contoured at 3σ (black mesh); modeled ligand and protein shown as green lines. (*D*) A close-up view of the ligand binding site is shown; β4 residues are in green, are β5 residue labels are in blue. Polar interactions are indicated with red dashed lines. (*E*) Protein surface representation to highlight the complementarity of the pocket filled by **8**. (*F*) Structural basis of human selectivity. Key residue differences are labeled in black. (*G*) Overlay with apo structure is shown in gray. The positions of the two key residues (T30 and G197) where mutations have been observed in *L. donovani* are shown.

The potency of **8** against the nonpathogenic *Leishmania* spp. *Leishmania tarentolae* (EC_50_ value of 40 nM) was comparable with that achieved against *L. donovani* promastigotes (EC_50_ value of 14 nM). In addition, inhibition of the chymotrypsin-like activities catalyzed by *L. donovani* and *L. tarentolae* proteasomes by a set of compound **8** analogs correlated extremely well (*SI Appendix*, Fig. S10). These data suggest that inhibitor pharmacology against *L. tarentolae* is broadly similar to that seen for *L. donovani*, providing confidence in the use of this nonpathogenic surrogate for subsequent structural studies (*SI Appendix*, Table S9). Apo (3.3 Å) and **8**-liganded (2.8 Å) (Fig. 5*A*) *L. tarentolae* 20S proteasome structures were obtained using single-particle cryo-EM (*SI Appendix*, Figs. S11–S15 and Table S9).

The binding site and binding mode of **8** could be clearly determined by the excellent quality of the map (Fig. 5 *B* and *C* and *SI Appendix*, Fig. S16). Unexpectedly, the inhibitor does not bind in the proposed binding site of GNF6702 (20). The ligand binds mainly within the β5 subunits proximal to the catalytic T100 residue (Fig. 5*D*). Hydrogen bonds to the side chain of Y212 (β5) and to the backbone amides of G228 (β5) and S229 (β5) within the β5 subdomain all anchor the molecule (Fig. 5*D*) into a largely open site that shows great surface complementarity between the ligand and protein (Fig. 5*E*). The fluorine atom establishes an orthogonal multipolar interaction with the backbone carbonyl carbon of S195 and a weak hydrogen bond with the side chain of the same residue. The pyrrolidine carboxamide sits in the only enclosed part of the site in a narrow hydrophobic pocket that is limited in depth by I29 (β4) and capped by the pi-stacked residues of F24 (β4) and Y25 (β4) (Fig. 5*F*). These three amino acids are completely conserved among *L. donovani*, *T. cruzi*, and *T. brucei* but become M28 (β4), S23 (β4), and N24 (β4) within the human protein (Fig. 5*F* and *SI Appendix*, Fig. S18). Interestingly, mutation of these same residues confers resistance to GNF6702 in *T. cruzi* (20), suggesting that the two series may have overlapping binding sites. In comparing the binding mode of **8** with the binding mode of bortezomib in the human structure (25), it is possible to observe that the two molecules partially overlap (*SI Appendix*, Fig. S19).

### Molecular Modeling.

To investigate the selectivity of **8** against the human 20S proteasome and rationalize the role of the single-point mutations causing resistance, a homology model of the *L. donovani* 20S proteasome β4 and β5 subunits was generated using the *L. tarentolae* 20S proteasome cryo-EM structure as a template. The *L. tarentolae* 20S proteasome β4 and β5 subunits are highly homologous to the *L. donovani* β4 and β5 subunits (overall sequence identities of 95 and 98%, respectively), and in particular, the 26 amino acids forming the binding site that recognizes **8** are entirely conserved, giving the opportunity to build a very robust model (*SI Appendix*, Figs. S20 and S21*A*)

### Molecular Basis for Selectivity Against the Human 20S Proteasome.

Despite several differences in the primary sequence, this binding site can be found in *L. donovani*, *L. tarentolae*, and human 20S proteasomes (*SI Appendix*, Fig. S21*B*). However, of the nine amino acids that are different in the human structure compared with *L. donovani*, the majority are located where the pyrrolidine ring binds. In *L. tarentolae*, the pyrrolidine ring binds in a hydrophobic pocket formed by A23, F24, Y25, I27, and I29 side chains and K28 backbone from the β4 subunit and Y212, V227, and Y235 side chains and S231 backbone from the β5 subunit. In the human proteasome, most of the β4 residues defining this pocket are different (*SI Appendix*, Fig. S20), leading to a considerable change in morphology. In the human structure, the pyrrolidine binding cavity is more open, shallower, and solvent exposed, thus losing its hydrophobic character (*SI Appendix*, Fig. S22). Thus, the human structure is not capable of establishing the important hydrophobic and π-stacking interactions with the inhibitor (F24 in *L. tarentolae* corresponds to S23 in human). These changes also impact the water molecule network in the binding site. A set of three highly unstable water molecules can be identified in the hydrophobic cavity in *L. tarentolae*, which can be easily replaced by the ligand on binding. In contrast, due to the polar nature of the side chains (S23, N24) and the exposure to the bulk of the solvent, the water network in the human structure is more stable and less easily displaced by the ligand (*SI Appendix*, Fig. S23).

Differences in ligand–protein electrostatic complementarity between the *L. tarentolae* and human proteasomes can also provide additional insights into the observed selectivity. A combination of inductive and mesomeric effects in **8** results in a nonhomogeneous charge distribution (*SI Appendix*, Fig. S24 *A* and *B*). In the *L. tarentolae* structure, the hydrogens on carbon 3 and 4 of the fluorophenyl system are characterized by an accumulation of positive charge that sits in a negatively charged area of the proteasome defined by D214 and D215 (*SI Appendix*, Fig. S24 *C* and *D*). In the human structure, one of the negatively charged residues is lost as D215 replaces S215, decreasing the negative character of the binding site and impacting the strength of the dipole–charge interaction with the ligand. Other residues within the β5 subunit are generally conserved or do not impact ligand binding. In the morpholine binding region of *L. tarentolae*, A268 changes to tyrosine but does not clash with the ligand.

### Drug Resistance Mutations.

The structural basis of the resistance mutations identified in our studies [G197S (β5), G197C (β5), and T30A (β4)] can be rationalized based on the distinct inhibitor site that we have uncovered. The alpha carbon of G197 forms part of the floor of the ligand binding pocket just below the fluorophenyl ring (Fig. 5*G*). The presence of the larger hydrophilic side chain in the serine and cysteine mutants would result in a steric clash with this fused aromatic system and would be detrimental to inhibitor activity. In contrast, T30A (β4) has no direct interactions with the ligand. It lies at a pivot point between the β4 and β5 subunits, allowing the β loop, which contains I29 (β4), F24 (β4), and Y25 (β4), to wedge open to create the pyrrolidine pocket. Comparison of the apo and liganded structures obtained by cryo-EM suggests that a T30A mutation would alter the ease of this motion, changing the access to this slim pocket, which is important for activity and selectivity (Fig. 5*G*). In addition, T30 establishes a hydrogen bond with Q222 (β5). These interactions seem to provide an anchor point to the β-sheet in β5 (L224–V227) that defines the binding site for **8**. A T30A mutation would remove the anchor point, introducing instability to the binding site.

## Conclusion

Here, we describe the development of a preclinical candidate for VL. This compound acts through inhibition of the chymotrypsin-like activity catalyzed by the β5 subunit of the *L. donovani* proteasome, demonstrating good selectivity over the human enzyme. The compound selectively inhibits the parasite enzyme. High-resolution cryo-EM structures of apo and **8**-bound *L. tarentolae* 20S proteasome have revealed a previously undiscovered binding site for inhibitors of the chymotrypsin-like activity. This site lies between the β4 and β5 subunits and exploits an induced cavity that is lined on one side by β4 residues that are divergent between human and kinetoplastid protozoan. While this binding pocket differs from the cavity suggested by Khare et al. (20) in *T. cruzi*, which accommodates GNF6702, it is consistent with all of our experimental observations and mutational data. Compound **8** is currently undergoing preclinical development, and it is advancing toward human clinical trials. The data generated to date provide every reason to believe that **8** can become a much-needed safe oral treatment for patients suffering from this devastating neglected tropical disease.

## Materials and Methods

All regulated procedures, at the University of Dundee, on living animals was carried out under the Animals (Scientific Procedures) Act 1986, as amended in 2012 (and in compliance with European Union Directive EU/2010/63). All animal studies were reviewed by GlaxoSmithKline’s (GSK) internal ethical review committee and performed in accordance with Animals (Scientific Procedures) Act 1986 and the GSK Policy on the Care, Welfare, and Treatment of Laboratory Animals (UK 1986). Usage of human-sourced macrophages was approved by the Scottish National Blood Transfusion Service committee for the governance of blood and tissue samples for nontherapeutic use, and donor research, and was in accord with the terms of the informed consents. Full details are in *SI Appendix*. This includes the following information: (*i*) the chemical synthesis of compounds described in the paper, (*ii*) the methods for in vitro parasite assays, (*iii*) the methods for in vitro and in vivo drug metabolism and pharmacokinetics, (*iv*) the methods for efficacy studies, (*v*) the methods for preclinical safety studies, (*vi*) the methods for mode of action studies, (*vii*) details of cryo-EM studies, (*viii*) details of molecular modeling studies, and (*ix*) ethical statements.

## Supplementary Material

Supplementary File

## References

[1] Burza S, Croft SL, Boelaert M (2018). Leishmaniasis. Lancet.

[2] Control of Neglected Tropical Diseases (NTD) World Health Organization (2015). http://www.who.int/leishmaniasis/burden/Status_of_endemicity_of_VL_worldwide_2015_with_imported_cases.pdf?ua=1.

[3] Ready PD (2014). Epidemiology of visceral leishmaniasis. Clin Epidemiol.

[4] Lindoso JAL, Moreira CHV, Cunha MA, Queiroz IT (2018). Visceral leishmaniasis and HIV coinfection: Current perspectives. HIV AIDS (Auckl).

[5] Rijal S (2013). Increasing failure of miltefosine in the treatment of Kala-azar in Nepal and the potential role of parasite drug resistance, reinfection, or noncompliance. Clin Infect Dis.

[6] Dorlo TP (2014). Failure of miltefosine in visceral leishmaniasis is associated with low drug exposure. J Infect Dis.

[7] Khalil EA (2014). Safety and efficacy of single dose versus multiple doses of AmBisome for treatment of visceral leishmaniasis in eastern Africa: A randomised trial. PLoS Negl Trop Dis.

[8] Wasunna M (2016). Efficacy and safety of AmBisome in combination with sodium stibogluconate or miltefosine and miltefosine monotherapy for African visceral leishmaniasis: Phase II randomized trial. PLoS Negl Trop Dis.

[9] Alvar J, Arana B, Rivas L, Gil C (2018). Leishmaniasis, impact and therapeutic needs. Drug Discovery for Leishmaniasis.

[10] Gilbert IH (2013). Drug discovery for neglected diseases: Molecular target-based and phenotypic approaches. J Med Chem.

[11] Don R, Ioset JR (2014). Screening strategies to identify new chemical diversity for drug development to treat kinetoplastid infections. Parasitology.

[12] Martín J, Cantizani J, Peña I, Rivas L, Gil C (2018). The pursuit of novel anti-leishmanial agents by high-throughput screening (HTS) of chemical libraries. Drug Discovery for Leishmaniasis.

[13] De Rycker M (2013). Comparison of a high-throughput high-content intracellular Leishmania donovani assay with an axenic amastigote assay. Antimicrob Agents Chemother.

[14] Böhm H-J, Flohr A, Stahl M (2004). Scaffold hopping. Drug Discov Today Technol.

[15] Drugs for Neglected Diseases initiative, DNDi (2018). https://www.dndi.org/diseases-projects/leishmaniasis/tpp-vl/.

[16] Nühs A (2015). Development and validation of a novel *Leishmania donovani* screening cascade for high-throughput screening using a novel axenic assay with high predictivity of Leishmanicidal intracellular activity. PLoS Negl Trop Dis.

[17] Wyllie S (2018). Cyclin-dependent kinase 12 is a drug target for visceral leishmaniasis. Nature.

[18] Escobar P, Yardley V, Croft SL (2001). Activities of hexadecylphosphocholine (miltefosine), AmBisome, and sodium stibogluconate (Pentostam) against *Leishmania donovani* in immunodeficient scid mice. Antimicrob Agents Chemother.

[19] Alsford S (2012). High-throughput decoding of antitrypanosomal drug efficacy and resistance. Nature.

[20] Khare S (2016). Proteasome inhibition for treatment of leishmaniasis, Chagas disease and sleeping sickness. Nature.

[21] Berkers CR (2012). Probing the specificity and activity profiles of the proteasome inhibitors bortezomib and delanzomib. Mol Pharm.

[22] Zhu K, Dunner K, McConkey DJ (2010). Proteasome inhibitors activate autophagy as a cytoprotective response in human prostate cancer cells. Oncogene.

[23] Sun GJ (2004). Mechanism of G2/M cell cycle arrest before apoptosis in leukemia cell line HL-60 induced by proteasome inhibitor MG132. Chin J Cancer.

[24] Ling YH (2003). Mechanisms of proteasome inhibitor PS-341-induced G(2)-M-phase arrest and apoptosis in human non-small cell lung cancer cell lines. Clin Cancer Res.

[25] Schrader J (2016). The inhibition mechanism of human 20S proteasomes enables next-generation inhibitor design. Science.

