# Supplementary material for: Preclinical candidate for the treatment of visceral leishmaniasis that acts through proteasome inhibition
Source: Proc Natl Acad Sci U S A. 2019 Apr 8;116(19):9318–23. doi: 10.1073/pnas.1820175116 (PMC6511062; doi:10.1073/pnas.1820175116)
Supplement: Supplementary File [file pnas.1820175116.sapp.pdf]

Supplementary Materials for A PRECLINICAL CANDIDATE FOR THE TREATMENT OF VISCERAL LEISHMANIASIS THAT ACTS THROUGH PROTEASOME INHIBITION

Authors:

Susan Wyllie[a]/ Stephen Brand[a], Michael Thomas[a], Manu De Rycker[a], Chun-wa Chung[d], Imanol Pena[b], Ryan P. Bingham[d], Juan A. Bueren-Calabuig[a], Juan Cantizani[b], David Cebrian[b], Peter D. Craggs[d], Liam Ferguson[a], Panchali Goswami[d], Judith Hobrath[a], Jonathan Howe[c], Laura Jeacock[a], Eun-Jung Ko[a], Justyna Korczynska[d], Lorna MacLean[a], Sujatha Manthri[a], Maria S. Martinez[b], Lydia Mata-Cantero[b], Sonia Moniz[a], Andrea Nuhs[a], Maria Osuna-Cabello[a], Erika Pinto[a], Jennifer Riley[a], Sharon Robinson[c], Paul Rowland[d], Frederick R. C. Simeons[a], Yoko Shishikura[a], Daniel Spinks[a], Laste Stojanovski[a], John Thomas[a], Stephen Thompson[a], Elisabet Viayna Gaza[a], Richard J. Wall[a], Fabio Zuccotto[a], David Horn[a], Michael A. J. Ferguson[a], Alan H. Fairlamb[a], Jose M. Fiandor[b], Julio Martin[b], David W. Gray[a], Timothy J. Miles[b], Ian H. Gilbert[a], Kevin D. Read[a,1], Maria Marco[b,1], Paul G. Wyatt[a,1].

[a] Drug Discovery Unit, Wellcome Centre for Anti-Infectives Research, Division of Biological Chemistry and Drug Discovery, University of Dundee, Dundee, DD1 5EH, United Kingdom.

[b] Global Health R&D, GlaxoSmithKline, Tres Cantos, Spain.

[c] David Jack Centre for R&D, GlaxoSmithKline, Ware, United Kingdom.

[d] Medicines Research Centre, Gunnels Wood Road, Stevenage, Hertfordshire, SG1 2NY, United Kingdom.

[1]To whom correspondence should be addressed. Email:

Kevin D. Read: K.Read@dundee.ac.uk

Maria Marco: maria.m.marco@gsk.com

Paul G. Wyatt: P.G.Wyatt@dundee.ac.uk

This PDF file includes:

Materials and Methods 3

1. Synthetic methods 3
   - a. Chemical synthesis of compound **1** 3
   - b. Chemical synthesis of compound **2** 3
   - c. Chemical synthesis of compound **3:** 3
   - d. Chemical synthesis of compound **4:** 5
   - e. Chemical synthesis of compound **5:** 5

www.pnas.org/cgi/doi/10.1073/pnas.20175116

f. Chemical synthesis of compound **6a:** 6
g. Chemical synthesis of compound **6:** 7
h. Chemical synthesis of compound **7a:** 8
i. Chemical synthesis of compound **7**: 9
j. Chemical synthesis of compound **8**: 10
2. CAD solubility assay 12
3. Solubility of Solid Compounds in Fasted Simulated Intestinal Fluid (FaSSIF). ... 12
4. Parasite assays 13
a. Rate of Kill 13
6. Pharmacokinetic studies. 13
7. *In vivo* efficacy in female Balb/C mice 14
8. Preclinical safety studies 15
a. Genotoxicity 15
b. 7 day rat toxicology studies 16
9. Mode of action studies 17
a. Cell lines and culture conditions 17
b. RITseq library screening 17
c. Generation of drug-resistant parasites 17
d. DNA sequencing 18
e. Generation of overexpression constructs and LdBOB transgenic cell lines. 18
f. *T. brucei* RNAi constructs and cell lines 18
h. Morphological analysis 19
i. FACS analysis 19
j. Proteasome enrichment by ultracentrifugation 19
k. Proteasome activity assays 20
m. Activity-Based Probe Labelling of THP1 and *L. donovani* proteasome 20
10. Measuring Proteolytic activity of *Leishmania tarentolae* Proteasome 21
11. Cryo-EM of 20S *Leishmania tarentolae* proteasome 21
a. Purification of 20S *Leishmania tarentolae* proteasome. 21
12. Cryo-grid preparation and data collection 22
a. Purified proteasome was used to prepare Cryo-grids with and without compound **8**. 22
13. Proteasome Cryo-EM structure refinements 23
a. Apo and ligand structures 23
14. Molecular modelling 23
Supplementary figures 25
Supplementary Tables 46
Ethical Statements .... **Error! Bookmark not defined.**

Materials and Methods

1. Synthetic methods

Chemicals and solvent were purchased from the Aldrich Chemical Co., Fluka, VWR, Acros, Fisher Chemicals, Fluorochem, Apollo Scientific and Alfa Aesar. All chemical were used as received unless otherwise stated. Air- and moisture- sensitive reactions were carried out under an inert atmosphere of argon in oven-dried glassware. Analytical thin-layer chromatography (TLC) was performed on pre-coated TLC plates (layer 0.20 mm silica gel 60 with fluorescent indicator UV254, from Merck). Developed plates were air-dried and analyzed under a UV lamp (UV254/365 nm). Flash column chromatography was performed using prepacked silica gel cartridges (230- 400 mesh, 40- 63 µM, from SiliCycle) using a Teledyne ISCO Combiflash Companion or Combifalsh Retrieve. ^{1}H NMR spectra were recorded on a Bruker Avance DPX 500 spectrometer (at 500.1 MHz). Chemical shifts (δ) are expressed in ppm recorded using the residual solvent as the internal reference in all cases. Signal splitting patterns are described as singlet (s), doublet (d), triplet (t), quartet (q), multiplet (m), broad (b), or a combination thereof. Coupling constants (*J*) are quoted to the nearest 0.1 Hz. LC-MS analyses were performed with either an Agilent HPLC 1100 series connected to a Bruker Daltonics MicrOTOF or an Agilent Technologies 1200 series HPLC connected to an Agilent Technologies 6130 quadrupole LC/MS, where both instruments were connected to an Agilent diode array detector. All assay compounds had a measure purity of ≥95% as determined using this analytical LC-MS system (TIC and UV).

a. Chemical synthesis of compound **1**

Commercially available**.**

b. Chemical synthesis of compound **2**

Followed described procedure[1].

c. Chemical synthesis of compound **3:**

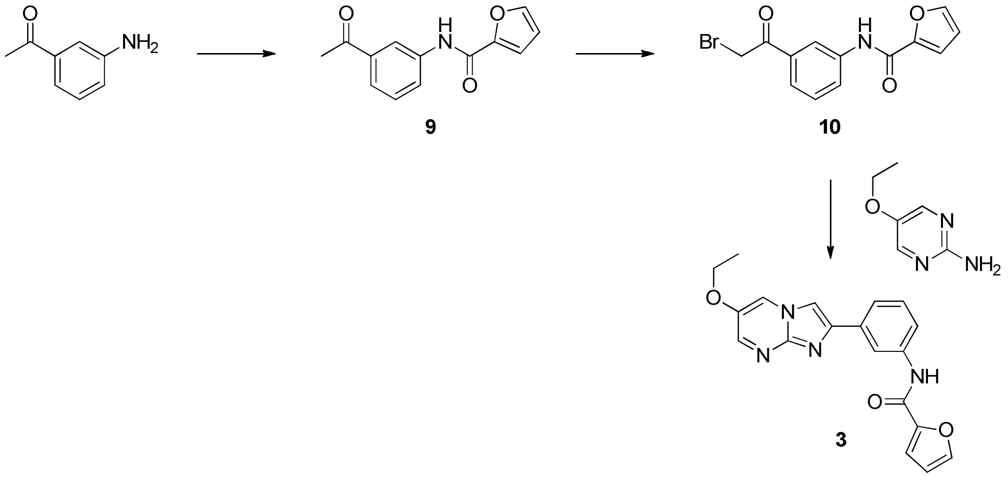

Scheme S1. Synthesis of compound **3**.

*Synthesis of N-(3-acetylphenyl)furan-2-carboxamide (**9**)*

To a mixture of 1-(3-aminophenyl)ethanone (4 g, 29.6 mmol) in pyridine (80 mL) was added furan-2-carbonyl chloride (3.86g, 29.6mmol) dropwise. The mixture was heated in a microwave (160^0 C, 11 min). After cooling, the mixture was diluted with DCM (250 mL) and washed with sat. aq. $NaHCO_3$ (2 x 350 mL) and water (2 x 350 mL). The organics were dried (phase separator), concentrated and the residue chromatographed (hexane/ EtOAc 0 to 100% gradient). Fractions corresponding to product were combined, concentrated and the residue triturated with Et_2O and dried to give **9** as a white solid (5.73 g, 24.7 mmol, 84 %). ^{1}H NMR (500 MHz, $CDCl_3$): δ 8.23 (s, 1H), 8.20 – 8.18 (m, 1H), 8.03 – 8.00 (m, 1H), 7.77 – 7.74 (m, 1H), 7.57 – 7.55 (m, 1H), 7.52 – 7.48 (m, 1H), 7.30 – 7.28 (m, 1H), 6.62 – 6.60 (m, 1H), 2.65 (s, 3H); m/z = 230.0 $[M+H]^+$.

Synthesis of N-(3-(2-bromoacetyl)phenyl)furan-2-carboxamide (**10**).

To a solution of *N*-(3-acetylphenyl)furan-2-carboxamide (**9**, 0.15 g, 0.65 mmol) in THF (3 mL) was added trimethylphenylammonium tribromide (0.246 g, 0.65 mmol) portion-wise over 10 min and stirred at RT overnight. The mixture was filtered, and the filtrate concentrated and chromatographed (hexane / EtOAc 0 to 100%) to yield **10** which was 77% pure by HPLC (contaminated with starting material and dibrominated compound). This material was used without further purification (0.118 g, 0.29 mmol, 44%); m/z = 310.0, 312.0 $[M+H]^+$.

Synthesis of N-(3-(6-ethoxyimidazo[1,2-a]pyrimidin-2-yl)phenyl)furan-2-carboxamide (**3**).

A mixture of *N*-[3-(2-bromoacetyl)phenyl]furan-2-carboxamide (**10**, 0.2 g, 0.65 mmol) and 2-amino-5-ethoxypyrimidine (0.1 g, 0.72 mmol) in DMF (2 mL) was stirred at 90 ^{0}C overnight. The mixture was cooled to room temperature, diluted with 2N NaOH (10 mL) and extracted with EtOAc (2 x 15 mL). The organic extracts were combined, dried over $MgSO_4$, filtered, concentrated and the residue chromatographed (hexane/EtOAc 0 to 100% gradient). Fractions corresponding to product were combined and concentrated and the residue triturated with EtOAc / MeOH (1:9) and dried to give **3** (0.035 g, 0.09 mmol, 15%). ^{1}H NMR (400 MHz, DMSO-d_6): δ 10.29 (s, 1H), 8.70 (d, J = 2.9 Hz, 1H), 8.43 – 8.38 (m, 2H), 8.21 (s, 1H), 7.97 (d, J = 1.0 Hz, 1H), 7.81 – 7.77 (m, 1H), 7.69 (d, J = 7.7 Hz, 1H), 7.45 – 7.40 (m, 2H), 6.73 (dd, J = 3.5 and 1.7 Hz, 1H), 4.10 (q, J = 6.9 Hz, 2H), 1.41 (t, J = 7.0 Hz, 3H); ^{13}C NMR (500 MHz, DMSO-d_6): δ 156.3, 147.5, 145.9, 145.5, 145.1, 144.9, 144.1, 139.1, 134.1, 129.1, 120.9. 120.0, 117.5, 116.8, 114.8, 112.2, 108.1, 65.0, 14.4; m/z $[M+H]^+$ calculated for $C_{19}H_{17}FN_4O_3$, 349.1295; found, 349.1308.

d. Chemical synthesis of compound **4:**

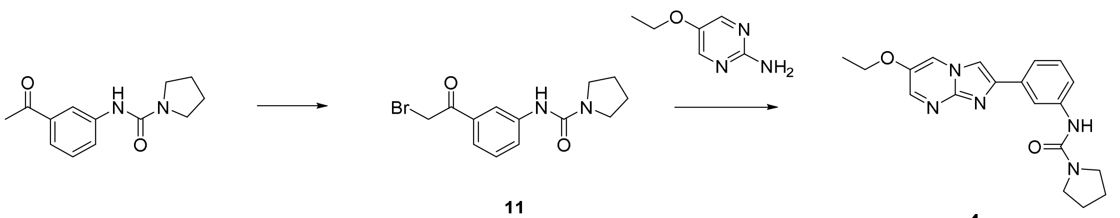

11 4

Scheme S2. Synthesis of compound **4**.

Synthesis of N-(3-(2-bromoacetyl)phenyl)pyrrolidine-1-carboxamide (**11**).

To a solution of *N*-(3-acetylphenyl)pyrrolidine-1-carboxamide (0.51 g, 2.2 mmol) in THF (5 mL) was added trimethylphenylammonium tribromide (0.826 g, 2.2 mmol) and stirred at RT overnight. The reaction mixture was filtered, and the filtrate concentrated and chromatographed (hexane / EtOAc 0 to 100% gradient). Fractions were combined and dried to give **11** (contaminated with starting material and dibrominated compound), which was used in the next step without further purification (0.53 g, 0.89 mmol 40%). m/z = 313.1 $[M+H]^+$.

Synthesis of N-[3-(6-ethoxyimidazo[1,2-a]pyrimidin-2-yl)phenyl]pyrrolidine-1-carboxamide (**4**).

A mixture of *N*-[3-(2-bromoacetyl)-4-fluoro-phenyl]pyrrolidine-1-carboxamide (**11**, 0.0863 g, 0.28 mmol) and 2-amino-5-ethoxypyrimidine (0.0447 g, 0.32 mmol) in DMF (1.5 mL) was stirred at 90 ^{0}C overnight, cooled to room temperature, filtered and purified by mass-directed auto-prep (C18 column, H_2O-0.1%NH_3/MeCN 20 to 95%). Fractions corresponding to product were combined and chromatographed (EtOAc) to give **4** (0.012 g, 0.04 mmol, 11%). ^{1}H NMR (400 MHz, DMSO-d_6): δ 8.68 (s, 1H), 8.40 (s, 1H), 8.24 (s, 1H), 8.15 (s, 2H), 7.57 (d, *J* = 8.3 Hz, 1H), 7.50 (d, *J* = 7.3 Hz, 1H), 7.29 (dd, *J* = 7.7, 7.7 Hz, 1H), 4.10 (q, *J* = 6.8 Hz, 2H), 3.40 (s, 4H), 1.87 (s, 4H), 1.40 (t, *J* = 6.8 Hz, 3H); ^{13}C NMR (500 MHz, DMSO-d_6): δ 153.9, 145.6, 145.5, 144.7, 144.0, 141.2, 133.7, 128.7, 119.0, 118.7, 116.8, 116.6, 107.9, 64.9, 45.7, 25.1, 14.4; m/z $[M+H]^+$ calculated for $C_{19}H_{22}N_5O_2$, 352.1768; found, 352.1782.

e. Chemical synthesis of compound **5:**

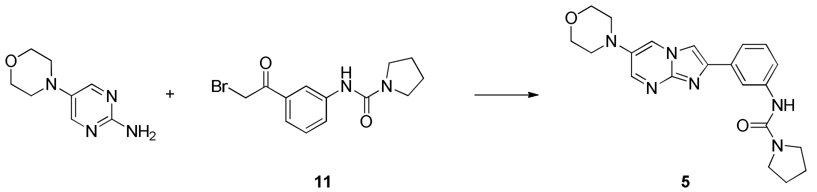

11 5

Scheme S3. Synthesis of compound **5**.

Synthesis of N-[3-(6-morpholinoimidazo[1,2-*a*]pyrimidin-2-yl)phenyl]pyrrolidine-1-carboxamide (**5**).

A mixture of *N*-(3-(2-bromoacetyl)phenyl)pyrrolidine-1-carboxamide (**11**, 0.086 g, 0.28 mmol) and 2-amino-5-morpholinopyrimidine[2] (0.05 g, 0.28 mmol) in DMF (1.5 mL) was

stirred at 90 ^{0}C overnight and cooled to room temperature. The crude mixture was diluted with 2N NaOH (10 mL) and extracted with EtOAc (2 x 15 mL). The organic extracts were combined, dried over $MgSO_4$, filtered and concentrated. The residue was chromatographed (EtOAc / MeOH 0 to 20%). Fractions corresponding to product were combined and further purified through mass-directed auto-prep (C18 column, H_2O-0.1%NH_3/MeCN 5 to 95%) to give **5** (0.0025 g, 0.006 mmol, 2%). ^{1}H NMR (400 MHz, $CDCl_3$): δ 8.46 (s, 1H), 7.84 (s, 1H), 7.77 (s, 2H), 7.69 (d, *J* = 8.2 Hz, 1H), 7.46 (d, *J* = 7.7 Hz, 1H), 7.36 – 7.30 (m, 1H), 3.93 – 3.88 (m, 4H), 3.52 – 3.47 (m, 4H), 3.13 – 3.09 (m, 4H), 2.46 – 2.42 (m, 4H); m/z = 393.2 $[M+H]^+$.

f. Chemical synthesis of compound **6a:**

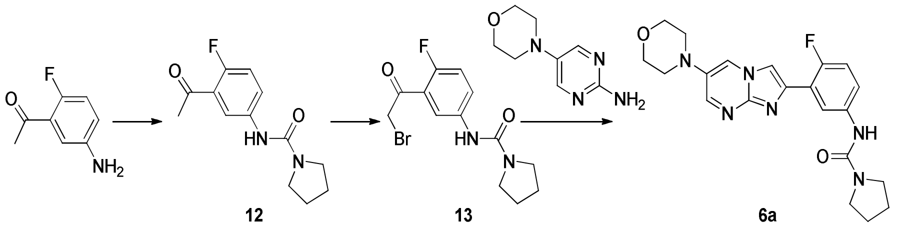

Scheme S4. Synthesis of compound **6a**.

Synthesis of N-(3-acetyl-4-fluorophenyl)pyrrolidine-1-carboxamide (**12**).

To a stirred solution of 1-(5-amino-2-fluoro-phenyl)ethanone (10.0 g, 65.3 mmol) and 4-dimethylaminopyridine (400 mg, 3.3 mmol) in pyridine (100 mL) / DCM (400 mL) at RT was added pyrrolidine-1-carbonyl chloride (13.08 g, 97.9 mmol) dropwise. The reaction was stirred at 50 ^{0}C for 72 h, concentrated, and the residue diluted with DCM (400 mL), washed with brine (200 mL) and the organic phase dried ($MgSO_4$) and concentrated. To the resulting powder was added a mixture of EtOAc:Et_2O(1:1), stirred for 12 h and the solid collected by filtration and dried to give **12** (13.55 g, 53.6 mmol, 82%). ^{1}H NMR (500 MHz, DMSO-d_6): δ 8.37 (s, 1H), 7.94 (dd, *J* = 6.7 and 2.8 Hz, 1H), 7.85 – 7.81 (m, 1H), 7.23 (dd, *J* = 10.9 and 9.0 Hz, 1H), 3.38 – 3.34 (m, 4H), 2.56 (d, *J* = 4.6 Hz, 3H), 1.88 –1.83 (m, 4H); m/z = 251.1 $[M+H]^+$.

Synthesis of N-(3-(2-bromoacetyl)-4-fluorophenyl)pyrrolidine-1-carboxamide (**13**).

N-(3-acetyl-4-fluoro-phenyl)pyrrolidine-1-carboxamide (**12**, 11.7 g, 46.8 mmol) was dissolved in THF (400 mL), cooled to 0 ^{0}C and treated portion-wise with trimethyl(phenyl)ammonium tribromide (17.58 g, 46.8 mmol). The reaction was allowed to warm to RT, stirred overnight and concentrated. The residual solid was dissolved in DCM (500 mL), washed with water (2 x 200 mL), brine (200 mL) and sat. aq. $NaHCO_3$ (200 mL), dried over $MgSO_4$, filtered and concentrated. The residual solid was triturated with EtOAc/Et_2O (1:1) then EtOAc (200 mL), collected by filtration and dried to give crude **13** (contaminated with starting material and dibrominated compound) which was used without purification (12.47 g, 34.1 mmol, 73%). m/z = 329.0 $[M+H]^+$.

Synthesis of N-[4-fluoro-3-(6-morpholinoimidazo[1,2-a]pyrimidin-2-yl)phenyl]pyrrolidine-1-carboxamide (**6a**).

A mixture of *N*-(3-(2-bromoacetyl)-4-fluorophenyl)pyrrolidine-1-carboxamide (**13**, 3 g, 9.11 mmol) and 2-amino-5-morpholinopyrimidine (1.64 g, 9.11 mmol) in MeCN (60 mL)

was stirred at 60 ^{0}C for 48 h, cooled to RT, diluted with Et_2O (50 mL) and filtered. The resulting solid was suspended in a MeOH / EtOAc mixture (1:5, 300 mL), basified to pH = 14 with 2N NaOH, and stirred at RT for 1 h. The two layers were separated and the aqueous layer extracted with EtOAc (2 x 250 mL).The combined organic extracts were washed with brine (200 mL), dried over $MgSO_4$, filtered, concentrated and chromatographed (MeOH / EtOAc 0 to 10% gradient). Fractions corresponding to product were combined and concentrated and the resulting solid triturated with DCM and dried to give **6a** (1.19 g, 4.61 mmol, 51%). ^{1}H NMR (500 MHz, DMSO-d_6): δ 8.68 (d, J = 2.6 Hz, 1H), 8.48 (d, J = 2.4 Hz, 1H), 8.36 – 8.30 (m, 2H), 8.12 (d, J = 4.1 Hz, 1H), 7.66 – 7.62 (m, 1H), 7.20 – 7.15 (m, 1H), 3.82 – 3.78 (m, 4H), 3.43 – 3.37 (m, 4H), 3.13 – 3.09 (m, 4H), 1.90 – 1.85 (m, 4H); ^{13}C NMR (500 MHz, DMSO-d_6): δ 154.0, 153.8, 147.3. 144.9, 138.7, 137.4, 136.1, 120.7 (d, J = 13.3 Hz), 120.0 (d, J = 7.8 Hz), 119.0 (d, J = 3.1 Hz), 118.7, 115.3, (d, J = 22.9 Hz), 110.8 (d, J = 15.4 Hz), 65.8, 49.6, 45.7, 25.1; m/z [M+H]$^+$ calculated for $C_{21}H_{24}FN_6O_2$, 411.1939; found, 411.1941.

g. Chemical synthesis of compound **6:**

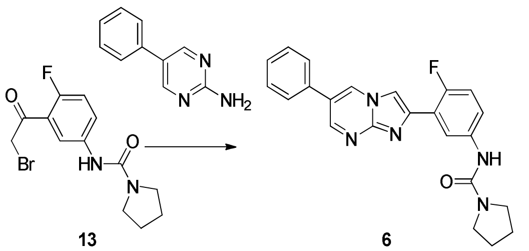

13 6

Scheme S5. Synthesis of compound **6**.

Synthesis of N-(4-fluoro-3-(6-phenylimidazo[1,2-a]pyrimidin-2-yl)phenyl)pyrrolidine-1-carboxamide (**6**).

A mixture of N-[3-(2-bromoacetyl)-4-fluoro-phenyl]pyrrolidine-1-carboxamide (**13**, 2.5 g, 6.46 mmol) and 2-amino-5-phenylpyrimidine (1.10 g, 6.46 mmol) in MeCN (50 mL) was stirred at 60 ^{0}C for 48 hours and monitored by LCMS. After the reaction was complete, it was cooled down to room temperature and filtered. The solid was suspended in a MeOH:EtOAc mixture (1:5, 300 mL) and basified to pH = 14 with 2N NaOH. The two layers were separated and the aqueous further extracted with EtOAc (2 x 250 mL) and the combined organic extracts were washed with brine (200 mL), dried over $MgSO_4$, filtered and concentrated to a yellow solid which was triturated with a mixture Et_2O:EtOAc (1:1) to give **6** (1.73 g, 4.26 mmol, 66%). ^{1}H NMR (500 MHz, DMSO): δ 9.35 (d, J = 2.6 Hz, 1H), 8.96 (d, J = 2.6 Hz, 1H), 8.44 (dd, J = 2.9, 6.9 Hz, 1H), 8.36 (s, 1H), 8.28 (d, J = 4.1 Hz, 1H), 7.79 (d, J = 7.2 Hz, 2H), 7.69 (ddd, J = 4.5, 2.9, 8.8 Hz, 1H), 7.57 (dd, J = 7.7, 7.7 Hz, 2H), 7.48 (t, J = 7.4 Hz, 1H), 7.22 (dd, J = 8.9, 11.1 Hz, 1H), 3.53- 3.48 (m, 4H), 2.04- 2.00 (m, 4H); m/z = 402.2 [M+H]$^+$.

h. Chemical synthesis of compound **7a:**

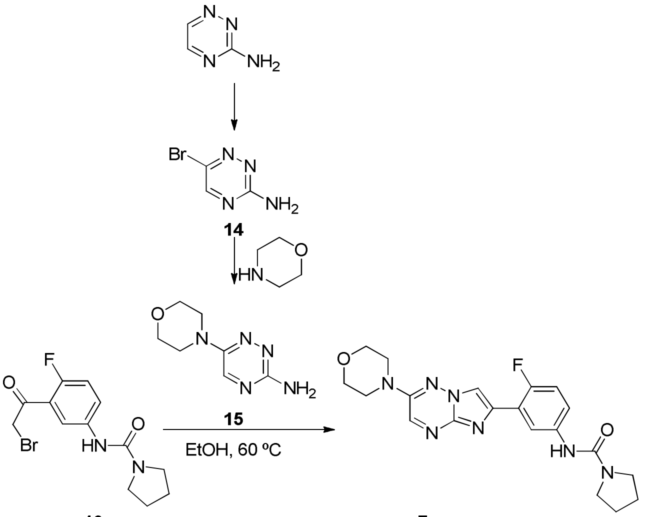

13 7a

Scheme S6. Synthesis of compound **7a**.

*Synthesis of 6-Bromo-1,2,4-triazin-3-amine (**14**)*

A solution of 1,2,4-triazin-3-amine (3.0 g, 31.2 mmol) in water (300 mL) was cooled to 0-5 ^{0}C. Bromine (4 mL, 78 mmol) was added drop-wise over 45 min, then the reaction mixture was stirred overnight at room temperature. A saturated aqueous Na_2SO_3 solution (100 mL) was added to the reaction mixture and then the mixture was basified to pH 12 by addition of 2N aqueous NaOH solution. The mixture was extracted with ethyl acetate (2 × 200 mL). The combined organic layers were dried over sodium sulfate, filtered and concentrated under reduced pressure to afford **14** as a pale yellow solid (2.51 g, 14.3 mmol, 46%). ^{1}H NMR (400 MHz, DMSO-d6): δ 8.40 (s, 1H), 7.47 (s br, 2H).

*Synthesis of 6-morpholino-1,2,4-triazin-3-amine (**15**).*

6-Bromo-1,2,4-triazin-3-amine (**14**, 4.5 g, 25.7 mmol) was loaded in equal portions into three 20 mL microwave vials fitted with stirrer bars, then morpholine (8.0 mL) added to each. The sealed vials were sonicated for 5 min at 60 ^{0}C to ensure a complete solution was obtained prior to heating to 120 ^{0}C for 1h after which time TLC (100% EtOAc) indicated complete consumption of starting material. The reactions were combined and concentrated *in vacuo* to a brown gum which was azeotroped with EtOAc (20 mL x 3), DCM (20 mL) then MeOH (20 mL) until a hard gum was obtained. This was then diluted with 10 mL of MeOH and the solution treated with 7N ammonia in MeOH (50 mL) stirred for 30 min and the solution concentrated to give a thick gum. Purification by chromatography (100% EtOAc to 15% 7M methanolic ammonia in EtOAc) gave **15** as an off-white powder (1.75 g, 9.66 mmol, 38%). ^{1}H NMR (500 MHz, DMSO-d6): δ 8.30 (s, 1H), 6.39 (bs, 2H), 3.75 – 3.71 (m, 4H), 3.33 – 3.30 (m, 4H).

*Synthesis of N-(4-fluoro-3-(2-morpholinoimidazo[1,2-b][1,2,4]triazin-6-yl)phenyl)pyrrolidine-1-carboxamide (**7a**).*

6-Morpholino-1,2,4-triazin-3-amine (**15**, 1.26 g, 6.9 mmol) in dry EtOH (80 mL) was warmed to 60 ^{0}C to give a solution which was treated portion-wise with stirring with N-[3-(2-bromoacetyl)-4-fluoro-phenyl]pyrrolidine-1-carboxamide (**13**, 2.30 g, 6.9 mmol). The reaction mixture was stirred at 60 ^{0}C for 72 h, allowed to cool to RT overnight, and the resulting precipitate collected by filtration onto a sintered funnel. The off-white solid was quickly washed with ice-cold EtOH and dried to give **7a** (1.28 g, 3.1 mmol, 44% yield) as a pale yellow crystalline solid.

The filtrate was concentrated, diluted with DCM (100 mL), washed with saturated aqueous sodium hydrogencarbonate (100 mL) and brine (100 mL), dried ($MgSO_4$) and concentrated. Chromatography (100% EtOAc moving to 10% 7M methanolic ammonia in EtOAc) and collection of clean fractions gave a solid which, after trituration from ice cold EtOH and collection by filtration, gave an additional 850 mg of **7a** (approx. 90% purity). This material was recrystallised from hot EtOH and both crops combined, re-dissolved in EtOH (100 mL) and concentrated slowly *in vacuo*. The precipitate was cooled with stirring and collected by filtration to give **7a** (1.91 g, 66% yield). ^{1}H NMR (500 MHz, DMSO-d6): δ 8.70 (s, 1H), 8.35 - 8.30 (m, 2H), 8.12 (d, J = 4.1 Hz, 1H), 7.62 (ddd, J = 8.9 Hz, 4.5 Hz, 2.9 Hz, 1H), 7.18 (dd, J = 10.9 Hz, 8.9 Hz, 1H), 3.77- 3.75 (m, 4H), 3.56- 3.54 (m, 4H), 3.40- 3.38 (m, 4H), 1.87- 1.85 (m, 4H); ^{13}C NMR (500 MHz, DMSO-d_6): δ 156.0, 154.5, 154.1, 150.9, 139.8, 138.4, 137.7 (d, J = 27.1 Hz), 120.9 (d, J = 13.2 Hz), 120.6 (d, J = 7.8 Hz), 119.1, 115.8 (d, J = 22.5 Hz), 114.1 (d, J = 14.5 Hz), 66.0, 46.1, 45.8, 25.5; HRMS (m/z): $[M+H]^+$ calculated for $C_{20}H_{23}N_7O_2F$, 412.1897; found 412.1876.

i. Chemical synthesis of compound **7**:

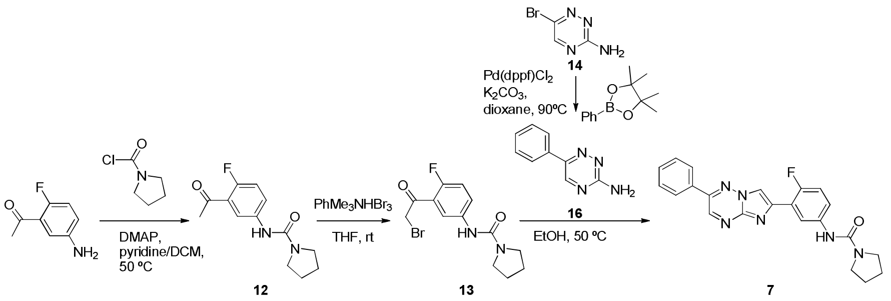

Scheme S7. Synthesis of compound **7**.

*Synthesis of 6-phenyl-1,2,4-triazin-3-amine (**16**).*

To a solution of 6-bromo-1,2,4-triazin-3-amine (**14**, 3.0 g, 17.1 mmol) and 4,4,5,5-tetramethyl-2-phenyl-1,3,2-dioxaborolane (5.25 g, 25.7 mmol) in 1,4-Dioxane (100 mL) was added a solution of potassium carbonate (7.1 g, 51.4 mmol) in water (20 mL). The resulting solution was deoxygenated by bubbling through a stream of nitrogen for 10 min, $Pd(dppf)Cl_2$.DCM (0.65 g, 0.8 mmol) was added, and the mixture stirred at 90 ^{0}C for 4h. The reaction mixture was cooled to RT, diluted with EtOAc (200 mL) and water (200 mL) and filtered to remove solid. The layers were separated, the aqueous layer extracted with EtOAc (3 x 50 mL), and the combined organics washed with brine, dried over $MgSO_4$, filtered and concentrated. The resulting slurry was triturated with 5:1 Et_2O / EtOAc and the solid collected by filtration, washed with ice-cold EtOAc and dried. The filtrate was cooled to 0 ^{0}C, and the resulting solid collected, dried and the two batches

combined to give **16** (2.06 g, 11.3 mmol, 66%). ^1H NMR (500 MHz, DMSO-d_6): δ 8.81 (s, 1H), 8.01 – 7.98 (m, 2H), 7.53 – 7.48 (m, 2H), 7.46 – 7.42 (1H, m), 7.34 (br s, 2H).

Synthesis of N-(4-fluoro-3-(2-phenylimidazo[1,2-b][1,2,4]triazin-6-yl)phenyl)pyrrolidine-1-carboxamide (**7**).

A slurry of 6-phenyl-1,2,4-triazin-3-amine (**16**, 0.777 g, 4.47 mmol) in EtOH (50 mL) was heated to 50 ^{0}C to form a solution which was treated portion-wise with N-(3-(2-bromoacetyl)-4-fluorophenyl)pyrrolidine-1-carboxamide (**13**, 1.44 g, 4.38 mmol). The reaction mixture was stirred at 60 ^{0}C for 3 days, cooled to room temperature and the resulting solid collected, washed with ice cold EtOH (10 mL) and dried under vacuum to give compound **7** (1.09 g, 2.68 mmol, 60%) as a yellow powder. ^1H NMR (500 MHz, DMSO-d_6): δ 9.25 (s, 1H), 8.60 (d, *J* = 4.0 Hz, 1H), 8.47 (dd, *J* = 6.8 Hz, 2.8 Hz, 1H), 8.40 (s, 1H), 8.18 – 8.15 (m, 2H), 7.71 (ddd, *J* = 8.9 Hz, 4.5 Hz and 2.8 Hz, 1H), 7.64 – 7.61 (m, 3H), 7.26 (dd, *J* = 10.8 Hz, 9.0 Hz, 1H), 3.44 – 3.39 (m, 4H), 1.91 – 1.85 (m, 4H). ^{13}C NMR (500 MHz, DMSO-d_6): δ 156.3. 154.4, 146.9, 143.3, 141.6, 140.7, 138.0, 133.0, 131.2, 129.7, 127.6, 121.6 (d, *J* = 7.9 Hz), 120.4, (d, *J* = 13.1 Hz), 119.5 (d, *J* = 2.8 Hz), 116.0 (d, *J* = 22.5 Hz), 114.9 (d, *J* = 14.9 Hz), 46.2, 25.5, 19.0. HRMS (ES+): m/z $[M+H]^+$ calculated for $C_{21}H_{23}FN_6O_2$, 403.1683; found, 403.1693.

j. Chemical synthesis of compound **8**:

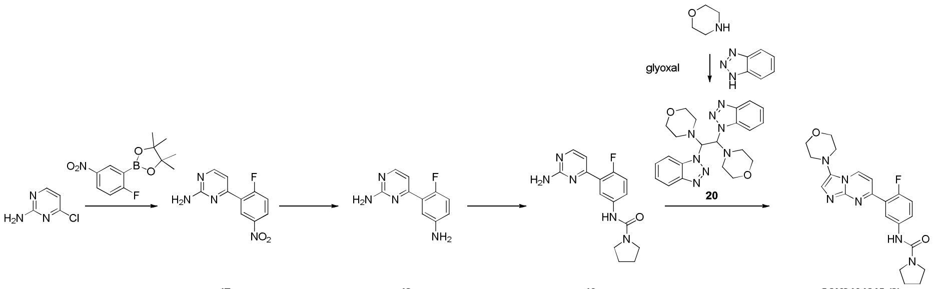

Scheme S8. Synthesis of compound **8**.

Synthesis of 4-(2-fluoro-5-nitrophenyl)pyrimidin-2-amine (**17**).

The reaction was carried out on three 1.55 mol batches: To a suspension of 4-chloropyrimidin-2-amine (200 g, 1.55 mol) and 2-(2-fluoro-5-nitrophenyl)-4,4,5,5-tetramethyl-1,3,2-dioxaborolane (413.9 g, 1.55 mol) in dioxane (5.2 L) and H_2O (1.3 L) was added $NaHCO_3$ (260.4 g, 3.1 mol) and $Pd(PPh_3)_4$ (89.45 g, 77.52 mmol) under nitrogen and stirred at 90 ^{0}C for 12 h. After cooling to 45 ^{0}C the three batches were combined, filtered, and the filter cake washed with EtOAc (3 x 1 L). The filtrate was concentrated to remove most of the dioxane and extracted with EtOAc (3 x 3 L). The combined organics were washed with brine, dried over Na_2SO_4 and concentrated. DCM was added and the resulting solid collected and dried to give **17** (520 g, 2.22 mol, 46 %). ^1H NMR (400 MHz, DMSO-d_6): δ 8.79 – 8.95 (m, 1H), 8.40 (d, *J* = 4.41 Hz, 1H), 7.59 – 7.71 (m, 1H), 7.05 (br s, 1H), 6.93 (br s, 2H). m/z = 235 $(M+H)^+$.

Synthesis of 4-(5-amino-2-fluorophenyl)pyrimidin-2-amine (**18**).

The reaction was carried out on three batches of **17** (2 batches x 0.86 mol + 1 batch x 0.43 mol. eg. a total 500 g, 2.15 mol of **17** was used): To a stirred mixture of 4-(2-fluoro-

5-nitrophenyl)pyrimidin-2-amine (**17**, 200 g, 0.86 mol) and iron (220.2 g, 3.93 mol) in EtOH (1 L) / 1,4-dioxane (1 L) at 70 ^{0}C, was added a solution of NH_4Cl (164.6 g, 53.5 mol) in H_2O (1 L) and stirring continued at 70 ^{0}C for 4 h. The reaction mixture was cooled to RT, diluted with EtOAc (2 L) and filtered. The filter cake was washed with EtOAc (3 x 500 mL).

The combined organics from all three batches were concentrated and the crude material recrystallized from EtOAc to give **18** (330 g, 1.62 mol, 75%) as pale yellow solid. ^{1}H NMR (400 MHz, DMSO-d_6): δ 8.85 (dd, J = 6.62 and 3.09 Hz, 1H), 8.42 – 8.31 (m, 2H), 7.63 (dd, J = 10.36 and 9.04 Hz, 1H), 7.04 (dd, J = 5.29 and 2.65 Hz, 1H), 6.91 (s, 2 H). m/z = 205 $(M+H)^+$.

Synthesis of N-(3-(2-aminopyrimidin-4-yl)-4-fluorophenyl)pyrrolidine-1-carboxamide (**19**).

The reaction was carried out on two 0.48 mol batches each: To a solution of 4-(5-amino-2-fluorophenyl)pyrimidin-2-amine (**18,** 100 g, 0.48 mol) and pyridine (100 g, 0.49 mol) in MeCN (1.2 L) was added a solution of phenyl chloroformate (76.47 g, 0.49 mol) at 0 ^{0}C and stirred at RT for 12 h. The reaction mixtures were combined, poured into water and filtered. The filter cake washed with EtOH (2 x 500 mL) and dried to give intermediate phenyl (3-(2-aminopyrimidin-4-yl)-4-fluorophenyl) carbamate (220 g, 0.68 mol, 69%) as a pale red solid. ^{1}H NMR (400 MHz, DMSO-d_6): δ 10.40 (br s, 1H), 8.31 (d, J = 5.09 Hz, 1H), 8.10 (br s, 1H), 7.56 (d, J = 8.22 Hz, 1H), 7.48 – 7.38 (m, 2 H), 7.34 – 7.24 (m, 2H), 7.22 (d, J = 8.22 Hz, 3H), 6.91 (br s, 1H), 6.75 (br s, 2H). m/z = 325 $(M+H)^+$.

For the synthesis of **19**, the intermediate was split into two 0.34 mol batches each: To a mixture of intermediate phenyl (3-(2-aminopyrimidin-4-yl)-4-fluorophenyl) carbamate (110 g, 0.34 mol) in dry DCM (1.5 L) at 15 ^{0}C was added pyrrolidine (28.9 g, 0.41 mol) and the mixture stirred at 15 ^{0}C for 12 h. The batches were combined, diluted with DCM (2 L), poured into water and the layers separated. The organic layer was washed with brine, dried over Na_2SO_4 and concentrated to give crude product, which was recrystallized from EtOH to give **19** (150 g, 0.49 mol, 76%) as pale yellow solid. ^{1}H NMR (400 MHz, DMSO-d_6): δ 8.35 – 8.24 (m, 2H), 8.02 (dd, J = 6.62 and 2.21 Hz, 1H), 7.68 – 7.57 (m, 1H), 7.16 (t, J = 9.92 Hz, 1H), 6.89 (d, J = 2.65 Hz, 1H), 6.66 (br s, 2H), 3.34 (br s, 4H), 1.83 (br s, 4H). m/z = 302 $(M+H)^+$.

Synthesis of 1,2-bis(1H-benzo[d][1,2,3]triazol-1-yl)-1,2-dimorpholinoethane (**20**).

The reaction was carried out in two parallels. To a mixture of benzotriazole (200 g, 1.68 mol) in EtOH (2 L) was added morpholine (146 g, 1.68 mol) at 15 ^{0}C. After stirring for 30 min, 40% aq. glyoxal in water (121.8 g, 0.84 mol) was added at 15°C and the mixture stirred for 12 h during which time a precipitate formed.

The solid was filtered, combined and washed with EtOH (3 x 500 mL) to give 1,2-bis(1*H*-benzo[*d*][1,2,3]triazol-1-yl)-1,2-dimorpholinoethane (**20**, 610 g, 1.4 mol, 83%) as a white solid. ^{1}H NMR (400 MHz, $CDCl_3$): δ 8.20 – 8.15 (m, 2H), 7.7 – 7.5 (m, 4H), 7.5 – 7.35 (m, 2H), 6.6 (br s, 2H), 3.35 – 3.2 (m, 4H), 3.15 – 2.9 (m, 4H), 2.7 – 2.5 (m, 8H).

Synthesis of N-(4-fluoro-3-(3-morpholinoimidazo[1,2-a]pyrimidin-7-yl)phenyl)pyrrolidine-1-carboxamide (**8**).

The reaction was carried out on three batches of **19** (2 x 0.2 mol, 1 x 0.1 mol, eg. a total of 150 g, 0.5 mol was used): To a suspension of *N*-(3-(2-aminopyrimidin-4-yl)-4-fluorophenyl)pyrrolidine-1-carboxamide (**19,** 60 g, 0.2 mol) and Zinc bromide (44.8 g, 0.2 mol) in DCM (2 L) was added 1,2-bis(1*H*-benzo[*d*][1,2,3]triazol-1-yl)-1,2-dimorpholinoethane (**20,** 172.7 g, 0.4 mol) at 15 ^{0}C and the reaction mixture heated to 60°C for 12 h. The batches were cooled to RT, combined, diluted with DCM (2 L) and treated with 2M NaOH solution (3 L). The mixture was stirred for 30 min, and then separated. The organic layers were concentrated to give crude material which was chromatographed (DCM / MeOH 100:1 to 10:1). Fractions containing product were combined and chromatographed a second time (EtOAc / MeOH 100:1 to 10:1). Fractions containing product of >99% purity were combined and concentrated, whilst fractions containing product of <99% purity were combined, concentrated and recrystallized from EtOAc. The crude liquors were further purified by prep-HPLC (acidic), re-crystallization from EtOAc and a second prep-HPLC (acidic). All batches of >99% purity were combined to give **8** (48.05 g, 0.12 mol, 29%). ^{1}H NMR (400 MHz, DMSO-d_6): δ 8.71 (d, *J* = 7.06 Hz, 1H), 8.37 (s, 1H), 8.17 (dd, *J* = 7.06 and 2.65 Hz, 1H), 7.74 (dt, *J* = 8.82 and 3.53 Hz, 1H), 7.47 (s, 1H), 7.38 (dd, *J* = 7.28 and 1.98 Hz, 1H), 7.21 (dd, *J* = 11.47 and 8.82 Hz, 1H), 3.85 – 3.73 (m, 4H), 3.39 – 3.31 (m, 4H), 3.04 – 2.96 (m, 4H), 1.82 (t, *J* = 6.40 Hz, 4H).). ^{13}C NMR (500 MHz, DMSO-d_6): δ 154.8, 154.4, 151.5, 144.5, 138.0 (d, *J* = 2.3 Hz), 134.0, 131.8, 125.2 (d, *J* = 11.8 Hz), 123.5, 123.2 (d, *J* = 8.2 Hz), 121.2, 116.8 (d, *J* = 24.1 Hz), 108.8 (d, *J* = 11.4 Hz), 66.6, 51.8, 46.2, 25.5. HRMS (ES+): m/z $[M+H]^{+}$ calcd for $C_{21}H_{23}FN_6O_2$, 411.1945; found, 411.1940.

2. CAD solubility assay

GSK in-house kinetic solubility assay: 5 mL of 10 mM DMSO stock solution was diluted to 100 µL with pH7.4 phosphate buffered saline, equilibrated for 1 h at room temperature and filtered through Millipore Multiscreen HTS-PCF filter plates (MSSL BPC). The filtrate was quantified by suitably calibrated Charged Aerosol Detector[3]. The upper limit of the solubility was 500 µM when working from 10 mM DMSO stock solution.

3. Solubility of Solid Compounds in Fasted Simulated Intestinal Fluid (FaSSIF).

This experiment determines the solubility of solid compounds in fasted simulated intestinal fluid (FaSSIF) at pH 6.5 after 4 h equilibration at RT. Then 1 mL of FaSSIF buffer (3 mM sodium taurocholate, 0.75 mM lecithin in sodium phosphate buffer at pH 6.5) was added to manually weighed 1 mg of solid compound in a 2 mL HPLC auto-sampler vial. The resulting suspension is shaken at 900 rpm for 4 h at RT and then transferred to a Multiscreen HTS, 96-well solubility filter plate. The residual solid was removed by filtration. The supernatant solution was quantified by HPLC-UV using single-point calibration of a known concentration of the compound in DMSO. The dynamic range of the assay was 1-1000 µg/mL.

4. Parasite assays

T. cruzi[4], *Leishmania donovani* axenic amastigote, cidal axenic amastigote and intra-macrophage drug sensitivity assays were conducted as previously described[5,6].

a. Rate of Kill

The rate of kill of compound **8** was determined in axenic amastigotes using the previously described cidal axenic assay[5], with one minor modification. Instead of a single readout at 72 h, multiple identical plates were used and read at different time points (24, 48, 72 h). Rate of kill curves showing the luminescence signal at each time point for each concentration were plotted.

5. Intrinsic clearance (CLint)

Test compound (0.5 µM) was incubated with female CD1 mouse liver microsomes (Xenotech LLC ™; 0.5 mg/mL 50 mM potassium phosphate buffer, pH7.4) and the reaction started with addition of excess NADPH (8 mg/mL 50 mM potassium phosphate buffer, pH7.4). Immediately, at time zero, then at 3, 6, 9, 15 and 30 minutes an aliquot (50 µL) of the incubation mixture was removed and mixed with acetonitrile (100 µL) to stop the reaction. Internal standard was added to all samples, the samples centrifuged to sediment precipitated protein and the plates then sealed prior to UPLCMSMS analysis using a Quattro Premier XE (Waters Corporation, USA).

XLfit (idbs, UK) was used to calculate the exponential decay and consequently the rate constant (k) from the ratio of peak area of test compound to internal standard at each time-point. The rate of intrinsic clearance (CLint) of each test compound was then calculated using the following calculation:

CLint(mL/min/g liver) = k x V x Microsomal protein yield

Where V (mL/mg protein) is the incubation volume/mg protein added and microsomal protein yield is taken as 52.5 mg protein/g liver. Verapamil (0.5 µM) was used as a positive control to confirm acceptable assay performance.

6. Pharmacokinetic studies.

a. Mouse and dog PK

Mouse pharmacokinetic studies were conducted in male CD-1 mice and male beagle dogs. 3 animals per group were used for each study performed.

A dose of 3 mg/kg was administered intravenously, in a bolus form to mice and infused over a 30 min period to dogs. For all the intravenous studies compound were dissolved in 5% DMSO/20% kleptose in saline.

A dose of 10 mg/kg was orally administered by gavage to the two species in a suspension of 1% methylcellulose to investigate oral pharmacokinetics.

Peripheral blood samples were obtained at 5, 15, 30 min, 1, 2, 4, 6 ,8 and 24 h after intravenous administration and 15, 30, 45 min,1, 2, 4, 6, 8 and 24 h after oral administration. Blood was 1/2 diluted with milliQ water and immediately frozen on dry ice until analysis. Quantification was performed by means of LC-MS/MS (API4000), with a lower limit of quantification of 1 ng/mL.

Pharmacokinetic parameters, namely clearance (CL), volume of distribution at steady state (Vss) and bioavailability (F%), were estimated using Phoenix, WinNonLin Version 6.3.

b. Rat PK

Male Sprague Dawley rats (n=3) were dosed either intravenously at a target dose of 3 mg/kg or orally at a target dose of 10, 30, 100 and 300 mg/kg. Following discrete intravenous tail vein dosing from a solution of 5% DMSO/20% kleptose in saline, serial blood samples (~10 µl) were taken via lateral tail vein at 0.01, 0.05, 0.08, 0.25, 0.5, 1, 2, 4, 6, 8 and 24 h post dose and 1/9 diluted with miliQ water, frozen on dry ice, then stored at approximately –20 ºC or below until analysis.

Following discrete oral gavage dosing from a suspension of 1% methyl cellulose at target doses of 10, 30, 100 and 300 mg/kg, respectively, serial blood samples (10 µl) were taken via lateral tail vein at 0.08, 0.25, 0.5, 0.75, 1, 2, 4, 6, 8 and 24 h post dose and 1/9 diluted with miliQ water, frozen on dry ice, then stored at approximately –20°C or below until analysis.

Samples were analyzed for parent compound using a method based on protein precipitation followed by UPLC-MS/MS.

Pharmacokinetic parameters, namely clearance (CL), volume of distribution at steady state (Vss) and bioavailability (F%), were estimated using Phoenix, WinNonLin 6.3.

c. Data Analysis

Data analysis and calculation of pharmacokinetic parameters was performed using Phoenix, WinNonLin Version 6.3.

Following the intravenous administration, the whole blood clearance was calculated by determining the dose administered to each animal and dividing by the AUC0-∞ . The estimate of the volume of distribution at steady state (VSS) was calculated as MRT*CL, where MRT is the mean residence time, calculated by AUMC0-∞ /AUC0-∞.

For both intravenous and oral administration, the systemic exposures were determined by calculating the area under the blood concentration time curve (AUC) from the start of dosing to the last observed quantifiable concentration (AUC0-t) by using the linear up-log down trapezoidal rule. The slope of the terminal elimination phase was estimated by linear regression of the terminal data points (minimum 3 points) from a natural log concentration versus time plot of the data. The half-life ($t_{1/2}$) of the terminal elimination phase was calculated as $t_{1/2} = 0.693/\lambda$.

7. *In vivo* efficacy in female Balb/C mice

To establish infection in mice, sodium stibogluconate-sensitive *L. donovani* (LV9, WHO designation: MHOM/ET/67/HU3) amastigotes were isolated from the spleen of a heavily infected donor hamster (Janvier, France)[7]. An inoculum containing 1.0 x 10^8 amastigotes/mL in Dulbecco's modified eagles medium (high glucose) was prepared

and female BalbC mice (Harlan, UK) then infected intravenously via the tail vein with a 0.2 mL bolus (equivalent to ~2.0 x 10^7 amastigotes). Infection was left to establish for 7 days. At day 7, mice (n=5/dose group) were treated with compound **8** once or twice daily orally as a suspension in 0.5% hydroxypropylmethylcellulose, 0.4% tween 80 and 0.5% benzyl alcohol. With twice daily regimens, the second daily dose was administered approximately 8 h following the first dose. Dose regimens evaluated were:

1. 50 mg/kg twice daily for 5 days
2. 25 mg/kg, 10 mg/kg and 3 mg/kg twice daily for 10 days
3. 25 mg/kg once daily for 10 days

Miltefosine, as the only oral treatment for visceral leishmaniasis, was used as a positive control in each evaluation, dosed once daily at 30 mg/kg as solution in sterile water. 30mg/kg was chosen as it represents the ED_{90} in mice[8,9] and therefore the required benchmark by which to compare any potential new oral therapies. For reference, a detailed study on the pharmacokinetics of miltefosine in mice has recently been published[10]. The minimal efficacy required by DNDi is 95% reduction in parasitaemia[11]. Vehicle dosed animals formed the negative control group.

Spot blood samples were taken for bioanalysis from each dose group (n=3/5 mice) following the first dose at day 1 and the last day of dosing.

Three days following completion of each dosing regimen, all mice were culled, liver smears prepared from each animal and stained with Rapi-diff II (Biostain Ready Reagents, UK). The number of amastigotes/500 liver cells were then counted microscopically and parasite load expressed in Leishman Donovan units (LDU): the mean number of amastigotes per 500 liver cells x mg liver. Expression of parasite load as LDU is the standard approach for assessing efficacy[12,13]. The individual animal LDU following oral dosing of compound **8** at 50 mg/kg b.i.d. for 5 days, 25 mg/kg b.i.d., 10 mg/kg b.i.d. and 3 mg/kg b.i.d. for 10 days and 25 mg/kg u.i.d. for 10 days were determined.

8. Preclinical safety studies

a. Genotoxicity

i. Bacterial Mutation (Ames) Assay

Compound **6** was tested in a bacterial mutation screening assay (Ames test) with *Salmonella typhimurium* TA1535, TA1537, TA98, TA100 and *Escherichia coli* WP2uvrA(pKM101) in the presence and absence of an exogenous mammalian oxidative metabolism system (S9-mix). The maximum concentration tested and analysed was 2500 µg per plate, limited by precipitation and in accordance with current guidelines (OECD 471, 1997[14]). Compound **6** was found not mutagenic in this bacterial mutation screening assay when tested in the presence and absence of S9-mix.

Compound **6a** was tested in a bacterial mutation screening assay (Ames test) with *Salmonella typhimurium* TA1535, TA1537, TA98, TA100 and *Escherichia coli* WP2uvrA(pKM101) in the presence and absence of an exogenous mammalian

oxidative metabolism system (S9-mix). The maximum concentration tested and analysed was 1500 µg per plate, limited by precipitation. Compound **6a** was found mutagenic in strain TA1537 when tested in the absence of S9-mix.

Compound **7a** was tested in a bacterial mutation screening assay (Ames test) with *Salmonella typhimurium* TA1535, TA1537, TA98, TA100 and *Escherichia coli* WP2uvrA(pKM101) in the presence and absence of an exogenous mammalian oxidative metabolism system (S9-mix). The maximum concentration tested and analysed was 1500 µg per plate, limited by precipitation. Compound **7a** was found not mutagenic in this bacterial mutation screening assay when tested in the presence and absence of S9-mix.

Compound **8** was tested in a bacterial mutation screening assay (Ames test) with *Salmonella typhimurium* TA1535, TA1537, TA98, TA100 and *Escherichia coli* WP2uvrA(pKM101) in the presence and absence of an exogenous mammalian oxidative metabolism system (S9-mix). The maximum concentration tested and analysed was 5000 µg per plate, in accordance with current guidelines (OECD 471, 1997[14]).

The bacterial mutation screening assay was a plate incorporation test carried out as previously described[15] and according to current methodology.

ii. Mouse Lymphoma Assay

Compound **8** was negative in the mouse lymphoma screen when tested for 3 hours in the presence of S9-mix and 24 hours in the absence of S9-mix. The maximum concentrations analysed were 410.45 µg/mL (1 mM, the maximum concentration in accordance with current guidelines and 100 µg/mL (limited by toxicity), for the 3 and 24 hour treatments, respectively (OECD 476, 1997[16])).

The mouse lymphoma screen was carried out using test methodology based on established procedures for *in vitro* gene mutation testing[17,18,19,20].

b. 7 day rat toxicology studies

A 7 day oral repeat-dose study in male rats was conducted to determine the toxicity and toxicokinetics of compound **8**. Groups of male rats (4/group toxicity; 3/group toxicokinetics) were given 0, 100, 300 or 1000 mg/kg/day compound **8** once daily for up to 7 days by oral gavage. In animals given 100, 300 and 1000 mg/kg/day, blood concentrations of compound **8** were generally quantifiable to 24 h after dosing on both day 1 and day 7 (1000 mg/kg/day animals were not sampled on day 7 due to early termination). Systemic exposures ($AUC_{0\text{-}t}$ and Cmax) increased in an approximately proportional manner with increasing dose on Day 1. On Day 7 exposures increased in a greater than proportional manner between 100 and 300 mg/kg/day. This was driven by the increase in systemic exposure from Day 1 to Day 7 at the 300 mg/kg/day dose. There is a 37-fold margin at 300 mg/kg/day, where minimal microscopic and clinical pathology changes were observed, to the predicted human blood $AUC_{0\text{-}24}$ of 27 ng/h/mL after a 600 mg dose b.i.d.

The 1000 mg/kg/day dose level was not tolerated with all animals terminated early due to the clinical signs observed. The 100 and 300 mg/kg/day dose levels were well

tolerated over the 7 days. Microscopic (mild to moderate) changes were observed at 1000 mg/kg/day in the kidney, liver and pancreas. At 300 mg/kg/day minimal/mild microscopic changes were observed in the liver (increased incidence of PAS reactivity of hepatocytes compared to control [no evidence of hepatocellular rarefaction on H&E stained sections]), and in the pancreas of a single animal at this dose (diffuse acinar cell degranulation). At 1000 mg/kg/day increases in alanine aminotransferase, glutamate dehydrogenase, alkaline phosphatase, glucose, phosphorus, calcium and a reduction in chloride concentrations were seen. A lower urea concentration was seen in a single male. Increases were seen in total bile acids (≥100 mg/kg/day), total bilirubin and cholesterol (≥300 mg/kg/day) and creatinine (300 mg/kg/day only). Individual males given 100 or 300 mg/kg/day had increased reticulocyte counts with correlating increases in medium and high reticulocyte subpopulations.

9. Mode of action studies

a. Cell lines and culture conditions

The clonal *Leishmania donovani* cell line LdBOB (derived from MHOM/SD/62/1S-CL2D) was grown as promastigotes at 26°C in modified M199 media[21]. Bloodstream-form *T. brucei*, Lister 427 and 2T1 cells[22] were grown in HMI-9T medium[23].

b. RITseq library screening

An RNAi library screen was carried out as described previously[22]. Briefly, the RNAi library was induced on day 0 with tetracycline (1 µg/mL) and maintained under blasticidin (1 µg/mL) and phleomycin (1 µg/mL) selection at a minimum of 2.5×10^7 cells in 150 ml of media. Following induction for 24 h, cultures were selected with 6.3 nM compound **7** (~3x EC_{50}) was added. Cultures were split and supplemented with fresh drug as required. DNA was extracted from drug-resistant cells on day 12. RNAi target fragments were then amplified by PCR using the LIB2f and LIB2r primers (Table S6). For high throughput identification of fragments, the PCR products were fragmented and sequenced using an Illumina HiSeq platform at BGI (Beijing Genomics Institute). Reads were mapped to the *T. brucei* 927 reference genome (v9.0, tritrypdb.org) with Bowtie 2[24] using the following parameter: very-sensitive-local-phred33. The generated alignment files were manipulated with SAMtools[25] and a custom script to identify reads with barcodes (GCCTCGCGA)[22]. Total and bar-coded reads were then quantified using the Artemis genome browser[26]. Hit-lists generated from RIT-seq analyses excluded selected large gene families, including variant surface glycoproteins, and genes immediately adjacent to hits.

c. Generation of drug-resistant parasites

Compound **7**-resistant lines were generated by sub-culturing a clone of wild-type *L. donovani* in the continuous presence of this compound. Starting at a sub-lethal concentration of 3 nM compound **7**, the drug concentrations in 3 independent cultures were increased in a step-wise manner, usually by 2-fold. After a total of 120 days in culture, when promastigotes were able to survive and grow in >100 nM compound **7**, the resulting cell lines were cloned by limiting dilution in the absence of compound. Three clones (RES I-III) were selected for further biological study.

d. DNA sequencing

Genomic DNA was harvested from three independently generated *L. donovani* resistant clones and an age match control, as previously described[27]. PCR was performed using Platinum PCR Supermix (Invitrogen), as per manufacturer's instructions. To sequence the β4 subunit (LdBPK_360340.1), an 889bp fragment was generated using primers SubunitB4FW and SubunitB4RV, then sequenced using primer SubunitB4FW (Table S6). To sequence the β5 subunit (LdBPK_361730.1), an 1113bp fragment was generated using primers Subunitβ5FW and Subunitβ5RV, then sequenced using primer SubunitB5FW. PCR amplification and DNA sequencing were performed in triplicate for each independently generated cell line. The *Leishmania donovani* BPK282A1 sequences of the two subunits were identified in TriTrypDB and were a direct match with the age match control (drug-sensitive).

e. Generation of overexpression constructs and LdBOB transgenic cell lines

The genes encoding the mutated versions of the β4 (LdBPK_360340.1) and the β5 (LdBPK_361730.1) subunits of the proteasome, identified in our drug-resistant parasites, were synthesised (GeneArt, Thermo Fischer Scientific). The synthetic genes, flanked by BamHI sites were cloned into the equivalent site of pIR1-SAT. The accuracy of all assembled constructs was verified by sequencing.

Mid-log-phase *L. donovani* promastigotes (LdBOB) were transfected with overexpression constructs using the Human T-Cell Nucleofector kit and the Amaxa Nucleofector electroporator (program V-033). Following transfection, cells were allowed to grow for 16-24 h in modified M199 medium with 10% fetal calf serum prior to appropriate drug selection (100 µg/mL nourseothricin). Cloned cell lines were generated by limiting dilution, maintained in selective medium, and removed from drug selection for one passage prior to experiments.

f. *T. brucei* RNAi constructs and cell lines

PCR primers (Table S6) were designed using RNAit[28] to generate a 500-bp fragment conferring specific knockdown to Tb927.8.6620 (Table S7) and a 543-bp fragment of Tb927.9.15260 PCR fragments were cloned in the pRPaSLi construct for the generation of stem-loop dsRNA under the control of tetracycline as the trigger for RNAi[28,29]. Constructs were digested with AscI, EtOH-precipitated, and resuspended (1 µg/mL) in sterile water. 2T1 strains, containing a tetracycline repressor, were electroporated using program X-001 of the Nucleofector II electroporator (Amaxa, Cologne, Germany)[30] following the addition of 5 µg of linearized DNA mixed in 100 µL of cytomix[31]. Transformants were cloned by limiting dilution under phleomycin (1 µg/mL) and hygromycin (2.5 µg/mL) selection. Puromycin susceptibility (1 µg/mL) was tested for full integration of the construct and expression of stem-loop RNAi was induced with concentrations of tetracycline ranging from 1 ng – µg/mL.

g. Quantitative RT-PCR

T. brucei RNA was isolated using an RNeasy purification kit (Qiagen) and cDNA-synthesized using a high capacity RNA-to-cDNA kit (Applied Biosystems). PCR primers (Table S6) were designed using the Premierbiosoft's Beacon Designer 6. qRT-PCRs

consisted of 1 µL (40 ng) of cDNA, 10 µL of Brilliant III Ultra-Fast QPCR Master Mix (Agilent Technologies), 1 µL (500 nM) each of the forward and reverse primers, and 0.3 µl (30 nM) of reference dye and nuclease-free PCR grade-treated water. PCR was performed using an Agilent Mx3005P machine with the following cycling conditions: 95°C for 3 min; 40 cycles of 95°C for 20 s; then 60 °C for 20 s. The reference gene *TERT* (Tb927.11.10190, telomerase reverse transcriptase) was used to provide a baseline of transcription levels for normalization of the data. Relative quantification in the tetracycline-induced cell lines were normalized to the un-induced cell line using the $\Delta\Delta Ct$ method.

h. Morphological analysis

For transmission electron microscopy, *L. donovani* promastigotes were prepared as described previously[32], post-fixed for 1 h at 4°C with 1% (v/v) osmium tetroxide in 100 mM phosphate buffer (pH 6.5) and rinsed briefly in water prior to fixing or staining *en bloc* with 3% (v/v) aqueous uranyl acetate. Cells were rinsed further in distilled water and subsequently dehydrated through a graded EtOH series with a final wash in propylene oxide, prior to embedding in Durcupan resin. Sections were stained with 3% (v/v) aqueous uranyl acetate and Reynold's lead citrate prior to examination using a JEOL-1200 EX TEM.

i. FACS analysis

Cultures of bloodstream *T. brucei* and *L. donovani* promastigotes at starting cell densities of 5 x 10^5 were incubated with concentrations of compound **7** equivalent to 5x their respective EC_{50} values. At defined intervals samples of culture were taken, cells were collected by centrifugation (900 g, 10 min, 4°C) and washed twice in PBS before preparation for FACS analysis[33]. After washing, cell densities were adjusted to 5 × 10^5 cells mL, re-suspended in 500 µL of PBS containing 50 µg/mL propidium iodide, 50 µg/mL RNase and 0.1% Triton X-100, incubated at room temperature for 20 min in the dark and analysed using FACSort analytical flow cytometer using Cellquest software (BD Biosciences).

j. Proteasome enrichment by ultracentrifugation

The protocol described by Kisselev *et al*.[34] was adapted to enrich the proteasomes present in *L. donovani* and THP1 crude extracts. *L. donovani* or THP1 cells were resuspended in sucrose buffer (50 mM Tris buffer pH 7.5, 5 mM Magnesium chloride, 1 mM EDTA, 50 mM NaCl, 250 mM sucrose, 2 mM ATP, 1 mM DTT) and lysed by nitrogen cavitation for 15 min (110 bars of 1500 psi). Lysates were centrifuged at 20,000g for 30 min at 4ºC and supernatants containing soluble protein were subjected to an ultracentrifugation step at 300,000g for 2h at 4ºC. The resulting proteasome-containing pellets were resuspended in sucrose buffer and left on ice for 30 min to complete solubilization. Samples were centrifuged for 10 min at 20,000 g to remove insoluble material. Proteins contained in supernatant (proteasome enriched fraction) were measured by Bradford.

k. Proteasome activity assays

Compounds were dispensed into black 384-well assay plates (Greiner) by acoustic dispensing (LabCyte ECHO). For potency determinations, eleven-point, one in three dilution curves were generated, with a top concentration of 100 µM. Three proteasome activities were measured using a commercially available indirect enzyme-based luminescent assay kit (Promega) with Suc-LLVY-aminoluciferin, Z-LRR-aminoluciferin and Z-nLPnLD-aminoluciferin used as substrates for chymotrypsin-like, trypsin-like and caspase-like activities, respectively. In addition to proteasome samples: *L. donovani* and THP1-enriched proteasomes, commercially available human purified 26S proteasome (Boston Biochem) was also assessed. Proteasome final assay concentration were 0.025 mg/ml for *L. donovani* and THP1-enriched proteasomes and 1 nM for human purified 26S proteasome. Proteasome solution was added to the plates containing the compounds one hour before adding the proteasome substrate solution. Substrate solution was then added and after 30 min of incubation luminescent signal was measured in an Envision reader (PerkinElmer).

l. Western blot analysis

Cultures of *L. donovani* axenic amastigotes were diluted to either 8 x 10^6 or 8 x 10^7 cells/ml prior to treatment with compound **8** at 30 nM (0.1X whole cell IC_{50}), 300 nM (1X IC_{50}) or 3100 nM concentrations equivalent to 0.1, 1 and 10X the compounds EC_{50} value. Culture were incubated with compound **8** for 1h at 37ºC. Miltefosine at 80 µM (equivalent to 10X EC_{50} value) and bortezomib at 200 nM (equivalent to 10X EC_{50} value) were used as negative and positive controls, respectively. Following incubation, drug-treated parasites were pelleted by centrifugation at 640 g for 15 min and washed twice with cold PBS. Untreated cells were also harvested alongside treated cells to assess the basal levels of ubiquitylated proteins. Pellets were frozen at -80ºC and three cycles of freeze/thawing were conducted to biologically inactivate parasites. Lysis buffer (50 mM Tris-HCl pH 8.5, 150 mM NaCl, 5 mM EDTA, 1% Triton X-100, 1 mM PMSF and complete mini EDTA protease inhibitor cocktail) was added to each pellet prior to one fast cycle of freeze/thawing. Lysates were cleared by centrifugation (10 min, 20,000g, 4°C) and supernatants were collected. Protein concentrations were determined by standard Bradford assays and Laemmli sample buffer was added to samples. For each condition, 80 µg of each protein sample was separated by SDS-PAGE. Proteins were then transferred onto polyvinylidene difluoride (PVDF) membranes (Roche), blocked with milk at 5% in TBS-tween 0.1% and incubated with anti-ubiquitin P4D1 antibody (Santa Cruz) diluted 1:1,000 in 2.5% TBS-tween 0.1%.or anti-tubulin 1:1,000 (Sigma)

m. Activity-Based Probe Labelling of THP1 and *L. donovani* proteasome

Proteasome enriched fractions from THP1 (20 µg), *L. donovani* (10 µg) and *L. donovani* resistant RES II cells (14 µg) were incubated with compound **8** or bortezomib at 100 µM for 1 h. Proteasome enriched fractions were also incubated with DMSO as control. For all proteasome labelling experiments, the active-site probe Me4BodipyFL-Ahx3Leu3VS (UbiQ-018) was used at final concentration of 500 nM in sucrose buffer (50 mM Tris buffer pH 7.5, 5 mM magnesium chloride, 1 mM EDTA, 50 mM NaCl, 250 mM sucrose, 2 mM ATP, 1 mM DTT). Proteasome enriched fractions treated with

DMSO or test compounds, were incubated with the probe for 2h at RT. Samples were then denatured by addition of Laemmli sample buffer, heated to 95°C for 10 min and separated by electrophoresis on a 15% SDS-PAGE acrylamide gel. Following separation, gels were scanned in the fluorescein channel of a ChemiDoc reader (λ excitation/emission = 480/530 nm). Fluorescent proteins were excised and MALDI–TOF analysis carried out by the FingerPrints Proteomics service at the University of Dundee (see Table S8).

10. Measuring Proteolytic activity of *Leishmania tarentolae* Proteasome

The chymotrypsin-like catalytic activity of purified *Leishmania tarentolae* proteasome was monitored by measuring the cleavage of a Rhodamine110-labelled fluorogenic substrate (Suc-LLVY-Rh110-dPro), which alleviates a quench of the Rhodamine110 fluorophore leading to an increase in fluorescence at 535 nm[35]. Test compounds were pre-prepared as a 10 mM stocks in 100 % DMSO and then diluted 1 in 3, over 11 points, prior to dispense of 100 nl of each dilution series into black, low volume, 384-well microplates. Proteasome was prepared as a 0.5 µg/mL working solution in assay buffer (50 mM HEPES (pH 7.5), 150 mM NaCl, 5 mM $MgCl_2$, 5% glycerol, 1 mM CHAPS) and 5 µL was added using a Multidrop Micro (Thermo Fisher Scientific) to all wells containing compound dilutions on the test microplates. The microplates were then centrifuged for 1 min at 1000 rpm and incubated for 15 mins at 25 °C, prior to an assay initiating addition of 5 µl of fluorogenic substrate, which had been prepared as a 1 µM (~ 2x K_m) solution in assay buffer, using a Mutidrop Micro. The assay microplates were then centrifuged for 10 secs before using a Tecan M1000 Pro to monitor the increase of fluorescent intensity at 25 °C by excitation of the liberated Rhodamine110 at 485 nm and emission at 535 nm every 125 secs for 20 cycles. These data were then used to calculate initial rates of reactions for all assay wells on the microplate by fitting to a linear regression model. The initial rates of reaction for all compound concentrations tested were normalised to the initial rates determined for 16 replicate control wells per microplate of both uninhibited reaction and no-proteasome background, and subsequently, IC_{50} values were determined by fitting the normalised data to the following four-parameter logistic model; y = a + ((b – a) / (1 + (10 ^ x / 10 ^ c) ^ d), where 'a' is the minimum, 'b' is the Hill slope, 'c' is the IC_{50} and 'd' is the maximum.
Table S8 shows the *Leishmania tarentolae* activity for compound **8**.

11. Cryo-EM of 20S *Leishmania tarentolae* proteasome

a. Purification of 20S *Leishmania tarentolae* proteasome.

Leishmania tarentolae strain P10 was purchased from Jena Biosciences and cultivated in LEXSY BHI medium, according to the manufacturers' guidelines. Cells were harvested in early stationary phase by centrifugation at 3000g for 30 mins and the pellets were frozen. For the purification, the frozen cell pellets were defrosted on ice, resuspended in twice the pellet volume of 100 mM mM Tris–HCl pH 7.5, 500 mM sucrose, 2 mM EDTA, 100 mM NaCl, 10 mM $MgCl_2$, 4 mM ATP and 2 mM DTT. Cells were lysed by sonication at 10% Amplitude for 30sec on/off for 6 cycles using Soniprep 150 sonicator. The lysates were clarified by gentle centrifugation at 20,000 g for 30 min. The supernatant was further ultra-centrifuged at 300,000 × g for 2 h at 4 °C. Pelleted proteasomes were solubilised in 100mM mM Tris–HCl pH 7.5, 100 mM sucrose, 1 mM EDTA, 150 mM NaCl, 5 mM $MgCl_2$, 4 mM ATP and 1 mM DTT (GF buffer) and

incubated for 1 hour at 4 ºC. The suspension was then centrifuged at 20,000g for 30 mins at 4 ºC. The supernatant was filtered using a 0.2 µm filter and passed through GE HiPrep 16/60 Sephacryl S-400 HR column equilibrated with the GF buffer using a GE Akta Pure chromatography system. Fractions of 1.5 ml were collected and assayed for chymotrypsin-like activity using suc-LLVY-[Rh110]-[D-Pro] peptide. Active fractions were pooled and further purified by two rounds of anion exchange chromatography using GE HiScreen CaptoQ and then GE MonoQ 5/50 GL columns, equillibrated with GF buffer. Bound proteins were eluted by linear 0-1M NaCl gradient. Fractions (0.25 mL) were collected and analysed by silver stain and tested for chymotrypsin activity. The purest fractions were pooled together and loaded on GE Superose 6 Increase 5/150 GL column equilibrated with 100 mM mM Tris–HCl pH 7.5, 150 mM NaCl, 5 mM $MgCl_2$, 4mM ATP and 1 mM DTT. Peak fractions were analysed as described above. The final concentration of 0.1 mg/mL was estimated by Bradford assay using Pierce™ Coomassie Plus (Bradford) Assay Reagent.

12. Cryo-grid preparation and data collection

a. Purified proteasome was used to prepare Cryo-grids with and without compound **8**.

For Cryo-grids with proteasome alone, a concentration of 0.1 mg/mL was used. Quanitfoil grids (300 mesh, 1.3/1.2) coated with 2 nm carbon (Agar Scientific) were freshly glow discharged using a Pelco easiGlow at 25 mA for 45 s. Initially, 2.5 µl of purified proteasome was applied. After 30s incubation the grid was double-sided blotted for 0.5 s using a Vitrobot Mark IV (FEI ThermoFisher). Immediately, another 2.5 µL of sample was applied followed by 30 s incubation and 2.5s blotting and then the grid was plunged into liquid ethane. The blot force was kept constant at 2. For the liganded complex, the purified proteasome was buffer exchanged into 100 mM Tris–HCl pH 7.5, 5 mM $MgCl_2$ and 1 mM DTT and concentrated to 0.4 mg/mL using a Vivaspin 500 centrifugal concentrator MWCO 5 kDa. 20 µM of compound **8** was incubated with 0.5 µM of purified proteasome at 37 °C for 30 min. In this case, the sample was applied only once on the grid. All grids were made at 4°C and 100% humidity.

High-resolution data were collected on a Titan Krios electron microscope equipped with a Falcon III direct electron detector at ThermoFischer-Cambridge-Pharma consortium at Cambridge, Nanosciences.

i. Image processing

Beam induced motion was corrected in MotionCor2[36] retaining all the frames of the raw movies, collected at pixel size of 1.07 Å, using a patch alignment of 5x5. All subsequent particle picking, particle extraction, 2D and 3D classifications as well as 3D refinements were performed in Relion2.0[37]. Initially, particles were manually picked and 2D class averages obtained using a mask diameter of 240Å then served as references for later autopicking. CTF correction was performed using CTFFIND4[38] and the best quality integrated movies were examined and selected. Particles were extracted from dose-weighted micrographs by setting a box size of 300 pixels for all data sets. After 2-3 rounds of 2D classification, 3D classification, and 3D refinement were performed without applying any mask and using the *Thermophilic acidiphilum* proteasome structure (EMD-3231) as the initial model (filtered to 30 Å).

Fourier filtering or masking, were created using UCSF Chimera[39]. The mask was created from the final 3D auto-refinement of the particles (Figure S11 and S15) adjusted to a threshold of 0.0062 for apo and 0.0115 for liganded, low-pass filtered to 15 Å and a soft edge of three pixels was applied. The local resolution of the maps was estimated using the Relion-2.0 local resolution function, setting the same B-factor as the post processing output. No symmetry was applied at any stage of the image processing.

13. Proteasome Cryo-EM structure refinements

Two Cryo-EM structures were refined.

a. Apo and ligand structures

Structure factors of the apo dataset were generated from the sharpened EM map using Refmac[40] within the CCP4 suite of programs[41]. Molecular Replacement was carried out using Phaser[42] with the coordinates of the human proteasome crystal structure 4R3O. Model building was carried out using Coot[43], guided by sequence alignments generated for all the individual α- and β-subunits of human and *Leishmania tarentolae* proteasomes. When the higher resolution sharpened EM liganded proteasome map became available model building was switched from the apo structure to the liganded structure. The quality of this map was sufficient to allow backbone tracing for most of the protein with only a few gaps. The amino acid side chain conformations were built by selecting the best fitting rotamer followed by real space refinement in Coot. One half of the 20S proteasome subunit was manually built (subunits A-N) with the two-fold symmetry used to generate the second half (subunits O-b). A small number of water molecules were added based on standard X-ray structure criteria (significant peaks in the map, consistent with favourable locations for hydrogen bonding interactions with well-ordered protein residues). Ligand coordinates and dictionary files were generated using Grade[44]. Multiple cycles of manual model building followed by structure refinement with Refmac were carried out utilising two-fold NCS restraints throughout, initially using jelly body refinement, with restrained refinement used in the final refinement step. The refined liganded proteasome coordinates were then used as a starting point for building the apo structure (omitting the ligands and waters). The individual subunits were adjusted using rigid body refinement in Coot and manually adjusted where intradomain movements were apparent or where side chain conformations were clearly different in the two structures. The refinement protocol was similar to that used for the liganded structure. The EM maps and structures have been deposited in the Protein Data Bank: liganded structure EMD-4590 (PDB 6QM7), apo structure EMD-4591 (PDB 6QM8).

14. Molecular modelling

Using Prime[45], the homology model of *L. donovani* β4-β5 subunits was generated utilising as reference the Cryo-EM structure of *L. tarentolae* bound to compound **8**. The sequence identity between *L. tarentolae* and *L. donovani* proteasome β4-β5 subunits was 95% and 98%, respectively. The model was refined by performing a 100 ns MD simulation of the protein-ligand complex using AMBER16 suite of programs[46]. FF14SB forcefield was used for the protein. The geometry of compound **8** was refined using Gaussian 03[47] at the HF/6-31G* level. The optimized geometries were used to calculate electrostatic potential-derived charges (ESP). The forcefield parameters for the ligand

were generated with antechamber module, using the general AMBER forcefield (GAFF2.0). The site recognition software SiteMap[45] was used to describe the physicochemical properties of the ligand binding site. Water networks and relative energies were calculated using WaterFLAP (Molecular Discovery).

Supplementary figures

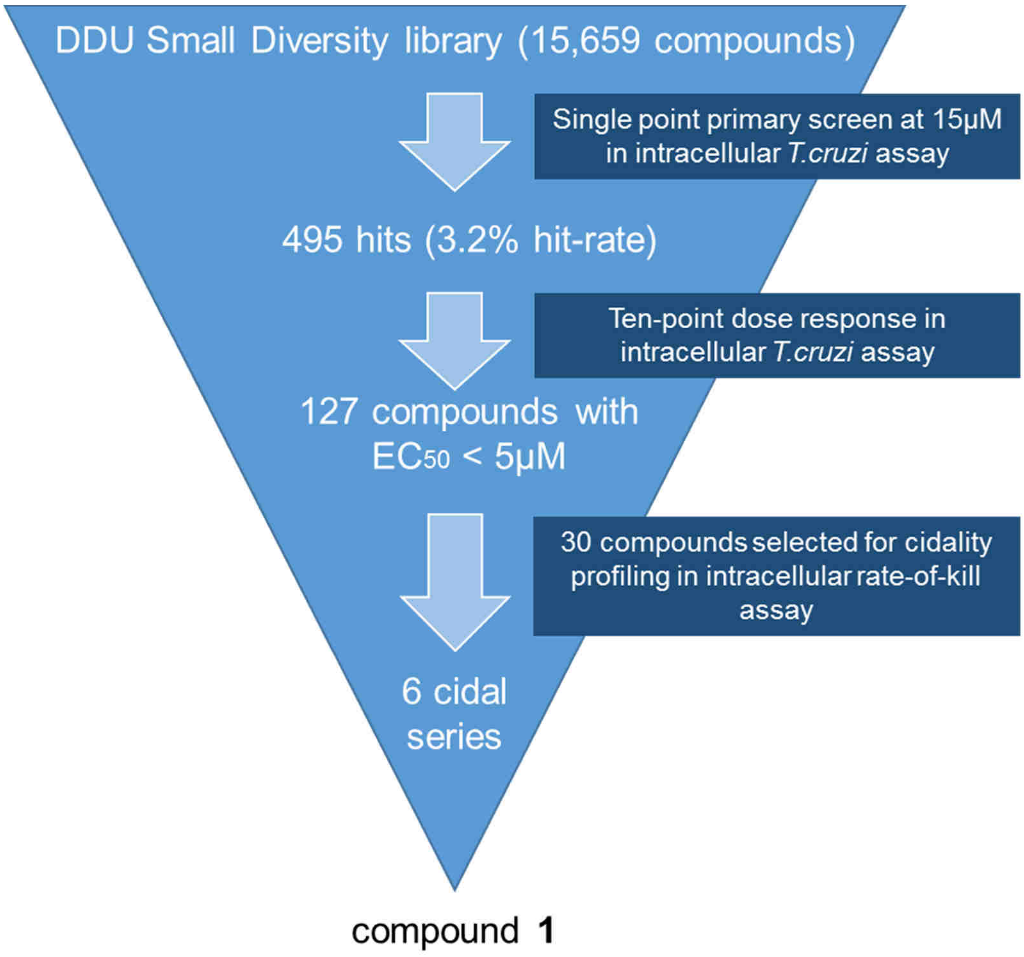

Figure S1. Screening cascade for primary *T. cruzi* screen that identified compound **1**.

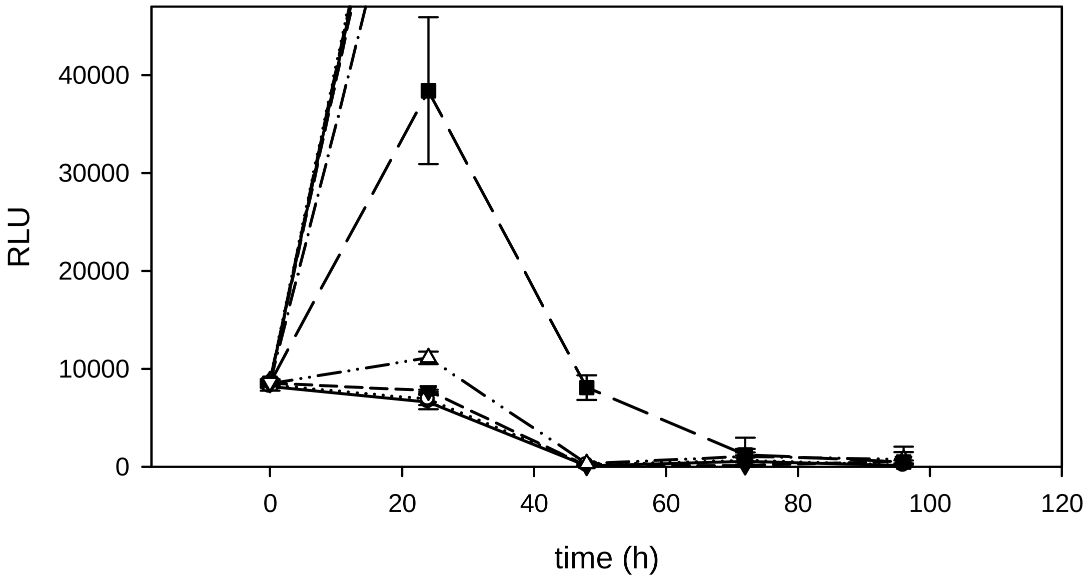

Figure S2. Axenic amastigote rate of kill time-course experiment for compound **8**. Amastigote levels (as RLU, relative luminescence units) for each tested concentration plotted against time. Data represented as mean +/- standard deviation, n=4 (technical replicates). Concentrations with inhibitory effect: —●— 50 µM, ··○·· 17 µM, —▼— 5.6 µM, —△·· 1.9 µM, —■ – 620 nM.

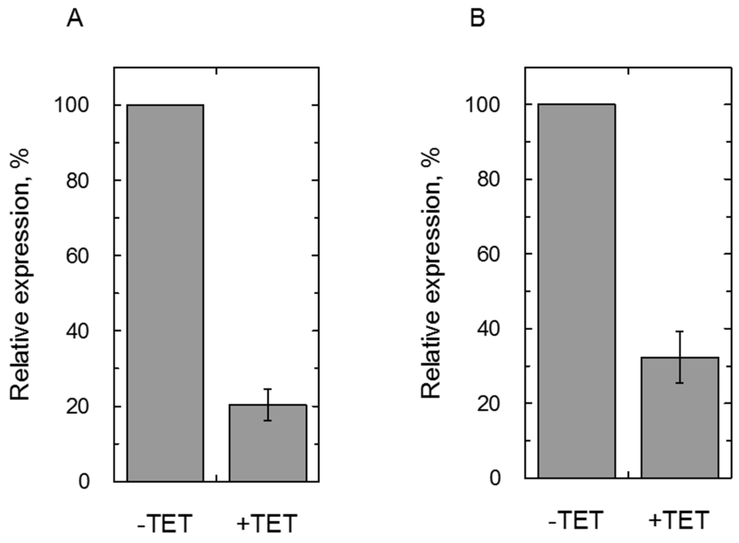

Figure S3. qRT-PCR analysis of transcript levels in Tb927.9.15260 and Tb927.8.6620 knock-down bloodstream trypanosomes. Transcript levels in trypanosomes where RNAi was induced by the addition of tetracycline (1 µg/mL, 24 h) were compared to wild-type (uninduced) levels. Relative levels of cDNA were normalized using the reference gene *TERT* (Tb927.11.10190, telomerase reverse transcriptase).

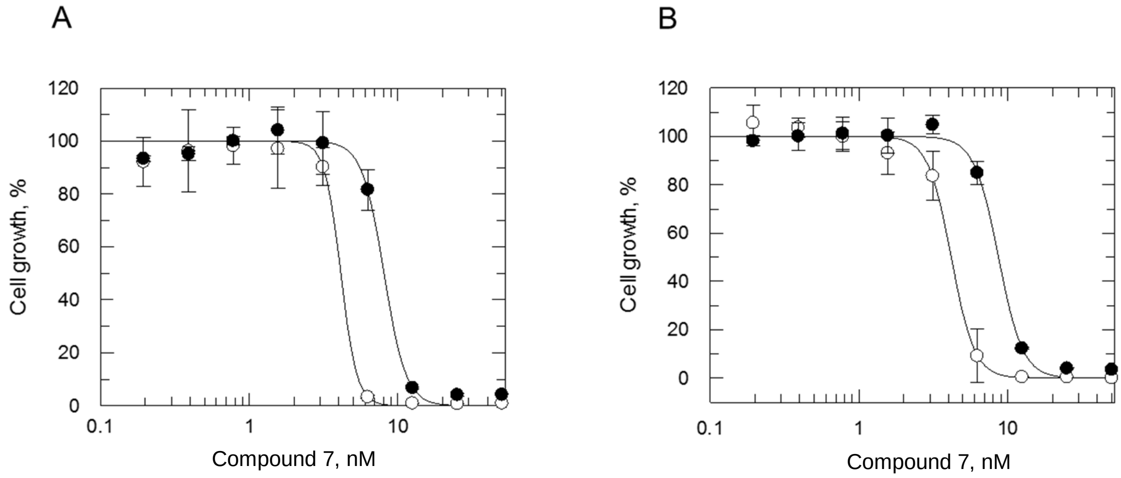

Figure S4. Dose-response curves for compound **7** against cell lines where Tb927.9.15260 (A) and Tb927.8.6620 (B) transcript levels have been knocked-down by RNAi. EC_{50} values were 4.1 ± 0.2 and 8.0 ± 0.4 nM for Tb927.9.15260^{RNAi} uninduced (open circles) and induced (closed circles), respectively. EC_{50} values were 4.2 ± 0.2 and 8.7 ± 0.3 nM for Tb927.8.6620^{RNAi} uninduced (open circles) and induced (closed circles), respectively. Results are the means standard deviations of data from three independent experiments.

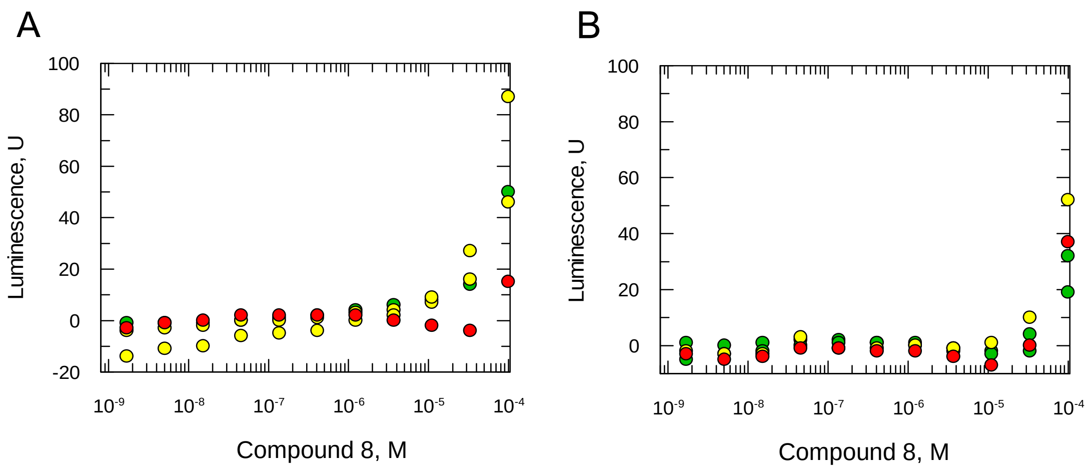

Figure S5. Effect of compound **8** treatment on inhibition of caspase and trypsin-like proteasome activity. Dose-response curves determining the effect of compound **8** treatment on the inhibition of caspase (A) and trypsin-like (B) proteasome activity in lysates of *L. donovani* (green), purified human 26S proteasomes (red) and extracts of THP1 monocytes (yellow).

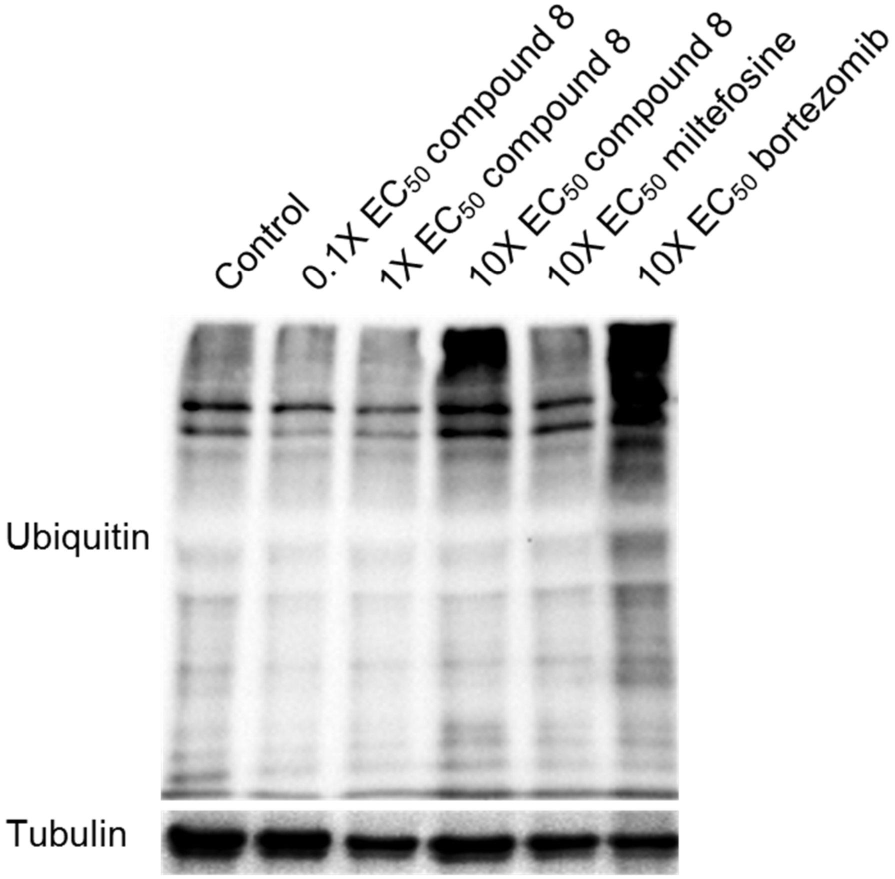

Figure S6. Western blot of ubiquitylated proteins (human antibody α-ubiquitin). 80 µg of each protein sample previously treated or not treated with the different compounds were separated by SDS-PAGE and transferred onto PVDF membranes. Ubiquitin proteins were detected using the human anti-ubiquitin P4D1 antibody diluted 1:1,000. An anti-tubulin antibody 1:1,000 was used as loading control. Compound **8** induces the accumulation of ubiquitylated proteins in the parasite cell in a dose-dependent manner.

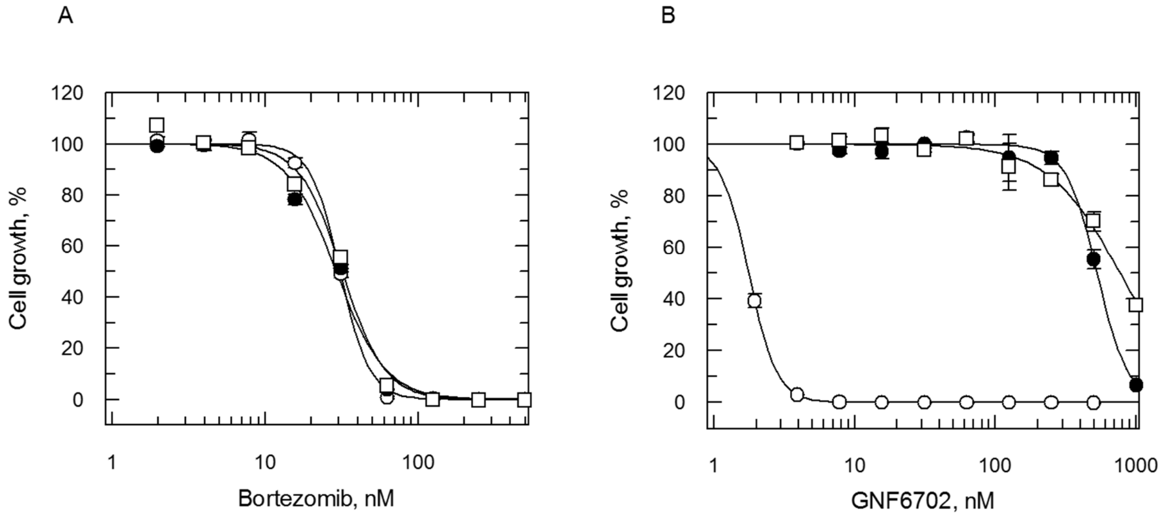

Figure S7. Cross-resistance studies with compound **7**-resistant clonal cell lines. The sensitivity of compound **7**-resistant cloned cell lines (RES I and RES III) to established proteasome inhibitors bortezomib and GNF6702 was determined. Promastigotes resistant to compound **7** were just as sensitive to bortezomib (EC_{50} values of 28 ± 1.4 and 32 ± 1.5 nM for RES I and RES III, respectively) as WT (EC_{50} value of 31 ± 1.5 nM). In contrast, RES I and RES III demonstrated significant levels of cross-resistance to GNF6702 with EC_{50} values of 527 ± 12 and 768 ± 38 nM, respectively compared to 1.8 ± 0.002 nM for WT parasites. WT parasites illustrated by open circles, RES I shown as closed circles and RES III shown as open squares. Data are the mean ± standard deviation of at least two independent experiments. RES II is genetically identical to RES I in having both G197C (β5 subunit) and T30A (β4 subunit) mutations and their response to compounds from this series is similar.

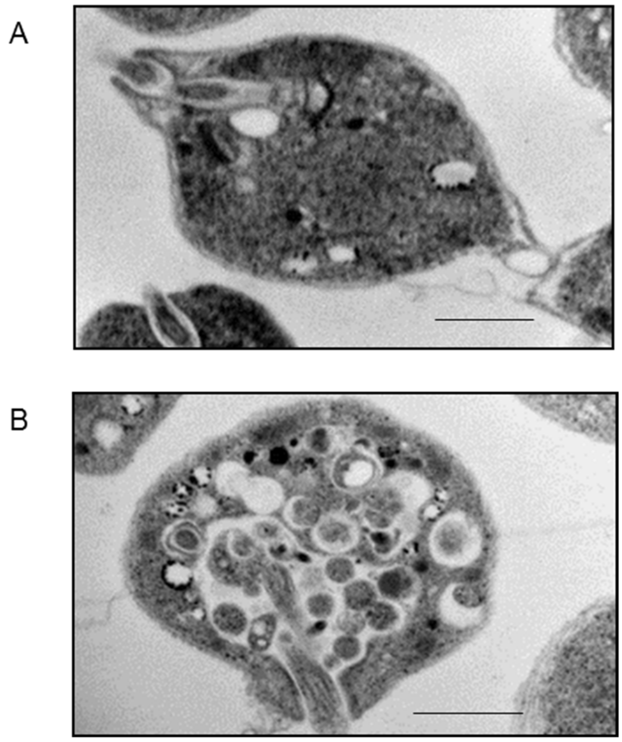

Figure S8. Morphology of **7**-treated *L. donovani* promastigotes. TEM section of untreated promastigotes (A) and promastigotes treated with **7** (2x the established EC_{50} value) for 6h (B). Black bars represent 200 nm.

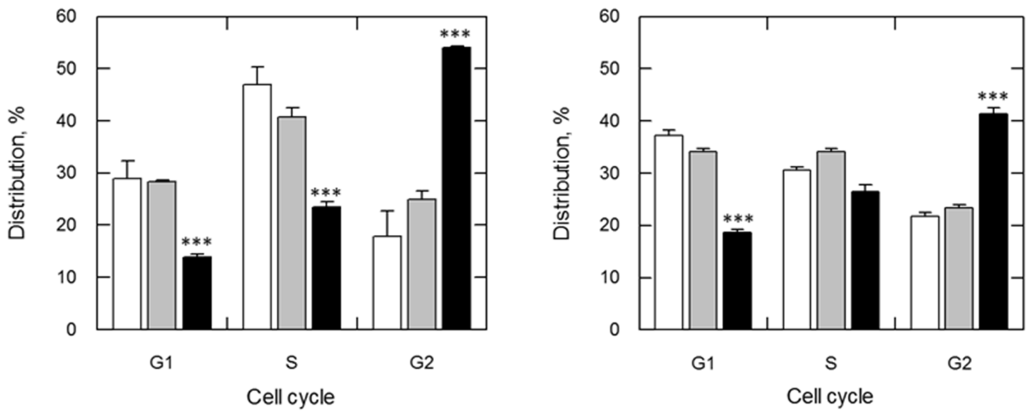

Figure S9. Cell cycle analysis following treatment with compound **7**. Untreated cells at 0 h (white), 6h (grey) and at 8 h (black). Cells were treated with compound **7** equivalent to the respective 5x EC_{50} values. Treatment of *L. donovani* promastigotes and bloodstream *T. brucei* with compound **7** are shown in panels A and B, respectively. Differences in the percentage of drug-treated cells in the G1, S and G2 phases of the cell cycle were confirmed as statistically significant by using an unpaired Student t test (***, P = 0.001).

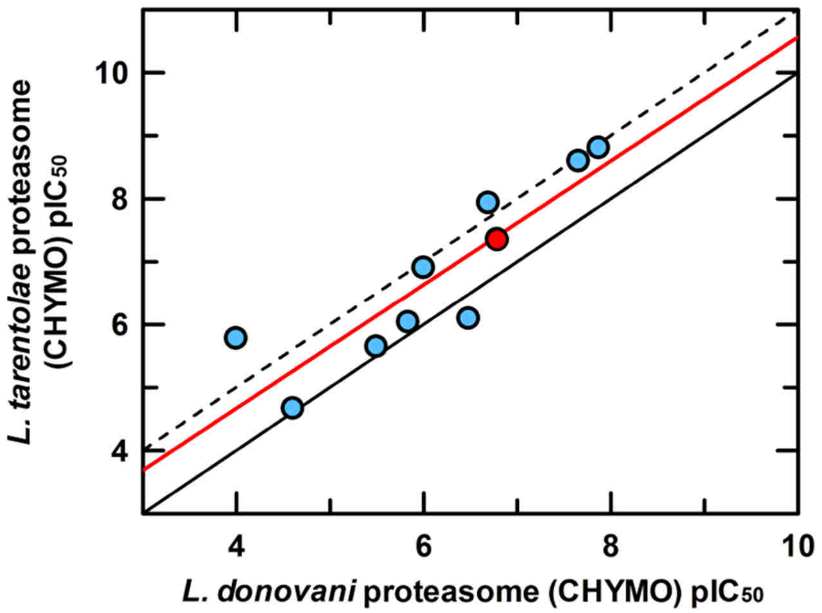

Figure S10. Inhibition of the chymotrypsin-like activities of *L. donovani* and *L. tarentolae* proteasomes by a set of compound **8** analogues. A scatter plot correlating pIC50s of the chymotrypsin-like activities of *L. donovani* (inhibition of purified *L. donovani* proteasome, preventing cleavage of a chymotrypsin peptide, monitored by a Proteasome-Go assay, x-axis) against *L. tarentolae* (inhibition of purified *L. tarentolae* proteasome, preventing cleavage of a Rhodamine R110-labelled chymotrypsin peptide, monitored kinetically by a FLINT assay, y-axis). (1) red line – is the line of best fit, (2) black line – is the x=y line & (3) black dashed line – is the x=y+1 line & (4) red circle is compound **8**.

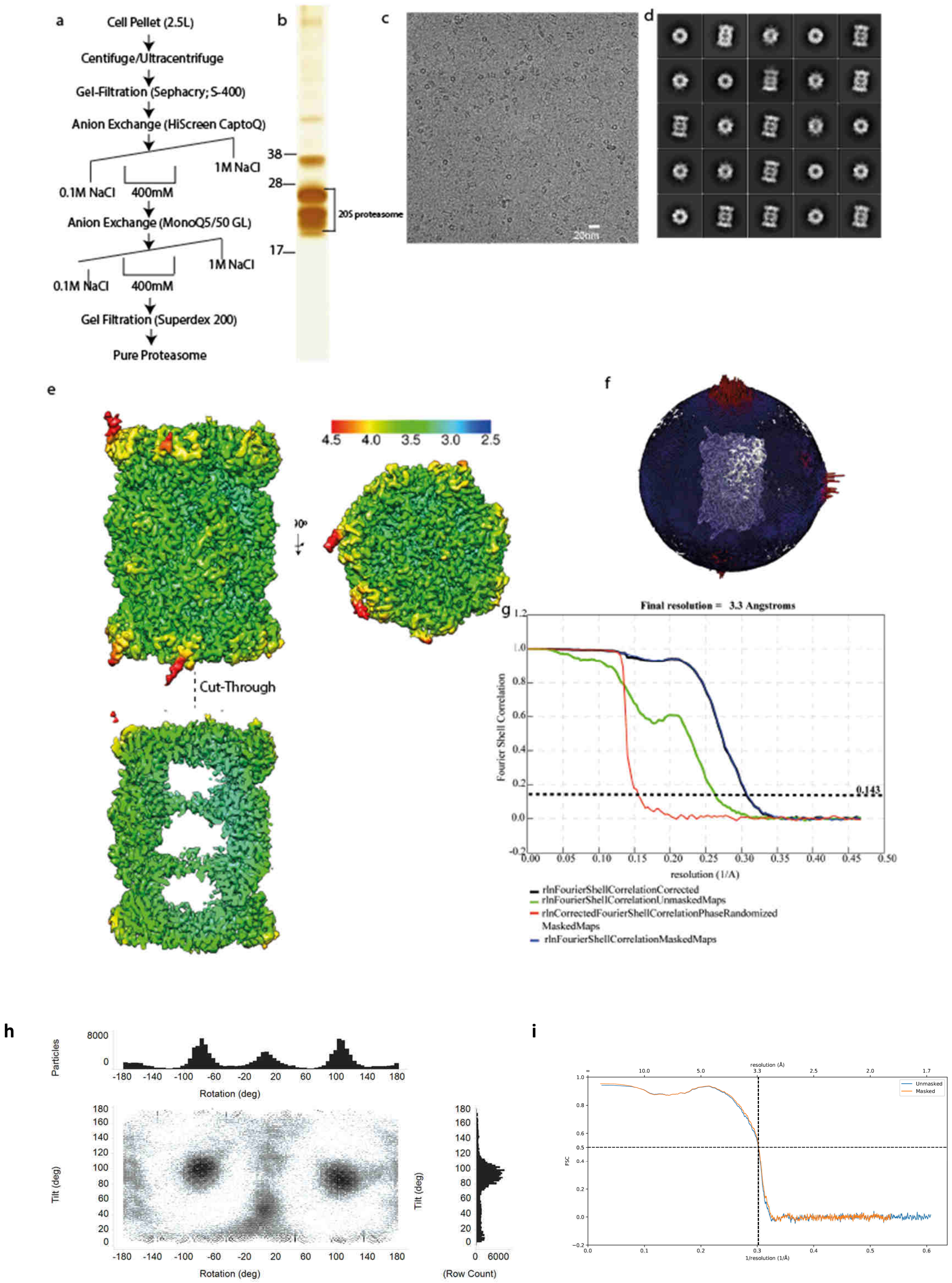

Figure S11. Purification and Cryo-EM single particle data for processing of Apo 20S *L. tarentolae* proteasome. (a) Purification protocol for making homogeneous proteasome for single particle analysis. (b) silver-stained gel of the *L. tarentolae* proteasome from LEXSY system. (c) Representative micrograph. (d) 2D class averages. (e) Local Resolution map (f) Angular distribution (g) Fourier shell correlation indicates a resolution of 3.3 Å according to the 0.143 criterion (h) Angular distribution plot. (i) FSC plot of the model versus map, showing FSC value of 0.5 at 3.3 Å.

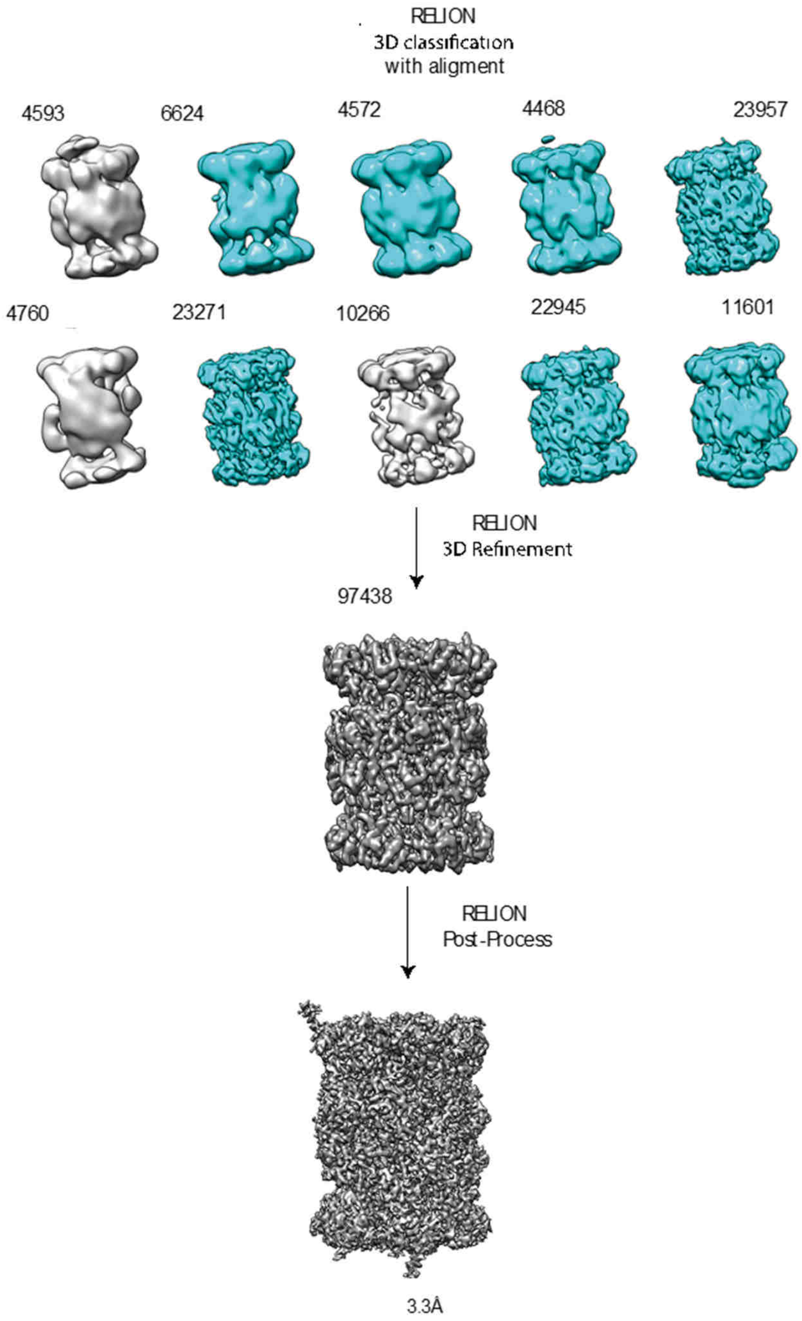

Figure S12. Schematic overview of Cryo-EM image processing for apo *L. tarentolae* 20S proteasome. Particles from 3D classes indicated in cyan were selected for further 3D refinement using EMDB structure-3231.

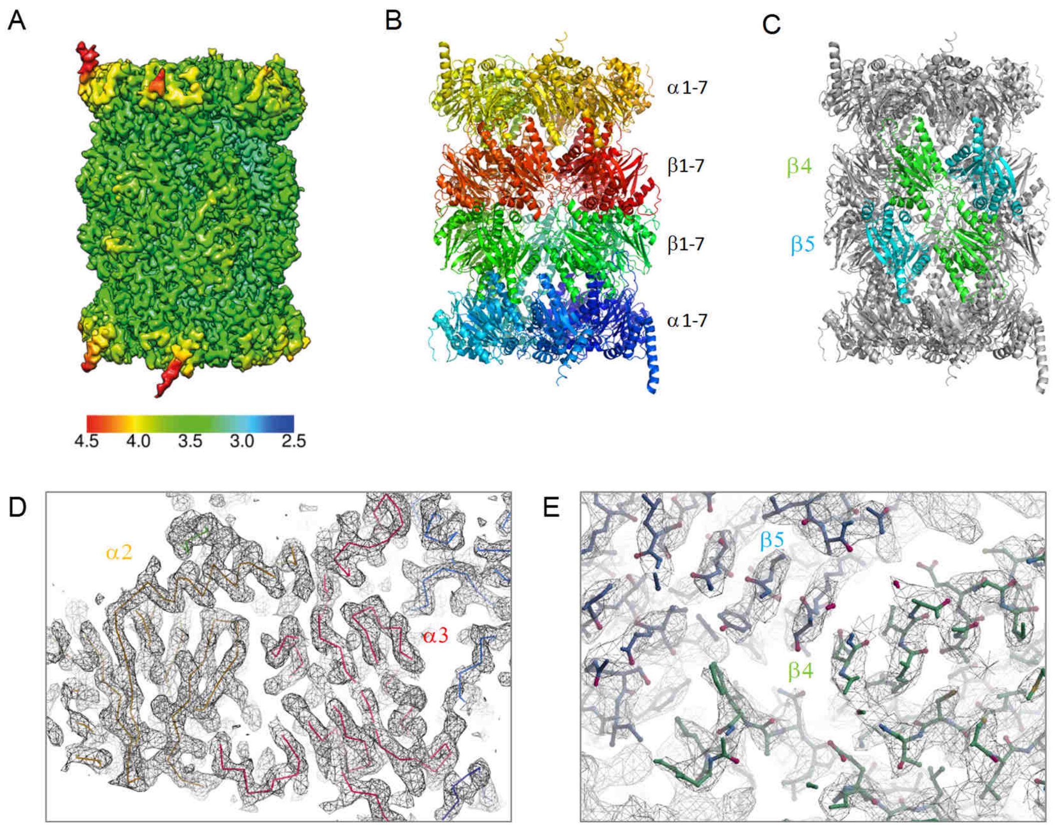

Figure S13. Structure of the *L. tarentolae* 20S proteasome core determined by single particle Cryo-EM at 3.3Å resolution. (A) The Cryo-EM map is surface rendered and colour coded according to local resolution as determined by ResMap. (B) Section of the Cryo-EM map as a mesh representation (contoured at 3.5 sigma) illustrating the quality of the Cryo-EM map. β4 and β5 protein models are in green and blue, respectively. (C) The entire model of 20S shown as a protein cartoon highlighting the layers of alpha and beta subunits. (D) Map at 5 sigma at the α2(Chain B, yellow) and α3 (chain C, red) interface. Only ribbon protein model shown. (E) Map at 4sigma at the β4 (Chain K, green) and β5 (Chain L, blue) interface.

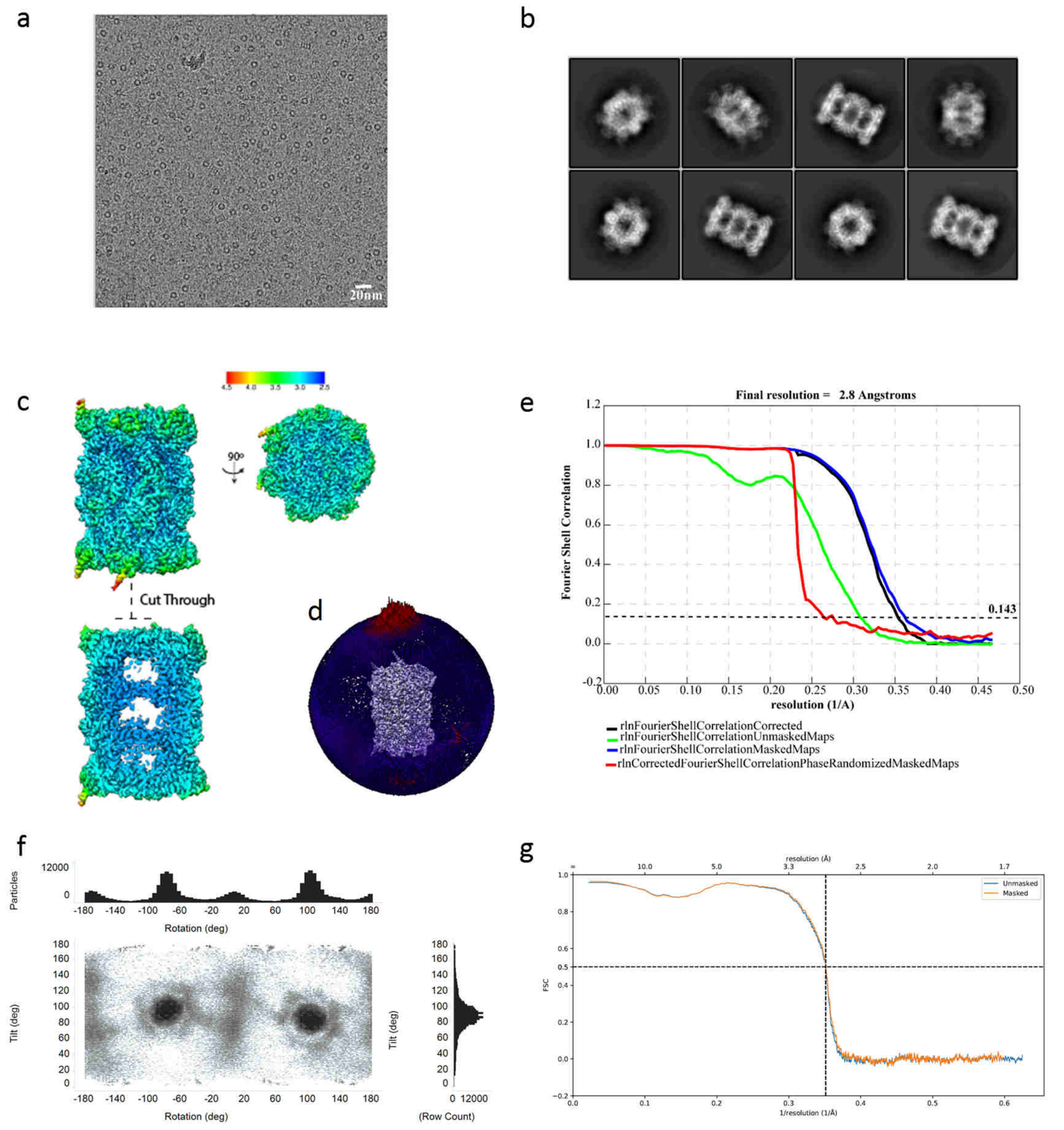

Figure S14. Cryo-EM single particle data processing of compound **8**-liganded 20S *L. tarentolae* proteasome. (a) Representative micrograph of *L. tarentolae* proteasome incubated with compound **8**. (b) Two-dimensional class averages of *L. tarentolae* proteasome with compound 8. (c) Local resolution map. (d) Angular distribution. (e) Fourier shell correlation indicates a resolution of 3.3 Å according to the 0.143 criterion. (f) Angular Distribution plot. (g) FSC plot of the model versus map, showing FSC value of 0.5 at 2.85 Å.

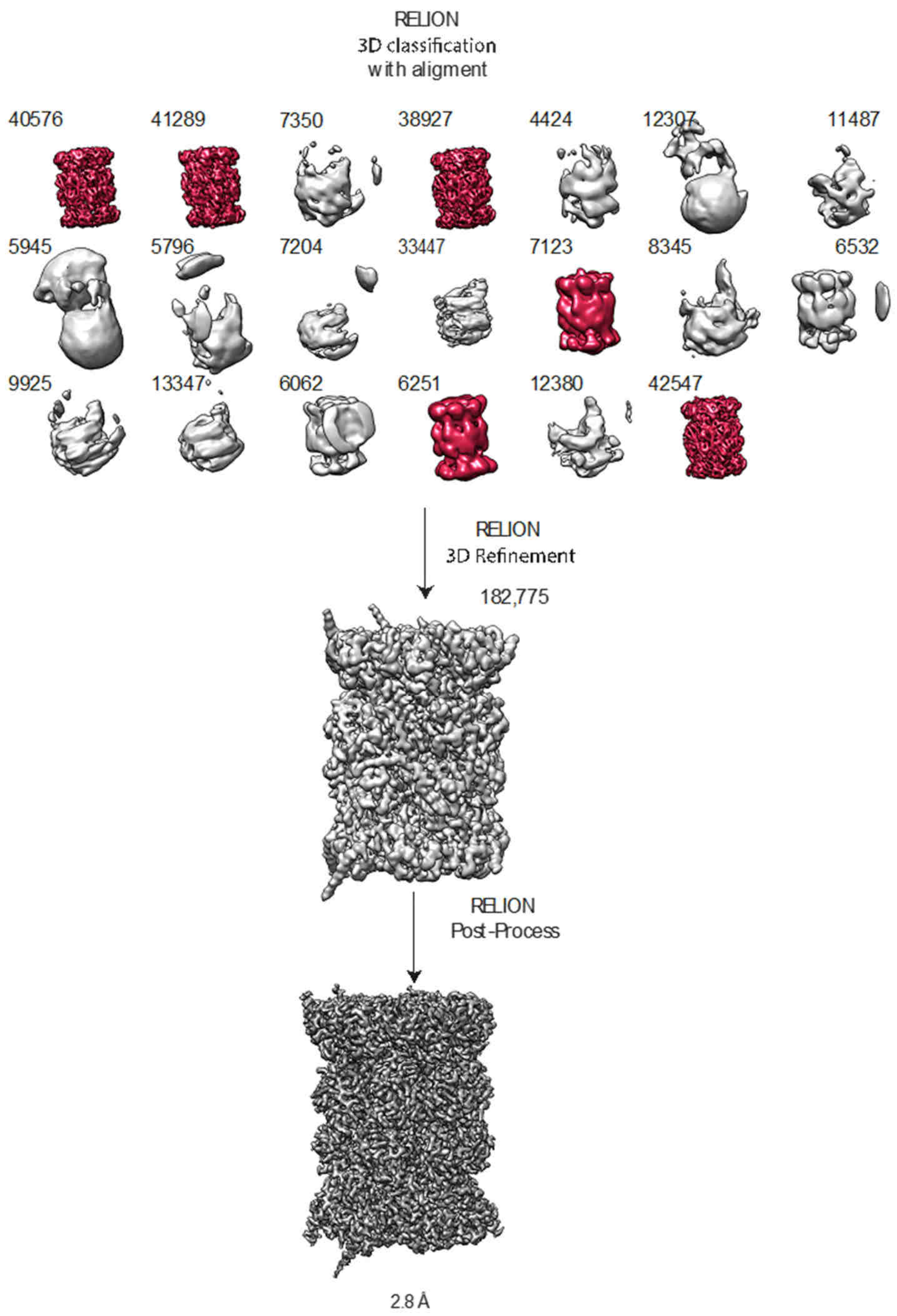

Figure S15. Schematic overview of image processing for *L. tarentolae* 20S proteasome with compound **8**. Particles from 3D classes indicated in red were selected for further 3D refinement using EMD structure-3231.

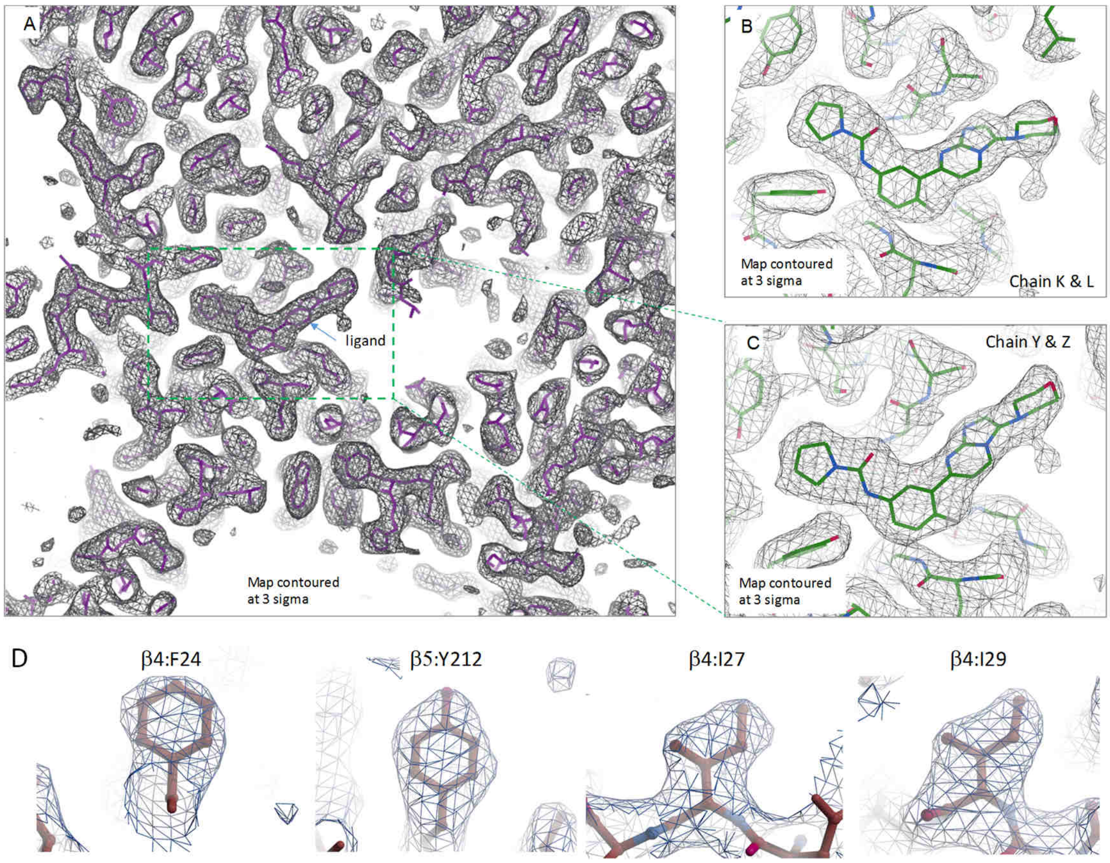

Figure S16. *L. tarentolae* 20S Cryo-EM maps around compound **8** (A) Map and protein model centred on the ligand placed at the interface of chains Y/T (β4/β5). (B) Ligand in chains K/L (β4/β5) can be seen to be well defined by electron map, with an unambiguous binding mode. (C) Ligand in chains Y/T (β4/β5) is also well defined by experimental data and has an identical binding mode that seen in (B). (D) Shows density around some residues proximal to compound **8** in more detail to illustrate the quality of the density can permit phenylalanine and tyrosine to be distinguished as well as the rotameric state of isoleucine.

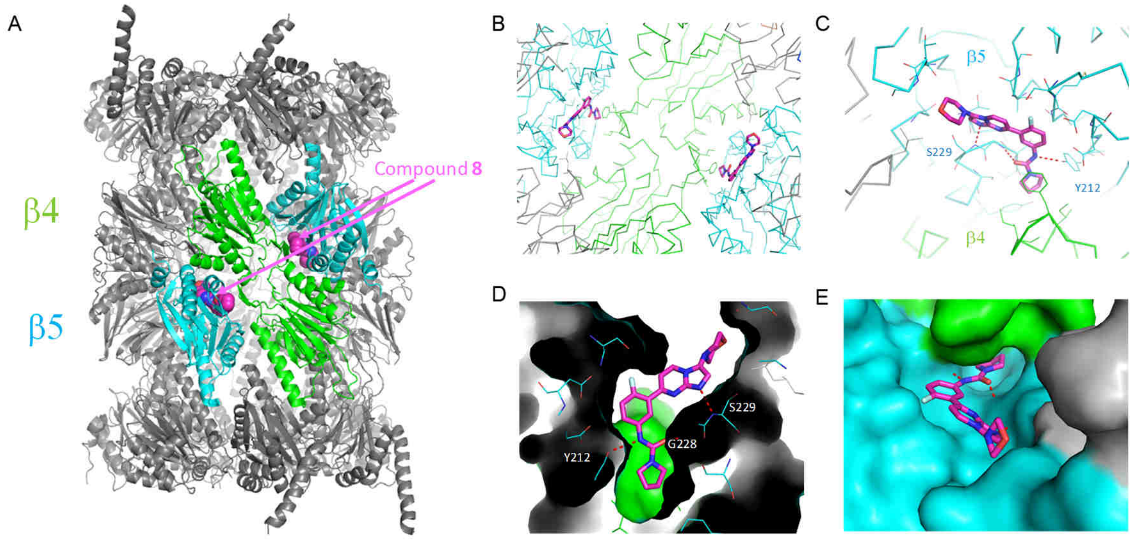

Figure S17. Compound **8** bound to *L. tarentolae* 20S proteasome core. Consistent colouring of 20S proteasome core in grey with β4 (green), β5 (cyan) and compound **8** (magenta), respectively. (A) 20S proteasome shown as a protein cartoon, with compound **8** in a space filled format. (B) Two molecules of compound **8** (stick format), with 20S core as a protein ribbon. (C) Hydrogen bonding interactions of compound **8** (stick format) are made with G228, S229 and Y212. (D) Excellent shape complementarity can be seen in the compound **8** binding pocket. (E) One face of the ligand is bound close to β5 (cyan), the other is largely solvent exposed.

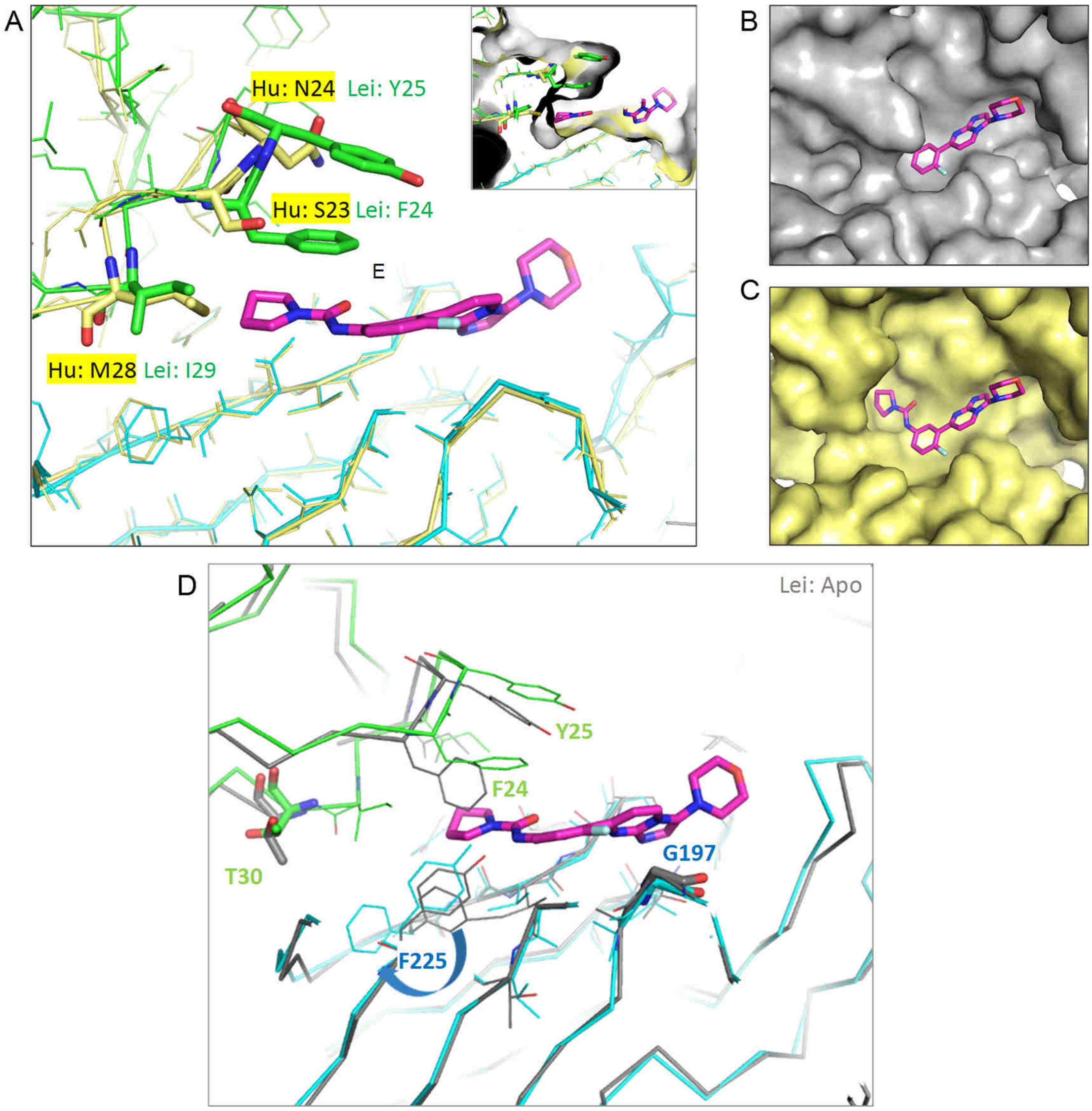

Figure S18. Structural basis of *L. tarentolae* versus human selectivity. Compound **8** bound to *L. tarentolae* and modelled into human 20S proteasome (4r67.pdb). (A) Overlay of human proteasome from 4r67.pdb (yellow) and *L. tarentolae* 20S with β4 (green), β5 (cyan) and compound **8** (magenta) coloured as indicated. Pi-stack formed by F24 and Y25 that covers part of the ligand binding pocket occupied by compound **8** is absent in the human protein. Additionally, the larger M28 residue reduces the size of pyrrolidine pocket. Insert shows surfaces around human (yellow) and *L. tarentolae* protein (grey). (B) Protein surface of the *L. tarentolae* in grey. (C) Protein surface of the human 20S in yellow, with compound **8** modelled into the site. The exposed nature of the site in human protein explains the selectivity between the two species. (D) Interestingly, several of these residues are also those that adopt different conformations on ligand binding, as shown by the overlay of the compound **8** bound structure (cyan/green) with that of the apo *L. tarentolae* 20S in grey.

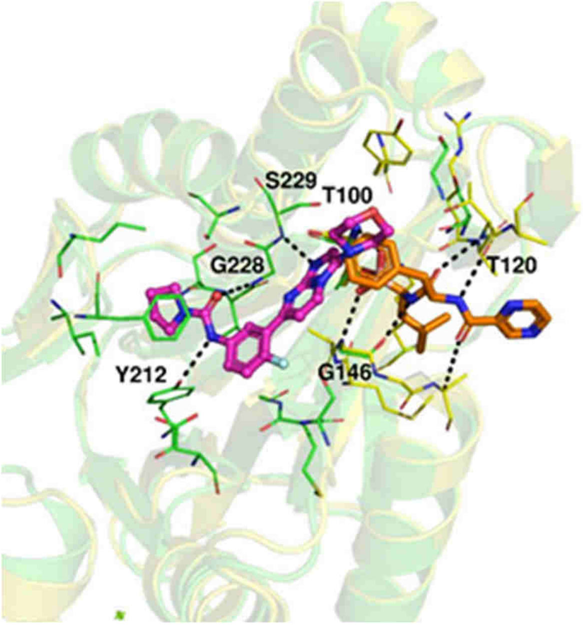

Figure S19. Comparison between binding mode of compound **8** in *L. tarentolae* 20S proteasome (purple ligand and green structure) and bortezomib in the human 20S proteasome (orange ligand and yellow structure – PDB code 5LF3). Residues establishing hydrogen bond interactions (black dashed lines) with the ligands are labelled.

A

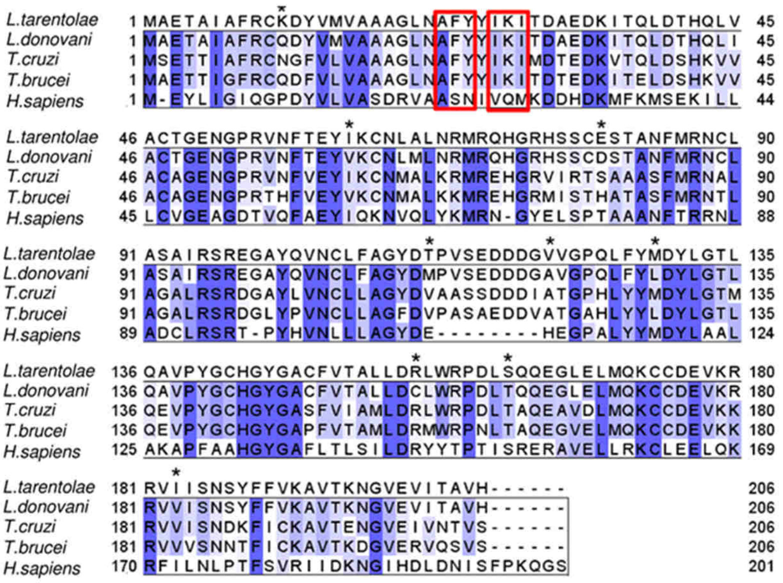

	L.tarentolae	L.donovani	T.cruzi	T.brucei	H.sapiens
**L.tarentolae**	100	100	100	100	17
**L.donovani**	100	100	100	100	17
**T.cruzi**	100	100	100	100	17
**T.brucei**	100	100	100	100	17
**H.sapiens**	17	17	17	17	100

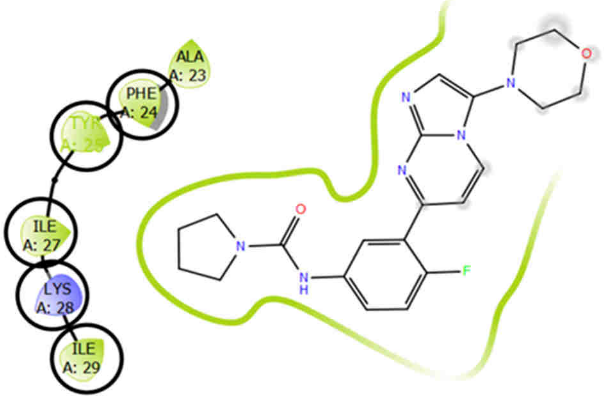

B

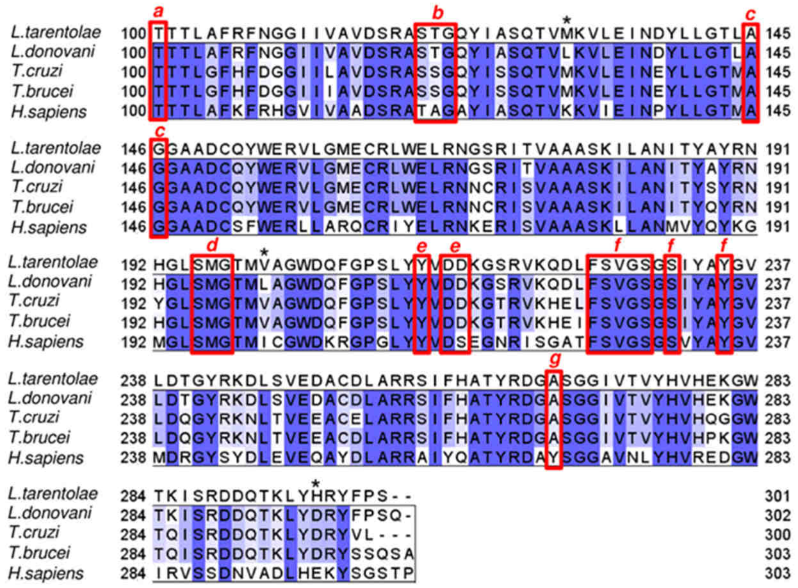

	L.tarentolae	L.donovani	T.cruzi	T.brucei	H.sapiens
**L.tarentolae**	100	100	95	95	80
**L.donovani**	100	100	95	95	80
**T.cruzi**	95	95	100	100	80
**T.brucei**	95	95	100	100	80
**H.sapiens**	80	80	80	80	100

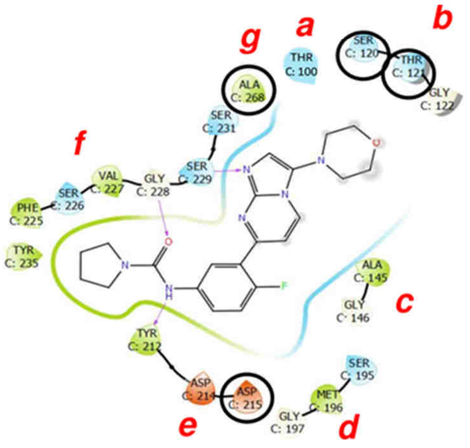

Figure S20. Sequence alignment of *L. tarentolae* with *L. donovani, T. cruzi, T. brucei* and human β4 subunit (A) and β5 subunit (B). The 26 amino acids within 5Å from compound **8** are identified by red boxes. Asterisks indicate positions which have a non-conserved residue in *L. tarentolae* and *L. donovani*. The percentage sequence identity matrix of the binding site region is found below each alignment as defined by the 26 amino acids. On the bottom, 2D diagrams showing the interactions with compound **8**. Circled in black are the 9 residues in the binding site that are different between *L. tarentolae* and human proteasomes.

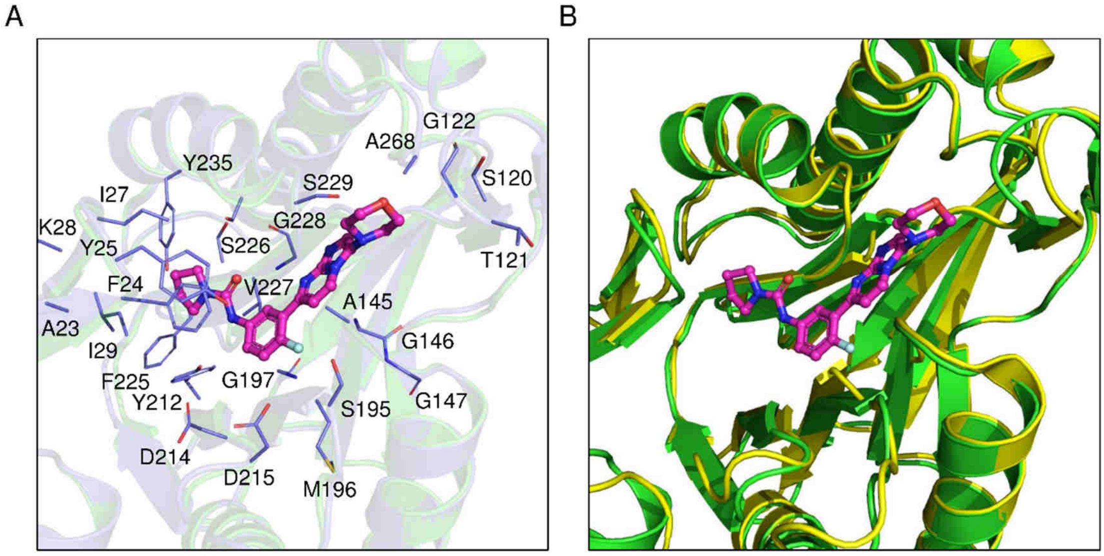

Fig S21. (A) Comparison between the β4-β5 subunits of the *L. tarentolae* 20S proteasome from the Cryo-EM structure (in green) and the β4-β5 subunits of *L. donovani* 20S proteasome from the homology model (in blue). The residues within 5Å from the ligand are displayed. (B) Comparison between the β4-β5 subunits of the *L. tarentolae* 20S proteasome from the Cryo-EM structure (in green) and the β4-β5 subunits of *Human* 20S proteasome (PDB code 5LF3, in yellow).

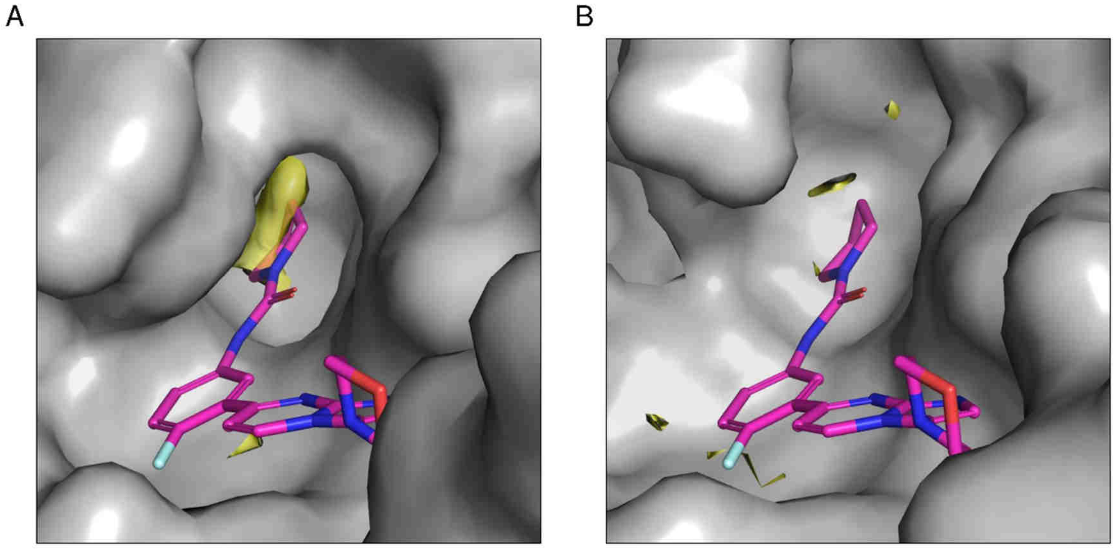

Figure S22. Different morphology of compound **8** binding site. (A) Molecular surface for *L. tarentolae* binding site with compound **8** bound (purple). (B) Molecular surface for human binding site. In yellow, the hydrophobic area matching the pyrrolidine ring, calculated with SiteMap (Schrödinger 2018). In human, due to differences residues in the β4 subunit, the hydrophobic pyrrolidine pocket is lost and the area is solvent exposed.

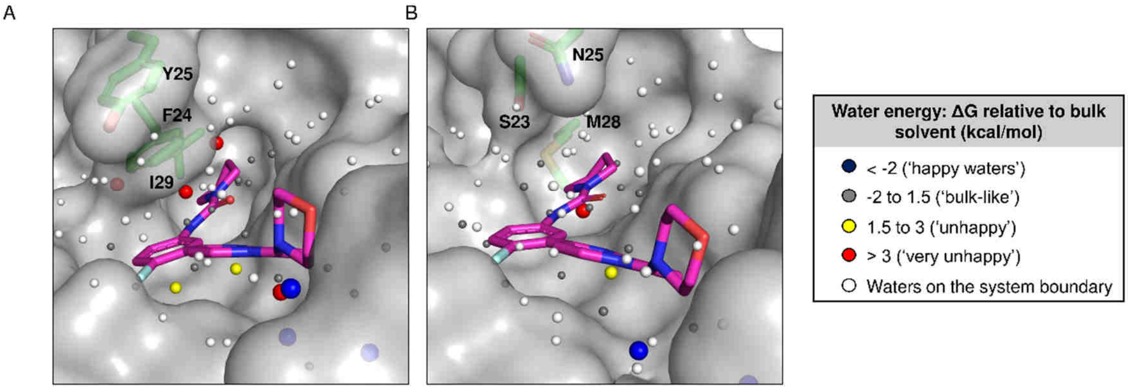

Figure S23. Analysis of the water network in the *L. donovani* (A) and human (B) binding site. Compound **8** is shown in purple. In green, the residues that are responsible for a change in morphology in the pyrrolidine cavity. In the *L. tarentolae* a number of "very unhappy" water molecules are removed upon compound **8** binding. In human the binding site is open to the solvent with a relatively stable water network with only limited stabilisation when the ligand is bound. Water networks and relative energies were calculated using WaterFLAP (Molecular Discovery).

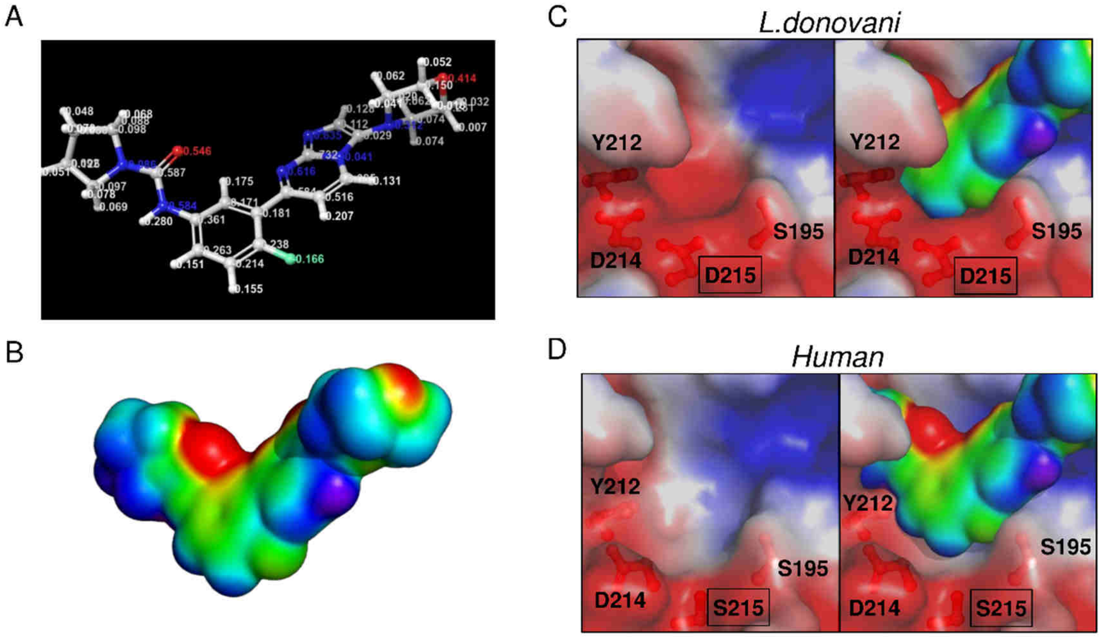

Figure S24. (A and B) atomic charges and molecular electrostatic potential for compound **8** in the *L. tarentolae* Cryo-EM bound conformation. (C and D) Electrostatic surface potential map of β4/β5 interface in *L. donovani* and human structures. On the left the apo binding site, on the right with compound **8** modelled in the binding site. In the electrostatic surface maps, red corresponds to areas negatively charged, blue to positively charged and green neutral.

Supplementary Tables

Table S1. Summary of the activity of compound **8** in clinically-relevant *Leishmania* strains

Strain	*Leishmania* EC_{50} (µM)	Host Cell EC_{50} (µM)
MHOM/ET/67/HU3 (LV9)	0.4 / 0.8	>50
SUKA001	0.5	>50
MHOM/IN/80/DD8	1.6	>50
MHOM/IN/2002/BHU1	0.6	>50
MHOM/MA/67/ITMAP263	7.9 / 1.3	>50

Results from one or two (both values shown) biological replicates.

Table S2. Effect of compound **8** and miltefosine on parasite burden compared to vehicle-treated group. Data represents % of reduction of parasite burden in drug-treated and vehicle control animals calculated from mean LDU data.

**Compound**	**Dosing**	**Reduction in parasite burden, % (compared to the vehicle control group)**
Miltefosine	30 mg/kg u.i.d. (10 days PO)	99.9
Compound **8**	3 mg/kg b.i.d. (10 days PO)	18.4
Compound **8**	10 mg/kg b.i.d. (10 days PO)	56.9
Compound **8**	25 mg/kg b.i.d. (10 days PO)	98.1

Compound **8**	50 mg/kg b.i.d. (10 days PO)	99.1
Compound **8**	25 mg/kg u.i.d. (10 days PO)	8.8

Table S3. Summary of pharmacokinetic parameters for compound **8**. Summary of pharmacokinetic parameters (mean and standard deviation) obtained after intravenous administration at 3 mg/kg single dose or oral administration at 10 mg/kg single dose to CD-1 mouse, SD rat and Beagle dog.

	Mouse CD-1 Male	Rat SD Male	Dog Beagle Male
IV Parameters (D=3 mg/kg iv bolus MO; 3 mg/kg iv bolus RAT; 3 mg/kg iv 0.5 h INF DOG)			
Cl (mL/min/kg)	40.7 ± 13.6	17 ± 5.2	29.6 ± 6.06
(%LBF)	32%	22%	53%
V_{ss} (L/kg)	2.20 ± 0.11	1.0 ± 0.25	1.34 ± 0.381
$t_{1/2}$ (h)	0.65 ± 0.32	0.7 ± 0.14	0.52 ± 0.08
PO Parameters (10 mg/kg po MO-RAT; 10 mg/kg po DOG)			
F (%)	18 (68%)	36 (20%)	46 (29%)

Table S4. Ames testing results for compound **8**.

Ames test without S9-mix

Strain	Compound	Dose level per plate	Mean revertants per plate	Standard Deviation	Ratio treated / solvent	Individual revertant colony counts
**TA100**	Compound **8**	50	99.0	19.8	0.9	113, 85
		150	97.0	5.7	0.9	101, 93

		500	86.0	1.4	0.8	87, 85
		1500	109.5	31.8	1.0	87, 132
		2500	91.5	4.9	0.9	95, 88
		5000	94.5	6.4	0.9	99, 90
	**DMSO**		104.3	11.6		120, 106, 95, 93, 96, 116
**TA1535**	Compound **8**	50	19.0	4.2	0.8	22, 16
		150	15.0	7.1	0.6	20, 10
		500	21.5	9.2	0.8	15, 28
		1500	16.0	0.0	0.6	16, 16
		2500	15.5	3.5	0.6	18, 13
		5000	18.0	8.5	0.7	24, 12
	**DMSO**		25.3	3.4		27, 21, 28, 27, 21, 28
**TA1537**	Compound **8**	50	14.0	5.7	1.0	18, 10
		150	9.5	3.5	0.7	7, 12
		500	16.0	5.7	1.1	20, 12
		1500	12.5	0.7	0.9	13, 12
		2500	14.5	3.5	1.0	17, 12
		5000	6.5	0.7	0.5	7, 6
	**DMSO**		14.3	3.8		13, 20, 11, 13, 11, 18
**TA98**	Compound **8**	50	22.5	9.2	1.1	29, 16
		150	19.0	1.4	0.9	20, 18
		500	14.5	2.1	0.7	16, 13
		1500	19.5	6.4	1.0	15, 24
		2500	16.5	0.7	0.8	16, 17

		5000	18.0	4.2	0.9	21, 15
	**DMSO**		20.3	5.9		23, 26, 15, 27, 18, 13
**WP2 uvrA (pKM101)**	Compound **8**	50	90.0	1.4	0.9	89, 91
		150	84.5	3.5	0.9	82, 87
		500	80.5	2.1	0.8	82, 79
		1500	87.5	16.3	0.9	76, 99
		2500	91.5	2.1	0.9	90, 93
		5000	82.0	2.8	0.8	84, 80
	**DMSO**		97.0	13.5		98, 118, 76, 101, 95, 94
**TA100**	**NaN_3**	2	1050.0	45.3	10.1	1082, 1018
**TA1535**	**NaN_3**	2	1128.5	46.0	44.5	1161, 1096
**TA1537**	**9-AAC**	50	249.5	31.8	17.4	272, 227
**TA98**	**2NF**	1	417.5	23.3	20.5	401, 434
**WP2 uvrA (pKM101)**	**4NQO**	2	4800.5	299.1	49.5	4589, 5012

Key to Positive Controls

NaN_3	Sodium Azide
9-AAC	9-Amino Acridine
2NF	2-Nitrofluorene
4NQO	4-Nitroquinoline-1-oxide

Ames test with S9-mix

Strain	Compound	Dose level per plate	Mean revertants per plate	Standard Deviation	Ratio treated / solvent	Individual revertant colony counts

**TA100**	Compound **8**	50	123.0	1.4	0.9	122, 124
		150	96.5	27.6	0.7	116, 77
		500	106.5	14.8	0.8	96, 117
		1500	118.0	0.0	0.9	118, 118
		2500	101.0	17.0	0.8	89, 113
		5000	122.5	2.1	0.9	124, 121
	**DMSO**		132.0	6.1		121, 137, 134, 137, 129, 134
**TA1535**	Compound **8**	50	12.0	1.4	0.8	11, 13
		150	10.0	1.4	0.6	9, 11
		500	17.5	0.7	1.1	17, 18
		1500	13.5	2.1	0.9	12, 15
		2500	12.5	3.5	0.8	10, 15
		5000	9.0	2.8	0.6	11, 7
	**DMSO**		15.5	3.9		15, 12, 11, 16, 22, 17
**TA1537**	Compound **8**	50	18.0	4.2	0.8	21, 15
		150	14.5	4.9	0.6	18, 11
		500	21.0	4.2	0.9	18, 24
		1500	11.0	2.8	0.5	13, 9
		2500	13.5	2.1	0.6	12, 15
		5000	8.5	4.9	0.4	5, 12
	**DMSO**		22.8	3.7		28, 20, 22, 23, 26, 18
**TA98**	Compound **8**	50	23.0	4.2	1.0	20, 26
		150	25.0	5.7	1.1	29, 21
		500	30.0	9.9	1.3	23, 37

		1500	24.5	3.5	1.0	22, 27
		2500	25.5	17.7	1.1	13, 38
		5000	25.0	2.8	1.1	23, 27
	**DMSO**		23.7	3.7		28, 27, 18, 24, 24, 21
**WP2 uvrA (pKM101)**	Compound **8**	50	150.5	29.0	1.0	171, 130
		150	164.5	19.1	1.1	178, 151
		500	143.5	6.4	1.0	139, 148
		1500	139.0	8.5	0.9	133, 145
		2500	130.5	3.5	0.9	133, 128
		5000	133.5	10.6	0.9	126, 141
	**DMSO**		148.5	5.9		152, 145, 145, 151, 141, 157
**TA100**	**2-AAN**	5	4441.0	99.0	33.6	4371, 4511
**TA1535**	**2-AAN**	5	490.5	0.7	31.6	490, 491
**TA1537**	**2-AAN**	5	153.0	38.2	6.7	126, 180
**TA98**	**B[a]P**	10	471.0	181.0	19.9	343, 599
**WP2 uvrA (pKM101)**	**2-AAN**	10	1650.5	68.6	11.1	1699, 1602

Key to Positive Controls:

2-AAN 2-Aminoanthracene
B[a]P Benzo[a]pyrene

Table S5. Mouse lymphoma assay results for compound **8**.

3 hour +S9 treatment, results summary table

Conc. µg/mL	RTG (%)	MF ($\times 10^{-6}$)	Positive / Negative
0.00	92	107.60	
0.00	111	95.96	
0.00	95	176.19	
375	73	191.25	
410.445	64	222.38	
DMBA	28	1783.48	Positive

Mean Control MF:	126.58

**Key:**			
**RTG**	Relative Total Growth	**DMBA**	3hr +S9 +ve control
**MF**	Mutation Frequency	**MMS**	24hr -S9 +ve control
**P**	Precipitation observed	**1**	Expressed in terms of parent compound

DMBA: 7,12-dimethylbenz[a]anthracene

MMS: methylmethansulfonate

24 hour -S9 treatment, results summary table

Concentration µg/mL	RTG (%)	MF ($\times 10^{-6}$)	Positive / Negative
0.00	95	133.07	
0.00	108	131.89	
0.00	97	168.87	
40	90	75.89	

70	46	78.36	
100	19	86.96	
MMS	73	938.58	Positive

Mean Control MF:	144.61

**Key:**			
**RTG**	Relative Total Growth	**DMBA**	3hr +S9 +ve control
**MF**	Mutation Frequency	**MMS**	24hr -S9 +ve control
**P**	Precipitation observed	**1**	Expressed in terms of parent compound

DMBA: 7,12-dimethylbenz[a]anthracene

MMS: methylmethansulfonate

Table S6. Primers used in target identification studies.Upper case letters refer to nucleotides corresponding to gene sequences in *T. brucei* or *L. donovani*; lower case refers to additional sequences used in generating constructs. Restriction endonuclease sites are underlined.

Primer name	Primer sequence
LIB2f	5′-TAGCCCCTCGAGGGCCAGT-3´
LIB2r	5′-GGAATTCGATATCAAGCTTGGC-3′
Tb15260-F	5´- gatcctcgagggtaccCACATAAAACGCCAGTGTGG -3´
Tb15260-R	5´- gatctctagaggatccCTCACTCTCCAGGAGCAACC -3´
Tb6620-F	5´- gatcgggcccggtaccTCTTTTCTCGCCTTCGTTGT -3´
Tb6620-R	5´- gatctctagaggatccACTGTGGGACTCTGACCACC -3´
qPCR15260-F	5´- TTCCCCCACCCTTTTCATCG -3´
qPCR15260-R	5´- GGCCGGTTCCCCAAATATCA -3´
qPCR6620-F	5´- GTGGTCAGAGTCCCACAGTG -3´

qPCR6620-R	5´- CCTGATGACCACAACCCGAA -3´
qPCR-*TERT*-F	5´- AGGAACTGTCACGGAGTTTGC-3´
qPCR-*TERT*-R	5´- AGGAACTGTCACGGAGTTTGC -3´
Ldβ4 seq-F	5´- CAGACCCGCTCACAAGATTTC -3´
Ldβ4 seq-R	5´- CCAACCTCACCCCGTTCAC -3´
Ldβ5 seq-F	5´- CAAGATTTCCTCAAGTGCGTGC -3´
Ldβ5 seq-R	5´- GGTGGAGGCGCCAGCAC -3´

Table S7. Genome-wide RNAi library screening of compound **7**. Table summarising the genes within the ubiquitin-proteasome degradation pathway (green) identified as playing a role in compound resistance following screening of the RITseq library in *T. brucei*.

**Gene ID**	**Gene name**
Tb927.9.15260	ubx domain containing protein, putative
Tb927.8.6620	uba containing protein , conserved
Tb927.10.4980	ubiquitin-like protein DSK2, putative (DSK2)
Tb927.5.780	hypothetical protein, conserved
Tb927.6.2460	ubiquitin fusion degradation protein 2
Tb927.9.5260	ERAD-associated E3 ubiquitin-protein ligase HRD1, putative
Tb927.10.7790	ubiquitin fusion degradation protein, putative
Tb11.v5.0553	ubiquitin hydrolase, putative
Tb927.8.6330	WD domain, G-beta repeat/PFU (PLAA family ubiquitin binding)
Tb927.6.1670	ubiquitin hydrolase, pseudogene, putative, cysteine peptidase
Tb927.8.1590	ubiquitin-protein ligase, putative (upl3)

Table S8. MALDI–TOF analysis of UbiQ-018-labelled *L. donovani* proteins. Proteasome-enriched fractions of L. donovani lysates were labelled with the human proteasome active-site probe Me4BodipyFL-Ahx3Leu3VS, commercially known as UbiQ-018. Labelled samples were then separated by electrophoresis on a 15% SDS-PAGE acrylamide gel. Following separation, gels were scanned in the fluorescein channel of a ChemiDoc reader (λ excitation/emission = 480/530 nm). Fluorescently

labelled proteins were excised from the gel (see Figure 4C) and MALDI–TOF analysis carried out by the FingerPrints Proteomics service at the University of Dundee. Identity of the recovered peptides detailed below. Catalytically active subunits of the *Leishmania* proteasome are highlighted in red.

Gel fragment	Peptide	UniProt ID	Function
Lower band	1	Q868B1	40S ribosomal protein S5
	2	Q4QBH2	tryparedoxin peroxidase
	3	Q4Q5P2	60S ribosomal protein L17, putative
	4	Q4QG98	60S ribosomal protein L18, putative
	5	Q4QHC3	small GTP-binding protein Rab11, putative
	6	Q9TZS4	tryparedoxin peroxidase
	7	Q4Q1Q9	proteasome β-5 subunit
	8	Q4Q5J4	60S ribosomal protein L18a
	9	Q4QAA9	60S ribosomal protein L12, putative
	10	Q4Q2R6	40S ribosomal protein S19 protein, putative
Upper band	1	Q4Q5K7	RNA binding protein, putative
	2	P25204	40S ribosomal protein S8, putative
	3	Q4Q271	stress-inducible protein STI1 homolog
	4	Q4Q4A0	40S ribosomal protein S3, putative
	5	Q4Q3B9	60S ribosomal protein L13a, putative
	6	P48157	60S ribosomal protein L11
	7	E9ACZ6	proteasome alpha 7 subunit
	8	Q4QDX9	60S ribosomal protein L10a
	9	Q4QG98	60S ribosomal protein L18
	10	Q4QFY6	pyrroline-5-carboxylate reductase
	11	O97213	60S ribosomal protein L10
	12	Q4QF84	60S ribosomal protein L6, putative
	13	E9AFK8	proteasome β-2 subunit, putative

Table S9. Statistics for data collection, 3D reconstruction and model refinement.

**Hardware**	
Microscope	D3512
Detector (mode)	Falcon III (Counting)
Accelerating voltage	300 kV
Detector Pixel size (Å)	1.07

**Illumination parameters**	
Gun Lens	5
Spot size	10
Illuminated area	1.38 µm

**Dose**	
Nominal Magnification	75000
Square pixel (Å^2)	1.07 x 1.07
Dose per physical pixel per second	0.50
Exposure time (seconds)	60
Number of Fractions	75

Number of Frames	31
Total dose (e/Å^2)	30

**Apertures (size in microns)**	
C1	2000
C2	50
C3	2000
Objective	100

**EPU parameters**	
Defocus range (-m)	-1.8, -2.0, -2.2, -2.4, -2.6, -2.8, -3.0, -3.2, -3.4
Autofocus Frequency	10 µm
Drift measurement	None
Delay after stage shift	5 sec
Delay after image shift	3.5 sec
Exposures per hole	1

3D Reconstruction

	**Apo-Proteasome**	**Liganded complex**
Total Micrographs	2896*(2 sessions, identical settings)	2189
Particles for final refinement	97,438	182,775
Resolution of final map (Å)	3.3	2.8
Map sharpening B-factor	-152.035	-112.99

Model Refinement

	Apo structure	Liganded structure
Refined resolution (Å)	3.3	2.8
R-factor	0.278	0.254
Average Fourier shell correlation	0.864	0.866
R.m.s. deviations		
Bond lengths (Å)	0.014	0.012
Bond angles (°)	1.91	1.87
Ramachandran plot		
Favoured (%)	91.7	95.7
Allowed (%)	7.4	3.9

Outliers (%)	0.9	0.4
MolProbity all-atom clash score	4.6	5.3
Mean B-factors (Å^2)		
Protein atoms	58.0	43.2
Ligand atoms	-	37.8
Waters	-	14.8

Ethical Statements

- Rat pharmacokinetics and *in vivo* efficacy: All regulated procedures, at the University of Dundee, on living animals was carried out under the authority of a project licence issued by the Home Office under the Animals (Scientific Procedures) Act 1986, as amended in 2012 (and in compliance with EU Directive EU/2010/63). Licence applications will have been approved by the University's Ethical Review Committee (ERC) before submission to the Home Office. The ERC has a general remit to develop and oversee policy on all aspects of the use of animals on University premises and is a sub-committee of the University Court, its highest governing body.

- Rat toxicology studies and mouse and dog pharmacokinetics: All animal studies were reviewed by GSK's internal ethical review committee and performed in accordance with Animals (Scientific Procedures) Act 1986 and the GSK Policy on the Care, Welfare, and Treatment of Laboratory Animals (UK 1986).

- The human biological samples were sourced ethically and their research use was in accord with the terms of the informed consents. Usage of human-sourced macrophages was approved by the "Scottish National Blood Transfusion Service committee for the governance of blood and tissue samples for non-therapeutic use, and donor research".

REFERENCES

[1] Synthesis of compound **2** described in US2011053915A.

[2] Synthesis of 2-amino-5-morpholinopyrimidine described in WO 2008018529.

[3] Robinson MW, Hill AP, Readshaw SA, Hollerton JC, Upton RJ, Lynn SM, Besley SC, Boughtflower BJ (2017) Use of Calculated Physicochemical Properties to Enhance Quantitative Response When Using Charged Aerosol Detection, *Anal. Chem., 89* (3), 1772–1777.

[4] De Rycker M, Thomas J, Riley J, Brough SJ, Miles TJ, Gray DW (2016) Identification of Trypanocidal Activity for Known Clinical Compounds Using a New *Trypanosoma cruzi* Hit-Discovery Screening Cascade. *PloS Negl. Trop. Dis. 10*(4): e0004584.

[5] De Rycker M, Hallybruton I, Thomas J, Campbell L, Wyllie S, Joshi D, Cameron S, Gilert IA, Wyatt PG, Frearson JA, Fairlamb AH, Gray DW (2013) Comparison of a high-throughput high-content intracellular *Leishmania donovani* assay with an axenic amastigote assay. *Antimicrob. Agents Chemother. 57*, 2913-2922.

[6] Nühs A, De Rycker M, Manthri S, Comer E, Scherer CA, Schreiber SL, Ioset JR, Gray DW (2015) Development and Validation of a Novel *Leishmania donovani* Screening Cascade for High-Throughput Screening Using a Novel Axenic Assay with High Predictivity of Leishmanicidal Intracellular Activity. *PLoS Negl. Trop. Dis. 9*, e0004094.

[7] Wyllie S, Fairlamb AH (2006) Refinement of techniques for the propagation of *Leishmania donovani* in hamsters. *Acta Tropica*, *97*(3):364-369.

[8] Escobar P, Yardley V, Croft SL (2001) Activities of Hexadecylphosphocholine (Miltefosine), AmBisome, and Sodium Stibogluconate (Pentostam) against *Leishmania donovani* in Immunodeficient scidMice. *Antimicrob. Agents Chemother. 45:* 1872-1875.

[9] Seifert K, Croft SL (2006) *In Vitro* and *In Vivo* Interactions between Miltefosine and Other Antileishmanial Drugs. *Antimicrob. Agents Chemother. 50*: 73-79.

[10] Jiménez-Antón MD, García-Calvo E, Gutiérrez C, Escribano MD, Kayali N, Luque-García JL, Olías-Molero AI, Corral MJ, Costi MP, Torrado JJ, Alunda JM (2018) Pharmacokinetics and disposition of miltefosine in healthy mice and hamsters experimentally infected with *Leishmania infantum*. *Eur. J. Pharm. Sci*. *121*:281-286.

[11] http://www.dndi.org/diseases-projects/diseases/vl/tpp/tpp-vl.html

[12] Croft SL, Snowdon D, Yardley V (1996)The activities of four anticancer alkyllysophospholipids against *Leishmania donovani*, *Trypanosoma cruzi* and *Trypanosoma brucei*. *J. Antimicrob. Chemother*. *38*: 1041-1047.

[13] Bradley DJ, Kirkley J (1977) Regulation of Leishmania populations within the host. I. the variable course of *Leishmania donovani* infections in mice. *Clin. Exp. Immunol*. *30*: 119-129.

[14] Bacterial Reverse Mutation Test. OECD Guideline for the Testing of Chemicals, Test Guideline 471; 1997.

[15] Maron DM, Ames BN (1983) Revised methods for the Salmonella mutagenicity test. *Mutat. Res. 3-4*: 173-215.

[16] *In Vitro* Mammalian Cell Gene Mutation Test. OECD Guidelines for the Testing of Chemicals. Test Guideline 476, 1997.

[17] Clive D, Johnson KO, Spector JFS, Batson AG, Brown MMM (1979) Validation and characterisation of the L5178Y/TK$^{+/-}$ Mouse Lymphoma Mutagen Assay system. *Mutation Research 59*(1):61-108.

[18] Clive D, Bolcsfoldi G, Clements J, Cole J, Homna M, Majeska J, Moore M, Muller L, Myhr B, Oberly T, Oudelhkim M-C, Rudd C, Shimada H, Sofuni T, Thybaud V, Wilcox P (1995) Consensus agreement regarding protocol issues discussed during the mouse lymphoma workshop: Portland, Oregon, may 7, 1994 *Environ. and Mol. Mutag. 25*(2): 165-168.

[19] Cole J et al. Gene mutation assays in cultured mammalian cells. In: Kirkland DJ, Gatehouse DG (1990) Basic mutagenicity tests : UKEMS recommended procedures : UKEMS Sub-Committee on Guidelines for Mutagenicity Testing : report, part 1 Cambridge, UK:Cambridge University Press, 87-114.

[20] Moore MM, Clive D, Hozier JC, Howard BE, Batson AG, Turner NT, Sawyer J. (1985) Analysis of trifluorothymidine-resistant (TFTr) mutants of L5178Y/TK± mouse lymphoma cells *Mutat. Res. 151*(1):161-174.

[21] Goyard S, Segawa H, Gordon J, Showalter M, Duncan R, Turco SJ, Beverley SM (2003) An in vitro system for developmental and genetic studies of *Leishmania donovani* phosphoglycans. *Mol. Biochem. Parasitol*. *130*(1):31-42.

[22] Glover L, Alsford S, Baker N, Turner DJ, Sanchez-Flores A, Hutchinson S, Hertz-Fowler C, Berriman M, Horn D (2015) Genome-scale RNAi screens for high-throughput phenotyping in bloodstream-form African trypanosomes. *Nature Protoc. 10*: 106-133.

[23] Greig N, Wyllie S, Patterson S, Fairlamb aH (2009) A comparative study of methylglyoxal metabolism in trypanosomatids. *FEBS J. 276(2): 376–386.*

[24] Langmead B, Salzberg SL (2012) Fast gapped-read alignment with Bowtie 2. *Nat. Methods 9*: 357-359.

[25] Li H, Handsaker B, Wysoker A, Fennell T, Ruan J, Homer N, Marth G, Abecasis G, Durbin R; 1000 Genome Project Data Processing Subgroup (2009) The Sequence Alignment/Map format and SAMtools. *Bioinformatics 25*: 2078-2079.

[26] Carver T, Harris SR, Berriman M, Parkhill J, McQuillan JA (2012) Artemis: an integrated platform for visualization and analysis of high-throughput sequence-based experimental data. *Bioinformatics 28*: 464-469.

[27] Wyllie S, Roberts AJ, Norval S, Patterson S, Foth BJ, Berriman M, Read KD, Fairlamb AH (2016) Activation of Bicyclic Nitro-drugs by a Novel Nitroreductase (NTR2) in *Leishmania*. *PloS Pathogens 12* (11), e1005971.

[28] Redmond S, Vadivelu J, Field MC (2003) RNAit: an automated web-based tool for the selection of RNAi targets in *Trypanosoma brucei*. *Mol. Biochem. Parasitol*. *128*(1):115-118.

[29] Alsford S, Kawahara T, Glover L, Horn D (2005) Tagging a *T. brucei* RRNA locus improves stable transfection efficiency and circumvents inducible expression position effects. *Mol. Biochem. Parasitol*. *144*(2):142-8.

[30] Burkard G, Fragoso CM, Roditi I (2007) Highly efficient stable transformation of bloodstream forms of *Trypanosoma brucei*. *Mol. Biochem. Parasitol*. *153*(2):220-223.

[31] van den Hoff MJ, Moorman AF, Lamers WH (1992) Electroporation in 'intracellular' buffer increases cell survival. *Nucleic Acids Res*. *20*(11):2902.

[32] Broadhead R, Dawe HR, Farr H, Griffiths S, Hart SR, Portman N, Shaw MK, Ginger ML, Gaskell SJ, McKean PG, Gull K (2006) Flagellar motility is required for the viability of the bloodstream trypanosome. *Nature 440*: 224–227.

[33] Tu X, Wang CC (2005) Coupling of Posterior Cytoskeletal Morphogenesis to the G1/S Transition in the *Trypanosoma brucei* Cell Cycle. *Mol. Biol. Cell 16*: 97–105.

[34] Kisselev AF, Goldberg AL (2005) Monitoring activity and inhibition of 26S proteasomes with fluorogenic peptide substrates. *Methods Enzymol. 398*: 364-378.

[35] Khare S, Nagle AS, Biggart A, Lai YH, Liang F, Davis LC, Barnes SW, Mathison CJ, Myburgh E, Gao MY, Gillespie JR, Liu X, Tan JL, Stinson M, Rivera IC, Ballard J, Yeh V, Groessl T, Federe G, Koh HX, Venable JD, Bursulaya B, Shapiro M, Mishra PK, Spraggon G, Brock A, Mottram JC, Buckner FS, Rao SP, Wen BG, Walker JR, Tuntland T, Molteni V, Glynne RJ, Supek F (2016) Proteasome inhibition for treatment of leishmaniasis, Chagas disease and sleeping sickness. *Nature 537*(7619):229-233.

[36] Zheng SQ, Palovcak E, Armache JP, Verba KA, Cheng Y, Agard DA (2017) MotionCor2: anisotropic correction of beam-induced motion for improved cryo-electron microscopy. *Nat. Methods 14*, 331-332.

[37] Fernandez-Leiro R, Scheres SH (2016) Unravelling biological macromolecules with cryo-electron microscopy. *Nature 537*, 339-346

[38] Rohou A, Grigorieff N (2015) CTFFIND4: Fast and accurate defocus estimation from electron micrographs. *J. Struct. Biol. 192*, 216-221.

[39] Pettersen EF, Goddard TD, Huang CC, Couch GS, Greenblatt DM, Meng EC, Ferrin TE (2004).UCSF Chimera--a visualization system for exploratory research and analysis. *J. Comput. Chem. 25*, 1605-1612.

[40] Murshudov GN, Skubák P, Lebedev AA, Pannu NS, Steiner RA, Nicholls RA, Winn MD, Long F, Vagin AA (2011) *REFMAC5* for the refinement of macromolecular crystal structures. *Acta Cryst. D67*, 355-367.

[41] Winn MD, Ballard CC, Cowtan KD, Dodson EJ, Emsley P, Evans PR, Keegan RM, Krissinel EB, Leslie AGW, McCoy A, McNicholas SJ, Murshudov GN, Pannu NS, Potterton EA, Powell HR, Read RJ, Vagin A, Wilson KS (2011) Overview of the CCP4 suite and current developments. *Acta Crystallogr D Biol Crystallogr. 67*(Pt 4): 235–242.

[42] McCoy AJ, Grosse-Kunstleve RW, Adams PD, Winn MD, Storoni LC, Read RJ (2007) Phaser crystallographic software. *J. Appl. Cryst. 40*: 658-674.

[43] Emsley P, Lohkamp B, Scott WG, Cowtan K (2010) Features and development of Coot. *Acta Cryst. D 66*: 486-501.

[44] Smart OS, Holstein J, Womack TO, Sharff A,Flensburg C, Keller P, Paciorek W, Vonrhein C, Bricogne G (2016) GRADE version 1.2.12. Global Phasing Ltd., Cambridge, U.K.

[45] Schrödinger Small Molecule Drug Discovery suite. Release 2018-2: Schrödinger, LLC, New York, NY, 2018.

[46] Case D, Darden TA, Cheatham TE, Simmerling CL *AMBER 16. 2016*.

[47] Frisch MJ et al. (2004) *Gaussian 03, Revision C.02* (Gaussian, Inc., Wallingford CT).
